# Analysis of the Sequences, Structures, and Functions of Product-Releasing Enzyme Domains in Fungal Polyketide Synthases

**DOI:** 10.3389/fmicb.2017.01685

**Published:** 2017-09-04

**Authors:** Lu Liu, Zheng Zhang, Chang-Lun Shao, Chang-Yun Wang

**Affiliations:** ^1^Key Laboratory of Marine Drugs, The Ministry of Education of China, School of Medicine and Pharmacy, Ocean University of China Qingdao, China; ^2^Laboratory for Marine Drugs and Bioproducts, Qingdao National Laboratory for Marine Science and Technology Qingdao, China; ^3^State Key Laboratory of Microbial Technology, School of Life Sciences, Shandong University Jinan, China; ^4^Institute of Evolution and Marine Biodiversity, Ocean University of China Qingdao, China

**Keywords:** fungal polyketide synthase, product-releasing enzyme, sequence, structure, function

## Abstract

Product-releasing enzyme (PRE) domains in fungal non-reducing polyketide synthases (NR-PKSs) play a crucial role in catalysis and editing during polyketide biosynthesis, especially accelerating final biosynthetic reactions accompanied with product offloading. However, up to date, the systematic knowledge about PRE domains is deficient. In the present study, the relationships between sequences, structures, and functions of PRE domains were analyzed with 574 NR-PKSs of eight groups (I–VIII). It was found that the PRE domains in NR-PKSs could be mainly classified into three types, thioesterase (TE), reductase (R), and metallo-β-lactamase-type TE (MβL-TE). The widely distributed TE or TE-like domains were involved in NR-PKSs of groups I–IV, VI, and VIII. The R domains appeared in NR-PKSs of groups IV and VII, while the physically discrete MβL-TE domains were employed by most NR-PKSs of group V. The changes of catalytic sites and structural characteristics resulted in PRE functional differentiations. The phylogeny revealed that the evolution of TE domains was accompanied by complex functional divergence. The diverse sequence lengths of TE lid-loops affected substrate specificity with different chain lengths. The volume diversification of TE catalytic pockets contributed to catalytic mechanisms with functional differentiations. The above findings may help to understand the crucial catalysis of fungal aromatic polyketide biosyntheses and govern recombination of NR-PKSs to obtain unnatural target products.

## Introduction

A great variety of fungal aromatic polyketides have an important impact on the pharmaceutical industry and agricultural production due to a wide range of biological products including clinical drugs as well as undesirable toxins and virulence factors (Hertweck, 2009; Crawford and Townsend, 2010; Chooi and Tang, 2012). Fungal aromatic polyketides are produced by non-reducing polyketide synthases (NR-PKSs), belonging to type I PKSs characterized by multidomain monomodular megasynthases (Crawford et al., 2008; Chooi and Tang, 2012; Xu et al., 2013b). According to the phylogeny and domain architectures, NR-PKSs have been categorized into eight major groups (groups I–VIII) by our recent study (Liu et al., 2015). Most of these eight groups contain the domain architectures of starter unit: ACP transacylase (SAT), ketosynthase (KS), malonyl-CoA:ACP transacylases (MAT), product template (PT), acyl-carrier protein (ACP), and product-releasing related domains such as thioesterase (TE), reductase (R), and metallo-β-lactamase-type TE (MβL-TE) (Chooi and Tang, 2012; Liu et al., 2015).

**FIGURE 1:**
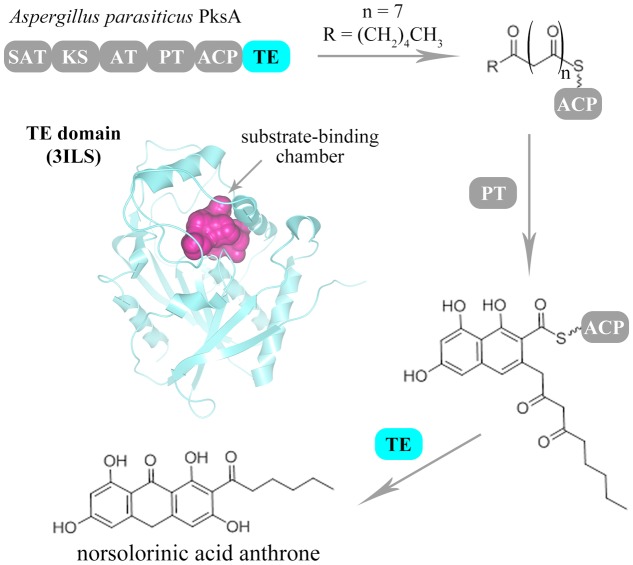
Biosynthesis scheme of fungal aromatic polyketides catalyzed by NR-PKSs. The catalytic process of fungal NR-PKSs is exemplified by *A. parasiticus* PksA (Q12053). The PT domain of PksA catalyzes the regio-selective cyclization of linear polyketide intermediates. Then, the PRE (TE/CLC) domain of PksA catalyzes the last ring cyclization of the aromatic polyketide intermediates and off-load the mature polyketide products. The catalytic pocket of TE/CLC structure (PDB code: 3ILS) of PksA is indicated with purple color.

As a class of crucial domains, the product-releasing related domains have been demonstrated to dominate the final step reaction to synthesize and release the products in many NR-PKSs (Crawford and Townsend, 2010; Chooi and Tang, 2012; Newman et al., 2014). It is essential that the reactions catalyzed by product-releasing related domains rapidly drive and stably maintain the overall biosynthetic program (Newman et al., 2014). In the past decades, several product-releasing related genes have been cloned and characterized, including TE, R, and MβL-TE, of which TE and R are C-terminal domains (Du and Lou, 2010). During fungal polyketide biosynthesis, most final polyketide intermediates are precisely transformed and released by TE domains (Korman et al., 2010). Recently, the TE domains have been proven to perform not only biosynthetic roles but also editing functions (Korman et al., 2010; Vagstad et al., 2012a; Newman et al., 2014). The undesirable derailment products could be effectively eliminated by TE-mediated editing role for maintaining the biosynthetic fidelity (Vagstad et al., 2012a; Newman et al., 2014). Furthermore, the flexible substrate specificity and intermediate chain length control could be precisely checked in each catalytic cycle of polyketide extension by TE domains (Vagstad et al., 2012a; Newman et al., 2014). Besides the TE domains, the R domains have also been found to facilitate the reductive product release in some NR-PKSs containing C-methyltransferase (CMeT) domains (Zabala et al., 2012). Except the C-terminal product-releasing related domains, the physically discrete MβL-TE domains have been found to be encoded in the vicinity of NR-PKSs in group V and release products by hydrolysis or fourth-ring cylization (Lim et al., 2012). So far, only one product-releasing related domain crystal structure has been reported from *Aspergillus parasiticus* PksA in group IV, which is a TE domain participating in aflatoxin B1 biosynthesis (Korman et al., 2010). Generally, during the biosynthesis of aromatic polyketides by NR-PKSs, the linear polyketide intermediates were regio-selectively cyclized by PT domains to form the polyketides containing one or two aromatic rings. Then, these aromatic polyketide intermediates were further processed and released by product-releasing related domains (**Figure 1**).

According to an earlier phylogenetic analysis of NR-PKSs involved in melanin or conidial pigment biosynthesis, it demonstrated that the TE domains with the same known functions were grouped within a clade (Vagstad et al., 2012b). Later, based on a phylogenetic tree of NR-PKSs from *Aspergillus nidulans*, the product-releasing related domains including R domains were marked corresponding to NR-PKSs in groups I, III–VII (Yeh et al., 2013). Recently, the functions of TE domains were reported to be differentiated into hydrolase and cross-coupling activities, especially diverse Claisen-like cyclase (CLC) and transferase activities, according to phylogenetic analysis of TE sequences of NR-PKSs from ascomycetes and basidiomycetes (Lackner et al., 2013). However, none of phylogenetic analyses involved all of product-releasing related domains in fungal NR-PKSs from eight groups.

Until now, the systematic knowledge about product-releasing related domains is insufficient. The relationships between sequences, structures, and functions of the diverse product-releasing related domains have not been illustrated. In this study, for the purpose of describing conveniently, the product-releasing related domains were collectively called as product-releasing enzyme (PRE) domains. The differentiations of PRE domains were labeled in the phylogenetic tree of 574 NR-PKSs involved in groups I–VIII. The three-dimensional structures of different PRE domains were modeled, and the relationships between catalytic sites, structural characteristics, and catalytic mechanisms were analyzed and compared. Specifically, the reasons that TE domains perform diverse functions were discussed with variations on the differentiations of sequences and structures. The complex and interesting evolution tracks of PRE domains in fungal NR-PKSs were also described according to the relationships between sequences, structures, and functions.

## Materials and Methods

### Dataset

The amino acid sequences of 58 fungal NR-PKSs with known product release mechanisms were collected from NCBI database (GenBank; Benson et al., 2013) with accession numbers which were obtained from the literature. For 58 fungal NR-PKSs, the homologous sequences of each were searched and obtained by BLAST individually (Johnson et al., 2008). Then, the amino acid sequences (a total of 574) were selected from the merged homologous sequences of 58 fungal NR-PKSs by eliminating the repetitive sequences and partial sequences. The accession numbers and related information of NR-PKSs were provided in Supplementary Tables S1, S2. The PRE sequences of NR-PKSs were extracted and calibrated with SMART (Letunic et al., 2012) and CDD (Marchler-Bauer et al., 2013).

### Phylogenetic Analysis

The NR-PKS sequences and PRE sequences were aligned with MAFFT (Katoh and Standley, 2013). Phylogenetic analyses were conducted using MEGA version 6 by the bootstrap neighbor joining method (Tamura et al., 2013). The evolutionary distances were computed using the Poisson correction method and were in the units of the number of amino acid substitutions per site. The phylogenetic tree was displayed by iTOL (Letunic and Bork, 2011).

### Structure Modeling

The three-dimensional models of PRE domains were constructed using comparative protein modeling method by I-TASSER (Yang et al., 2015). All the structural models were refined in the atomic-level by the fragment-guided molecular dynamics (FG-MD) simulations (Zhang et al., 2011). The quality assessment of Ramachandran plot has been used to quantitatively assess the accuracy of protein structure predictions. The statistical data of Ramachandran plot was calculated by PROCHECK (Laskowski et al., 1993).

### Lid Region, Cavity Volume, and Cavity Lining Residue Site Analyses

The structural mapping and pocket architecture visualization were displayed using VMD (Humphrey et al., 1996). The lid and loop sequences of TE domains were extracted and calibrated with structural information and multiple sequence alignment. The normalized B-factor was predicted in ResQ (Yang et al., 2016). The TE-lid sequences were aligned with MAFFT (Katoh and Standley, 2013). The cavity volumes and cavity lining residue (CLR) sites were analyzed by CASTp (Dundas et al., 2006) and CAVER (Pavelka et al., 2016).

### Evolutional Conservation Analysis

The evolutionary conservation of amino acid positions in the PRE sequences was estimated by using ConSurf algorithm (Ashkenazy et al., 2010). The LG substitution matrix and computation were based on the empirical Bayesian paradigm. Conservation scale was defined from the most variable amino acid positions (grade 1, color represented by turquoise) which were considered as rapidly evolving to conservative positions (grade 9, color represented by maroon) which were considered as slowly evolving. Sequence logos were generated as graphical representations of the multiple sequence alignment of the amino acids (Crooks et al., 2004).

## Results

### PRE Domains in Phylogenetic Tree of Fungal NR-PKSs

**FIGURE 2:**
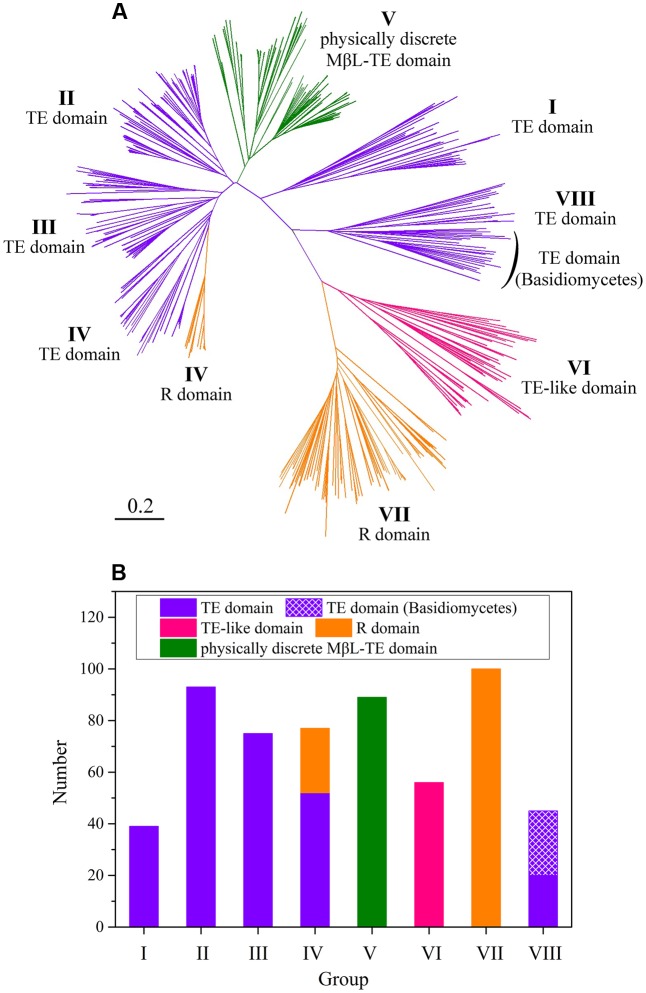
The NR-PKS phylogenetic tree and analysis of PRE domains in 574 NR-PKS sequences. **(A)** NR-PKS phylogenetic tree of 574 sequences. The different types of PRE domains in NR-PKSs have been colored. The branches of NR-PKSs in eight groups (groups I–VIII) have been labeled. The tree is drawn to scale, with branch lengths in the same units as those of the evolutionary distances used to infer the phylogenetic tree. **(B)** Statistical analysis of PRE domains in 574 NR-PKSs. The sequence numbers of NR-PKSs in groups I–VIII are 39, 93, 75, 77, 89, 56, 100, and 45, respectively.

The fungal NR-PKSs amino acid sequences for analysis of PRE domains were searched and screened from the NCBI database. The selected 574 amino acid sequences derived from both ascomycetes and basidiomycetes constituted a fungal NR-PKS dataset (Supplementary Table S1). The NR-PKS phylogenetic tree was constructed on the basis of the phylogeny and domain architectures. The resulting NR-PKS phylogenetic tree (**Figure 2A**) clearly classified the collected sequences into eight major groups with NR-PKSs from basidiomycetes only appeared in group VIII, which was consistent with our previous study (Liu et al., 2015).

The various PRE domains were labeled on the derived NR-PKSs in phylogenetic tree of fungal NR-PKSs (**Figure 2A**). It could be found that there are mainly three types of PRE domains, TE, R, and MβL-TE. The majority of TE and R domains located at the C-terminus of NR-PKSs, with 304 sequences containing TE domains and 125 sequences including R domains. Most PRE domains of NR-PKSs in groups I–III, VI, and VIII were characterized as TE or TE-like domains (**Figure 2B**). The sequence lengths of PRE domains of NR-PKSs in group VI, approximately 350 amino acid residues, were obviously longer than that of NR-PKSs in groups I–IV and VIII containing 250–300 residues. Considering the unusual sequence lengths, we proposed that the PRE domains of NR-PKSs in group VI could be defined as “TE-like” domains. About two-thirds PRE domains of NR-PKSs in group IV were TE domains, while the others were R domains. All PRE domains of NR-PKSs in group VII were identified with R domains. In addition, the physically discrete MβL-TE domains were employed by most NR-PKSs in group V, which do not possess the C-terminal fused PRE domains.

### Functional Differentiations of PRE Domains

**FIGURE 3:**
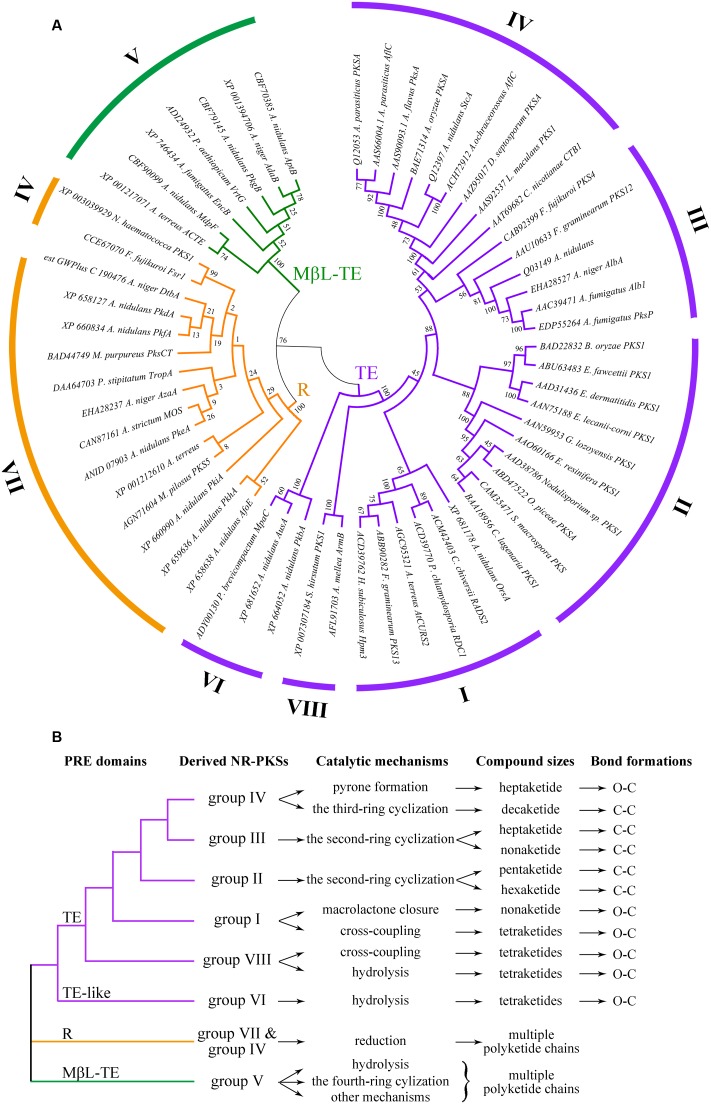
Phylogenetic tree of fungal PRE domains and their functional differentiations. **(A)** Phylogenetic tree of PRE domains of selected 58 NR-PKSs. PRE domains of 58 NR-PKSs are related to three types of product release mechanisms. Phylogenetic analysis was conducted using the bootstrap neighbor joining method. The tree is drawn to scale, with branch lengths in the same units as those of the evolutionary distances used to infer the phylogenetic tree. **(B)** Functional differentiations of fungal PRE domains.

The amino acid sequences of 58 NR-PKSs were selected according to the criterion of NR-PKS whose product release modes has been reported in the literature (Supplementary Table S2). These 58 NR-PKSs were found to cover all known groups (groups I–VIII) of fungal NR-PKSs. Successively, the PRE phylogenetic tree from these 58 NR-PKSs was established based on their product release modes (**Figure 3A**). In order to describe the phylogeny of PRE domains, the PRE phylogenetic tree was constructed with the PRE sequences with the product release functions, mainly including TE and R domains. Besides these two domains, MβL-TE was also involved due to its product release functions, despite it evolved independently. The resulting PRE phylogenetic tree clearly classified the PRE sequences into mainly three distinct types corresponding with TE, R, and MβL-TE. The phylogenetic relationships of diverse TE and TE-like domains were corresponding with that of their derived NR-PKSs (groups I–IV, VI, and VIII). The TE-like domains of NR-PKSs in group VI were arisen in early evolution from TE domains. Notably, the R domains mostly from NR-PKSs in groups IV and VII converged into a separate clade and the MβL-TE recruited by NR-PKSs in group V formed another one. The product release functions of PRE domains and their derived NR-PKS groups were precisely labeled in **Figure 3B** and **Supplementary Figure S1**. It should be clarified that different from the previous reports (Vagstad et al., 2012b; Lackner et al., 2013; Yeh et al., 2013), in the present study, the TE-like and MβL-TE domains were included in the phylogenetic analysis of PRE domains. Furthermore, the phylogenetic analysis of PRE sequences in our study involved TE, R, MβL-TE, and TE-like domains derived from all of the NR-PKS groups I–VIII reported by now. The relationships between different PRE domains, catalytic mechanisms, compound sizes, and bond formations were also demonstrated based on the phylogenetic analysis of PRE sequences.

The TE and TE-like domains, involved in NR-PKSs of six groups (groups I–IV, VI, and VIII), present more complex phylogenetic relationships and various product release mechanisms than other two types. The product release functions of TE and TE-like domains could be generally differentiated into two manners, O–C bond formation and C–C bond formation (**Figure 3B** and **Supplementary Figure S1**). It should be noted that TE and TE-like domains from these six NR-PKS groups are characterized not only various release mechanisms but also different chain lengths of polyketide intermediates (**Figure 3B**).

The O–C bond formations in TE and TE-like domains in NR-PKSs of groups I, IV, VI, and VIII involved four catalytic mechanisms, including macrolactone closure, cross-coupling, pyrone formation, and hydrolysis. The TE domains with intramolecular macrolactone closure function are from the NR-PKSs in group I, exemplified by hypothemycin Hpm3 (Reeves et al., 2008), radicicol RDC1 (Zhou et al., 2010), and zearalenone PKS13 (Wang et al., 2009). Most macrolactone rings closed by TE domains are composed with 14 members, except the 12-membered macrolactone ring catalyzed by TE domain of *Aspergillus terreus* AtCURS2 (Xu et al., 2013a). The TE domains with macrolactone closure function perform high stereotolerance and macrocyclize both D and L configured synthetic substrate analogs (Heberlig et al., 2014). Most TE domains with intermolecular cross-coupling activity are involved in the NR-PKSs of group VIII, such as *Armillaria mellea* ArmB (Lackner et al., 2013) and *Stereum hirsutum* FP-91666 SS1 PKS1 (Lackner et al., 2012). Interestingly, besides catalyzing macrocyclization, the TE domain of zearalenone PKS13 in group I also catalyzes the cross coupling of a benzoyl thioester with alcohols and amines (Wang et al., 2009). Similarly, the TE domain of *A. nidulans* OrsA in group I catalyzes an intermolecular transesterification during biosynthesis of lecanoric acid (Gressler et al., 2015). Besides cross-coupling activity, the TE domains of NR-PKSs in group VIII often perform hydrolysis activity to produce orsellinic acid (Lackner et al., 2013; Yu et al., 2016). The complex phylogeny revealed that the evolution of TE domains was accompanied by functional divergence from hydrolysis to cross-coupling to macrolactone closure. These findings reflect the immanent functional differentiation relationships of TE and TE-like domains between the NR-PKSs in groups I, VI, and VIII. Currently, only one case of pyrone formation has been reported among fungal TE domains. The TE domain of *Cercospora nicotianae* CTB1 in group IV catalyzes O13–C1 bond closure of a heptaketide intermediate (Newman et al., 2012). In addition, the TE-like domains of NR-PKSs in group VI catalyze tetraketide release by hydrolysis (Lo et al., 2012), which is distantly relate to TE domains of NR-PKSs in groups I–IV and VIII.

The TE domains of NR-PKSs in groups II–IV have been shown catalytic activities of CLCs by catalyzing C–C ring closure reactions. The regio-selective cyclizations of TE/CLC domains could be divided into two modes, the second-ring cyclization and the third-ring cyclization. All TE/CLC domains of NR-PKSs in groups II and III catalyze the formation of the second ring through C10–C1 Claisen condensation cyclization. The TE/CLC domains of NR-PKSs in group II biosynthesizing 1,3,6,8-tetrahydroxynaphthalene analogs diverge into two different catalytic mechanisms. Some TE/CLC domains merely accelerate cyclization to release substrates, such as in *Exophiala lecanii-corni* PKS1 (Cheng et al., 2004), and the others with dual-function catalyze cyclization and deacetylation, as in *Colletotrichum lagenaria* PKS1 (Vagstad et al., 2012b). The TE/CLC domains of NR-PKSs in group III cyclize the second ring formations of heptaketides and nonaketides, such as in *A. nidulans* WA (Fujii et al., 2001) and *Fusarium fujikuroi* PKS4 (Linnemannstons et al., 2002). The TE domains of C–C bond formations, belonging to the NR-PKSs in group IV, cyclize the third ring formation of decaketides via C14–C1 Claisen condensation cyclization, such as in *A. parasiticus* PksA (Korman et al., 2010).

The vast majority of R domains locate at the C-terminus of NR-PKSs in groups IV and VII (**Figure 3**). The R domains reductively release the different polyketide chains through catalyzing an NAD(P)H-dependent two-electron reduction of the acyl thioester to an aldehyde intermediate. The R domain of *Acremonium strictum* MOS producing xenovulene is the first identified PRE domain with reductive release mechanism during polyketide biosynthesis (Bailey et al., 2007). Rarely appearing in the NR-PKSs, the only known physically discrete R domain is employed by *Aspergillus niger* DtbA of NR-PKSs in group VI (Yeh et al., 2013). Additionally, although NR-PKSs in groups VI and VII were close relative characterized by CMeT domains, the PRE domains of NR-PKSs in group VI were TE-like and physically discrete R domains, while in group VII were C-terminal R domains.

Interestingly, in NR-PKSs of group V, the physically discrete MβL-TE domains are employed. Different from C-terminal PRE domains in fungal NR-PKS family, the MβL-TE domains evolve independently and do not derive from ancient orsellinic acid synthases. The emodin octaketide synthase ACAS from *A. terreus* releases polyketide chains by ACTE which is the first example of discrete MβL-TE domains (Awakawa et al., 2009). The vast majority of MβL-TE domains catalyze the reactions of fourth-ring cylization or hydrolysis to release the various polyketide intermediates. It is necessary for cylization reaction to form α-hydroxylated polyketide by a flavin adenine dinucleotide (FAD)-dependent monooxygenase (FMO) (Li et al., 2011). Most MβL-TE domains catalyze the reactions with binding two Zn^2+^ cations, while a few members exhibit preferential requirements for two Mn^2+^ cations, such as *A. nidulans* AptB (Li et al., 2011).

### Structure and Sequence Analyses of PRE Domains

**FIGURE 4:**
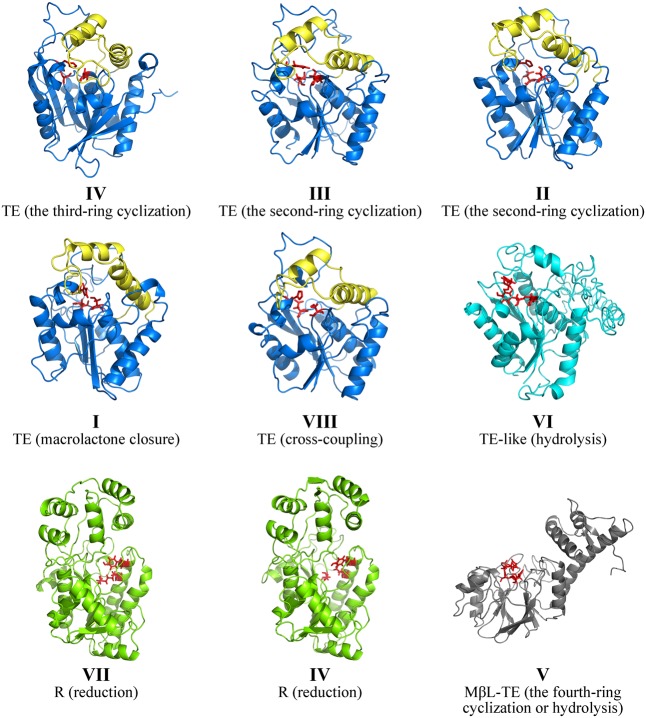
Comparison of PRE structures of NR-PKSs. The TE structure of NR-PKS in group IV is displayed by 3ILS PDB crystal structure and the PRE domains of NR-PKSs in other groups are showed by model structures. The TE structures of NR-PKSs in groups I–III and VIII are exemplified by XP_681178 from group I, BAA18956 from group II, Q03149 from group III, and AFL91703 from group VIII. The TE-like structure is exemplified by XP_681652 from group VI. The R structures are exemplified by XP_658638 from group VII and XP_003039929 from group IV. The MβL-TE structure is exemplified by CBF70385 from group V. The side chain atoms of catalytic sites in PRE domains are highlighted with red color. The lid regions in TE structures are highlighted with yellow color.

In order to illustrate the PRE structures corresponding to various product release mechanisms, the three-dimensional structures of all PRE domains from the selected 58 NR-PKSs with known functions were modeled and analyzed. The three-dimensional PRE structures of NR-PKSs in groups I–VIII were built by comparative protein modeling method. According to quality assessment of Ramachandran plot, the accuracy of all the PRE protein model structures was acceptable (Supplementary Table S3). Based on the above simulated structures, the structure and sequence analyses of PRE domains showed obvious differences between TE, R, and MβL-TE. In the whole, the TE and TE-like proteins show the α/β-hydrolase folds with different lid structures, while the R and MβL-TE proteins display α/β Rossmann fold and typical MβL fold, respectively (**Figure 4**). In addition, the catalytic sites of PRE domains in three types are also distinguishing significantly.

The TE domains have been considered to be the most widely distributed PRE domains in NR-PKSs and perform the most various product release mechanisms (Du and Lou, 2010; Korman et al., 2010). Up to date, only one crystal structure of PRE domain in fungal NR-PKSs has been reported from the TE/CLC domain of *A. parasiticus* PksA (Korman et al., 2010). The TE/CLC domain of PksA catalyzes the third ring closure reaction via C14–C1 register. The TE/CLC structure of PksA displays an α/β-hydrolase fold in the catalytic closed form and a conserved catalytic triad (Ser1937, Asp1964, and His2088) in the α/β-hydrolase family. The TE/CLC protein of PksA habors a deep, hydrophobic substrate-binding chamber formed between the α/β-hydrolase core and the lid region inserted between β6 and β7, which is distinct from the TE structures from bacterial modular PKSs (Tsai et al., 2001, 2002; Giraldes et al., 2006), non-ribosomal peptide synthetases (Bruner et al., 2002; Samel et al., 2006), and human fatty acid synthase (Chakravarty et al., 2004; Pemble et al., 2007). It was proposed that the TE/CLC protein of PksA adopts two conformations with open and closed lid.

Our analysis of model structures indicated that the TE proteins of NR-PKSs in groups I–IV and VIII display an α/β-hydrolase fold and a loop between two helices of lid, similar to that of PksA (**Figure 4**). The residues of catalytic triad (Ser-Asp-His) are highly conserved in diverse TE domains, as previous mutation experiments reported (Fujii et al., 2001; Wang et al., 2009; Korman et al., 2010; Zhou et al., 2010). However, the TE domains in different NR-PKS groups perform different catalytic mechanisms, such as chain length selection and bond formation of polyketide intermediates (**Figure 3B** and **Supplementary Figure S1**).

**FIGURE 5:**
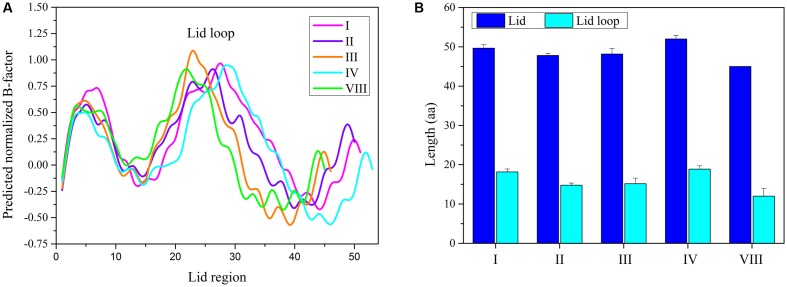
The TE-lid analyses of NR-PKSs in groups I–IV and VIII. **(A)** B factor analyses for each residue site in TE domains of NR-PKSs in groups I–IV and VIII. The B factors for TE-lid regions of NR-PKSs in groups I–IV and VIII are displayed by the same representative sequences as in **Figure 4**. **(B)** Sequence length analyses of lids and loops in TE domains of NR-PKSs in groups I–IV and VIII. Error bars represent standard error of the mean (SEM).

In order to study the different TE domains corresponding to various release mechanisms, the structure and sequence differentiations of TE domains of NR-PKSs in groups I–IV and VIII were analyzed and compared. The structural differentiation analyses were focused on the lid regions and catalytic pockets of TE domains. The results indicated that all TE domains of NR-PKSs in groups I–IV and VIII possess a similar loop region between two lid helices. B factor is a measure to capture the atomic vibrational motion. Analysis of the B factor for lid region in each TE structure showed that there are three regions of high disorder, corresponding with loop and two ends of lid (**Figure 5A**). The two flexible ends of lid suggested that the integral lid region could open and close within a certain space. The highest flexible loop in the middle of lid may be the entrance for substrate to move into the chamber.

**FIGURE 6:**
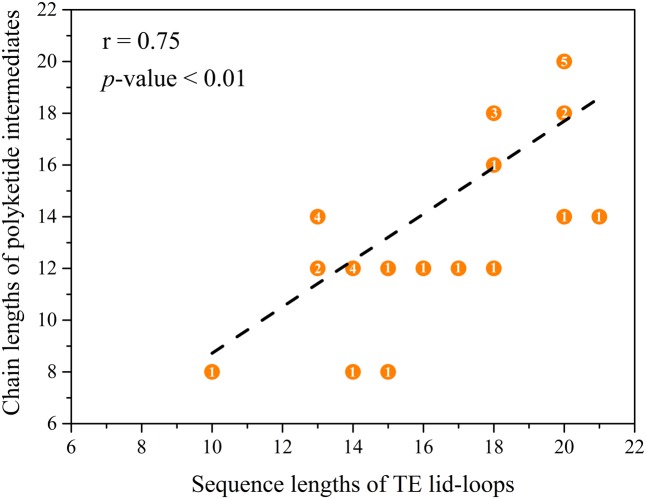
Correlations between the lid-loop sequence lengths and chain lengths of polyketide intermediates of TE domains of NR-PKSs in groups I–IV and VIII. The TE domains with known functions were used to be analyzed. The numbers of carbon atoms were used to represent the chain length of polyketide intermediates which were acquired from the literature. The overlapping points and the number of overlapping times have been indicated.

By comparing the lid and loop sequence lengths of TE domains of NR-PKSs in groups I–IV and VIII, it could be found that the lid sequence length differences mainly ascribe to the loop sequence length differences (**Figure 5B**). It should be noted that there is an interesting relationship between loop sequence lengths and synthesized compound sizes. The average sequence length of TE lid-loops of NR-PKSs in group IV is the longest (∼19 residues) corresponding with the products of decaketides, while shortest (∼12 residues) in group VIII with tetraketides. It is probable that the loop regions of TE domains could play a crucial role in substrate recognition. To better understand their relationship, the statistical data has been calculated (Supplementary Table S4). The statistical data of lid-loop sequence lengths and chain lengths of polyketide intermediates of TE domains in NR-PKSs (groups I–IV and VIII) showed a high degree of positive correlation (**Figure 6**, *r* = 0.75, *p*-value < 0.01).

**FIGURE 7:**
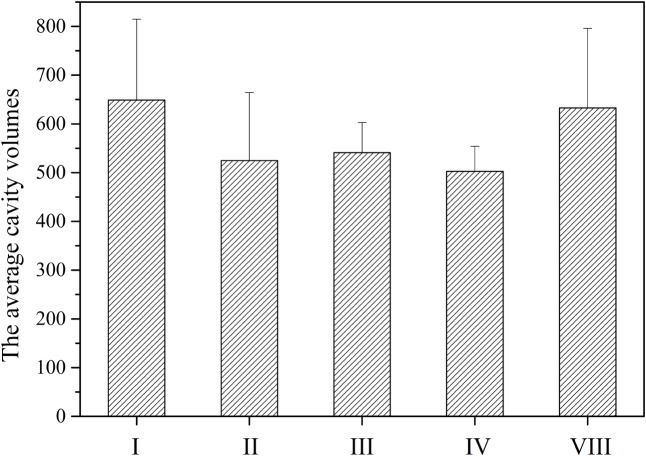
The average cavity volumes of TE catalytic pockets of NR-PKSs in groups I–IV and VIII. The cavity volumes were calculated and identified by CASTp and CAVER. The cavity volume of TE protein in each NR-PKS group was defined as the average value of cavity volumes of all TE model structures in the same group of NR-PKSs. Error bars represent the standard deviation.

The cavity volumes of TE catalytic pockets were calculated and analyzed by CASTp and CAVER. The average cavity volumes of TE catalytic pockets of NR-PKSs in groups I–IV and VIII are about 500–650 Å^3^. It was found that the average cavity volume of TE catalytic pockets with macrolactone closure in group I is the biggest one, followed by that of intermolecular cross-coupling in group VIII (**Figure 7**). The average cavity volumes of O–C bond-forming pockets in groups I and VIII are significantly bigger than that of C–C bond-forming pockets in groups II–IV. However, the cavity volumes of TE catalytic pockets with C–C ring closure reactions are similar despite the different chain lengths of polyketide intermediates synthesized by NR-PKSs in groups II–IV.

**FIGURE 8:**
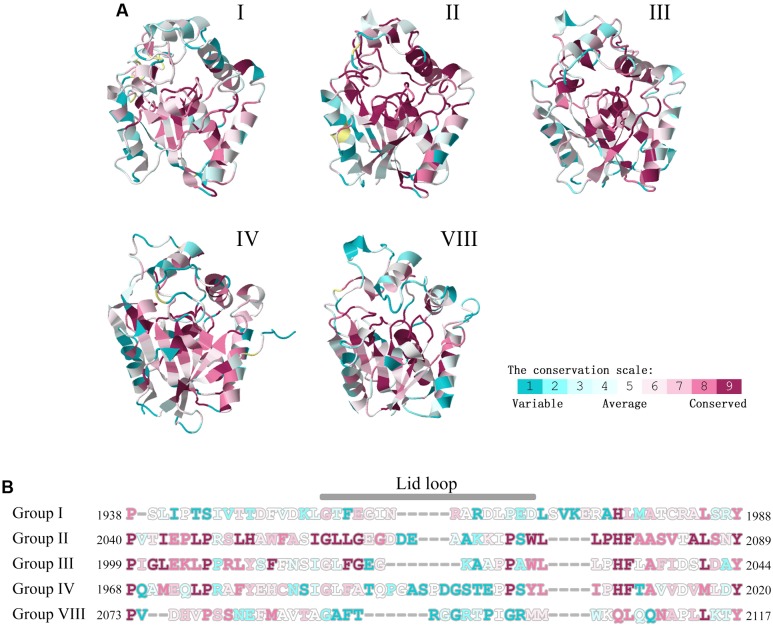
Conservation scale analyses of residue sites in TE domains of NR-PKSs in groups I–IV and VIII. **(A)** The TE structure of NR-PKS in group IV is displayed by 3ILS PDB crystal structure, while other TE domains are showed by model structures. The TE structures of NR-PKSs in groups I–IV and VIII are exemplified by the same representative sequences as in **Figure 4**. Conservation scale is defined from the most variable residue sites (grade 1, color represented by turquoise) which are considered as rapidly evolving to conservative residue sites (grade 9, color represented by maroon) which are considered as slowly evolving. **(B)** Conservation analyses of residue sites in lid regions. The numbers of residue sites in TE-lid regions of NR-PKSs in groups I–IV and VIII are displayed by the same representative sequences as in **Figure 4**.

Due to the structures are confined by sequences, the TE sequence differentiations of NR-PKSs in groups I–IV and VIII were also analyzed. The conservation analyses of all amino acid residue sites were performed with the evolutionary conservation scores calculated by ConSurf. The results showed that the majority of conserved residue sites (ConSurf grade 7–9) in TE sequences of NR-PKSs in groups I–IV and VIII locate at the catalytic pockets (**Figure 8A** and Supplementary Table S5). Therefore, based on the TE crystal structure of PksA, 27 CLR sites of TE domains were predicted by using CASTp and CAVER (Supplementary Table S5). Among them, 11 CLR sites locate at the lid regions of TE domains. It was found that there are nine most conserved CLR sites showing the same or similar residues in the predicted CLR sites of TE domains in NR-PKSs of groups I–IV and VIII. These CLR sites in the catalytic pockets present as Asp1873, Gly1874, Trp1936, Phe2056, Phe2089, and Ser1937-Asp1964-His2088 (catalytic sites), while in the lid regions only Pro1968 occurs (amino acid numbering as in Q12053). It should be noted that the results revealed that the small side chain residues Gly and Leu in the conserved CLRs in the TE catalytic pockets in group I were replaced by the conserved CLRs Ser1938 and Phe2010 (amino acid numbering as in Q12053) in groups II–IV, respectively, resulting in the decrease of pocket volumes. The positively charged residue Arg in group I was substituted by the conserved Pro1966 in groups II–IV. The conserved Leu1974 identical in TE lid regions of groups II–IV are not conserved in groups I and VIII. In a previous study of TE crystal structure of PksA, it was proposed that Trp1936 and Phe2010 together with other two residues envelope the hexyl portion of the substrate to cause the conformational change of the hexyl group, and backbone nitrogens of Gly1874 and Ser1938 act as the oxyanion hole to stabilize the transacylation reaction (Korman et al., 2010). In addition, the conservation analyses of TE lid regions showed that the loop regions are mainly composed by various residue sites (**Figure 8B**, ConSurf grade 1–3). The conservation of residue sites in the loop is obviously lower than those of helixes at two sides. Specifically, the Weblogo was generated to visualize the sequence diversity at each position of TE lid regions (**Supplementary Figure S2**). It was found that the highly conserved aromatic amino acids and charged amino acids of TE lid regions may be potential functional sites.

The structural and functional differentiations of TE-like, R, and MβL-TE domains are relatively simple compared to TE domains according to our analyses. The structure types of TE-like, R, and MβL-TE domains were obviously different from the TE domains. The TE-like structures of NR-PKSs in group VI are different from TE structures of NR-PKSs in groups I–IV and VIII in the lid regions. The lid regions of TE-like domains could not be finely modeled, despite the α/β-hydrolase core of TE-like domains are similar to those of TE domains (**Figure 4**). Multiple sequence alignment revealed that the average lid length of TE-like domains is more than 67 amino acid residues, comparing with those of TE domains (45–52 residues) of NR-PKSs in groups I–IV and VIII. In addition, it is worth noting that the residue Asp of catalytic triad is not conserved in TE-like domains. According to multiple sequence alignment, it could be deduced that the catalytic triad residues in TE-like domains should be Ser2251, Tyr2279, and His2445 (amino acid numbering as in XP_681652).

To date, neither R nor MβL-TE crystal structure in fungal NR-PKSs has been reported. The results of model structures revealed that both R structures of NR-PKSs in groups IV and group VII are similar to that of non-ribosomal peptide synthetase module MxaA in *Stigmatella aurantiaca* Sga15 (Barajas et al., 2015). All of the R structures of NR-PKSs contain an N-terminal NADPH-binding subdomain and a C-terminal substrate-binding subdomain, belonging to type E short-chain dehydrogenases/reductases family. The N-terminal region of R structure is highly conserved and contains an extended NADPH-binding α/β Rossmann fold with seven parallel β sheets flanked by five α helices. The R domain of NR-PKS features a catalytic triad Ser-Tyr-Lys (as Ser2045, Tyr2080, and Lys2084 of XP_003039929 in group IV; Ser2525, Tyr2553, and Lys2557 of XP_658638 in group VII), which is the same as in R domain of peptaibol synthetase, the relative of NR-PKSs, from *Trichoderma virens* (Manavalan et al., 2010).

The model structures of MβL-TE proteins employed by NR-PKSs of group V display the typical MβL fold at the N-terminal, with a central double β sheet sandwich flanked by helices and loops (**Figure 4**). The residues His97, His99, Asp101, His102, His153, Asp172, and His207 of MβL-TE domains (amino acid numbering as in CBF70385) are critical for binding substrates and catalyzing hydrolytic activities reported by the previous biochemical experiment with MβL-TE domain of *A. nidulans* AptB (Li et al., 2011). It could be proposed that the seven residues should locate at the catalytic pocket and be related to bind two Zn^2+^ or Mn^2+^ cations.

The above analyses concerning PRE structures and sequences indicated that the differentiations of the structural characteristics and catalytic sites result in the PRE functional differentiations. Specifically, for TE domains in NR-PKSs of groups I–IV and VIII, the structure and sequence variations of lid-loops and catalytic pockets contribute to the functional differentiations. The TE-like domains of NR-PKSs in group VI are distantly diverged from TE domains with different lid regions and catalytic sites.

## Discussion

In the past decade, more research on PRE domains in fungal NR-PKSs indicated that PRE domains develop into various and complicated catalytic mechanisms. The differentiation of catalytic mechanisms of diverse TE domains between the NR-PKSs in groups I–IV and VIII mainly involve chain length selection and bond formation of polyketide intermediates. Our study revealed that the sequence lengths of lid-loops and chain lengths of polyketide intermediates of TE domains showed a positive correlation. This finding is consistent with the previous studies, including the swapping experiments of TE domains and lid regions, respectively (Vagstad et al., 2012b; Newman et al., 2014). The TE domain of *A. nidulans* WA, with loop sequence length of 13 residues, was found to catalyze the release of hexaketide and heptaketide intermediates efficiently, while to be inefficient off-loading with the nonaketide and decaketide intermediates. Similarly, the TE domain of *C. nicotianae* CTB1, with loop sequence length of 20 residues, was validated to release heptaketide and nonaketide intermediates in a high level of production, however, catalyze hexaketide intermediates in a slow off-loading rate. The TE domain of *A. parasiticus* PksA, with loop sequence length of 20 residues, was verified to release the decaketide intermediates in high yields, while catalyze the release of hexaketide and nonaketide intermediates at a low level. The TE domains displayed a degree of substrate promiscuity and could accept chain lengths of polyketide intermediates with plus or minus one extension. Moreover, the similar situations exist in the bromoperoxidase A2 (BPO-A2) and surfactin synthase TE (Koglin et al., 2008; Chen et al., 2009), which also belong to the α/β-hydrolase family as TE. A previous study reported that by deleting the lid region of BPO-A2, the variant showed higher hydrolytic activities toward a substrate with a long chain, while its activity decreased dramatically toward a substrate with a short chain (Chen et al., 2009). Another literature proposed that by NMR titration experiments of surfactin-synthetase TE, the sequentially diverse lid and its loop present to be important for selecting the specific substrates (Koglin et al., 2008). It should be noted that the lid region whose folding process was independent to the core α/β-hydrolase could be operated with insertion, deletion, and substitution (Chen et al., 2009; Vagstad et al., 2012b). Therefore, it is essential to characterize the lid-loops of TE domains for operate genetic manipulation to obtain more new metabolites targetedly.

The results by our analysis also indicated that the cavity volumes of TE catalytic pockets with C–C ring closure reactions are similar although the chain lengths of polyketide intermediates synthesized by NR-PKSs of groups IV is obviously longer than others synthesized by NR-PKSs of groups II and III. It should be reasonable to deduce that the TE catalytic pockets of NR-PKSs in group IV develop a special protein conformation to position hexyl portion of the substrate. A previous study indicated that the hexyl portion of decaketide was enveloped by residues Gly1875, Trp1936, Gln1991, and Phe2010 (Korman et al., 2010). The conformational change of the hexyl group locks C-14 close to catalytic His2088 and controls substrate positioning for enolate formation and subsequent C–C cyclization. The TE domain of *A. parasiticus* PksA catalyzes the intermediates with a unique hexanoyl starter unit, while the TE domains of *C. lagenaria* Pks1 and *F. fujikuroi* Pks4 cyclize acetyl-initiated polyketides. In the swapping experiments, when the PksA TE domain of *A. parasiticus* replaced the TE domains of *C. lagenaria* Pks1 and *F. fujikuroi* Pks4, respectively, the PksA TE domain were found almost impossibly to catalyze the acetyl-initiated intermediates (Newman et al., 2014). Similarly, when the Pks1 TE and Pks4 TE replaced the PksA TE, respectively, it was also found that Pks1 TE and Pks4 TE could not catalyze the cyclizations of hexanoyl-initiated intermediates synthesized by *A. parasiticus* PksA (Newman et al., 2014). Considering that the mature products released by the TE domains, the cavity volumes of catalytic pockets may correlate with the chemical structures of the released products. However, based on the reported data, the precise correlation between the cavity volumes and chemical structures of the released products of TE domains could not be obtained.

Besides the diverse TE domains, our interesting findings also involve TE-like and R domains. The catalytic sites of TE-like domains display the intermediate formation between TE and R domains, as Ser-Asp-His in TE domains, Ser-Tyr-Lys in R domains, while Ser-Tyr-His in TE-like domains. Based on the phylogenetic tree of NR-PKSs and PRE domains, the evolutionary status of TE-like domains also exist between TE and R domains (**Figures 2A**, **3A**). The relationship of catalytic sites between TE, R, and TE-like domains is similar to that of phylogenetic tree of NR-PKSs and PRE domains. Considering distantly phylogenetic relationships of NR-PKSs between groups IV and VII, we deduced that the R domains of NR-PKSs in group IV should derive from separate gene fusion events and domain replacements. It could be further implied that the PRE domains at the C-terminus of NR-PKSs should originate from separate gene fusion events.

In the past decades, the PRE domains have been reported as TE, R, and MβL-TE domains in literature (Chooi and Tang, 2012). In our study, the TE-like domains were separated from TE domains according to their sequences, structures, and functions. In the previous studies, the functions of PRE domains were labeled on the phylogenetic tree of NR-PKSs involved groups I–VII, and these reports were individual, incomplete, and unsystematic (Vagstad et al., 2012b; Yeh et al., 2013). Additionally, the functions of TE domains were presumed to differentiate into diverse catalytic mechanisms based on the phylogeny of TE domains (Lackner et al., 2013). In our study, the PRE domains were summarized and compared systematically with more complete data. The differentiations of PRE domains were labeled in the phylogenetic tree of 574 NR-PKSs involved in all of groups I–VIII. The complex diverse functions of PRE domains were analyzed and compared based on the phylogeny of PRE domains concerning not only TE domain, but also TE-like, R, and MβL-TE domains. Based on our analysis, it was found that the functional differentiation of TE domains is attributed to the changes of the sequence lengths of lid-loops and cavity volumes of catalytic pockets arised from the sequence variations. Particularly, the sequence lengths of lid-loops and chain lengths of polyketide intermediates catalyzed by TE domains showed a positive correlation. This finding is reported for the first time.

The above results by our study may help to predict the structures of fungal secondary metabolites by genome mining. Based on the phylogeny of PRE domains of fungal NR-PKSs, the structures of secondary metabolites could be estimated primarily. The cavity volumes of TE catalytic pockets could help us to understand the bond formation types of polyketide intermediates. According to the sequence lengths of TE lid-loops, the possible chain lengths of polyketide intermediates of TE domains could be recognized.

Until now, the chain-release mechanisms of several NR-PKSs in group V are still mysterious, which may exceed the known mechanisms. These issues indicated that besides C-terminal PRE domains, NR-PKSs may recruit MβL-TE and other possible types of domains, which demonstrate the versatility, flexibility and complexity of PRE domains. The unknown product release mechanisms should be revealed in the future and may lead to new insights.

## Conclusion

In the present study, the relationships between sequences, structures, and functions of PRE domains in fungal NR-PKSs were systematically analyzed and elucidated. Our investigation revealed that the PRE domains could be mainly classified into three types, TE, R, and MβL-TE, with distinct catalytic modes caused by different structural characteristics as well as catalytic sites. The evolution routes of TE domains display complicated functional divergences. Furthermore, the functional differentiations of various TE domains are attributed to the structural changes, such as the lid-loop sequence lengths and catalytic cavity volumes, arising from the sequence variations. The specific catalytic sites and structural characteristics of TE-like and R domains were described for the first time. The above findings may provide the novel mechanistic insights for PRE domains and may help to predict the structural characteristics of the aromatic polyketides biosynthesized by fungal NR-PKSs. It could be prospected to regulate the biosynthetic pathways and further apply the combinatorial biosynthesis to obtain new target products.

## Author Contributions

LL and ZZ designed the experiments, analyzed the data, and wrote the paper; C-LS and C-YW directed the experiments, and wrote and revised the manuscript. All authors gave final approval for publication.

## Conflict of Interest Statement

The authors declare that the research was conducted in the absence of any commercial or financial relationships that could be construed as a potential conflict of interest.

## Supplementary Material

**FIGURE S1 |** The schematic diagram of representative product-releasing modes catalyzing the substrate moieties to form the mature polyketide products of PRE domains in NR-PKSs of all eight groups.

**FIGURE S2 |** The sequence diversity at each position of TE lid regions.
