# Supplementary figures and images for: Analysis of the Sequences, Structures, and Functions of Product-Releasing Enzyme Domains in Fungal Polyketide Synthases
Source: Front Microbiol. 2017 Sep 4;8:1685. doi: 10.3389/fmicb.2017.01685 (PMC5591372; doi:10.3389/fmicb.2017.01685)

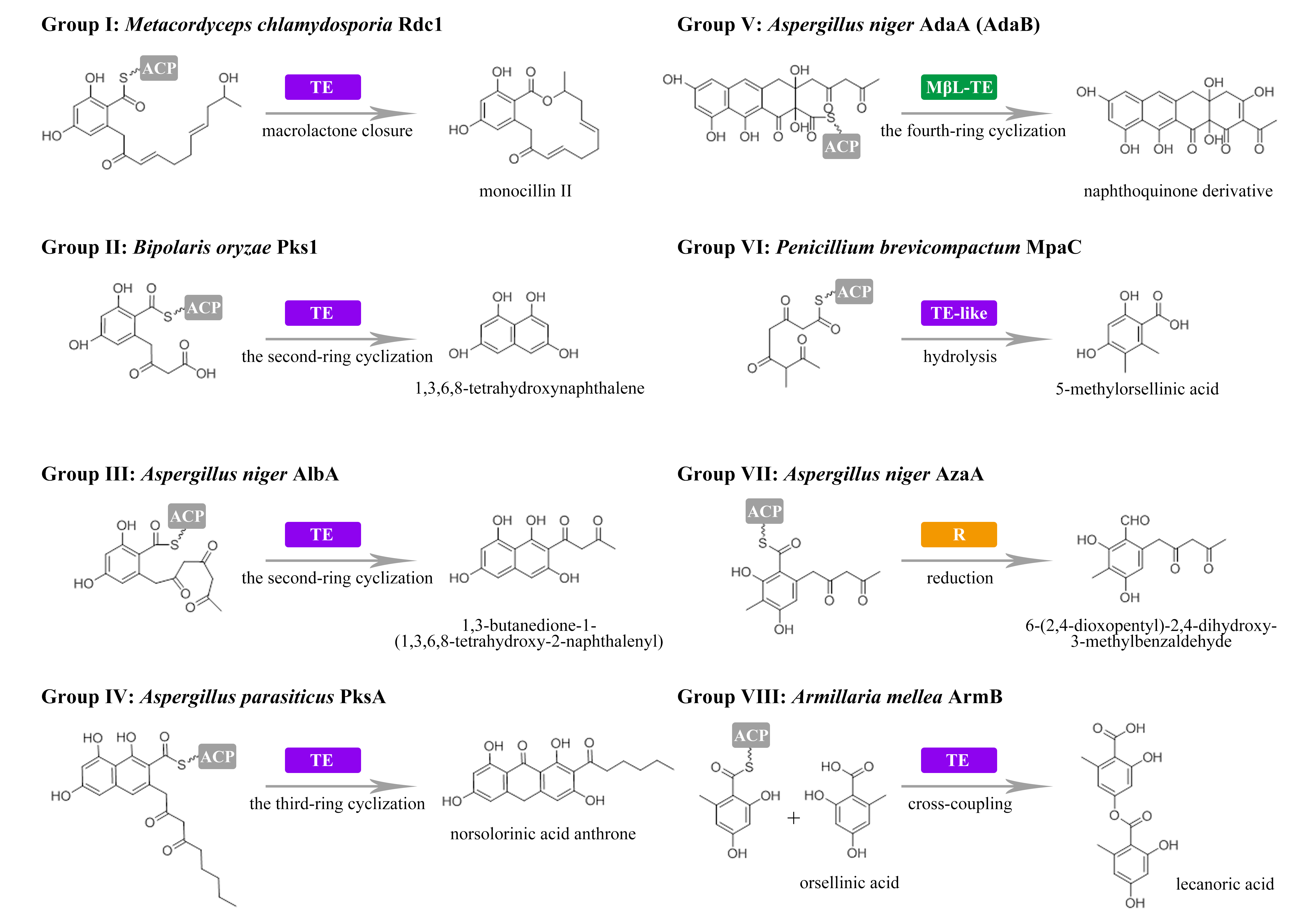

Supplement: Supplementary file 1 [file Image_1.TIF]

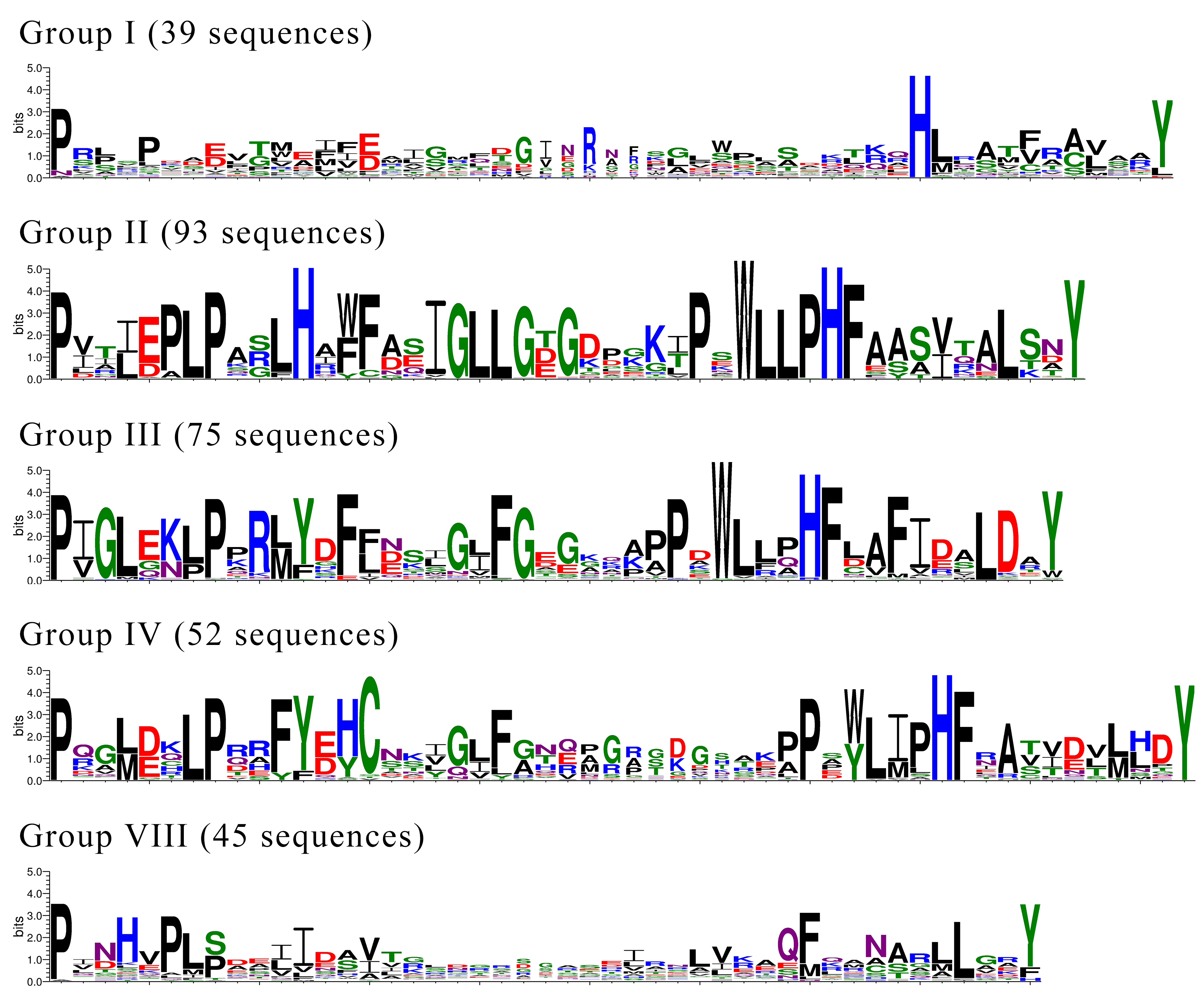

Supplement: Supplementary file 2 [file Image_2.TIF]
