# Supplementary material for: Analysis of the Sequences, Structures, and Functions of Product-Releasing Enzyme Domains in Fungal Polyketide Synthases
Source: Front Microbiol. 2017 Sep 4;8:1685. doi: 10.3389/fmicb.2017.01685 (PMC5591372; doi:10.3389/fmicb.2017.01685)
Supplement: Supplementary file 3 [file Table_1.DOCX]

# Table S1. List of 574 NR-PKSs introduced in the phylogenic analysis.

| **S/N** | **Group** | **Accession No.** | **PRE** | **Taxon** | **Strain Name** |
| --- | --- | --- | --- | --- | --- |
| 1 | VII | CAN87161 | R | ascomycetes | *Acremonium strictum* |
| 2 | II | AEH76763 | TE | ascomycetes | *Alternaria alternata* |
| 3 | II | AFN68292 | TE | ascomycetes | *Alternaria alternata* |
| 4 | II | BAK64048 | TE | ascomycetes | *Alternaria alternata* |
| 5 | VIII | AFL91703 | TE | basidiomycetes | *Armillaria mellea* |
| 6 | IV | XP_003013882 | TE | ascomycetes | *Arthroderma benhamiae CBS 112371* |
| 7 | V | XP_003013353 | none | ascomycetes | *Arthroderma benhamiae CBS 112371* |
| 8 | VII | XP_003010590 | R | ascomycetes | *Arthroderma benhamiae CBS 112371* |
| 9 | IV | XP_003169137 | TE | ascomycetes | *Arthroderma gypseum CBS 118893* |
| 10 | V | XP_003170602 | none | ascomycetes | *Arthroderma gypseum CBS 118893* |
| 11 | VII | XP_003170502 | R | ascomycetes | *Arthroderma gypseum CBS 118893* |
| 12 | III | XP_002847317 | TE | ascomycetes | *Arthroderma otae CBS 113480* |
| 13 | IV | XP_002842704 | TE | ascomycetes | *Arthroderma otae CBS 113480* |
| 14 | V | XP_002846705 | none | ascomycetes | *Arthroderma otae CBS 113480* |
| 15 | V | XP_002848092 | none | ascomycetes | *Arthroderma otae CBS 113480* |
| 16 | VI | XP_002843683 | TE-like | ascomycetes | *Arthroderma otae CBS 113480* |
| 17 | VII | XP_002846669 | R | ascomycetes | *Arthroderma otae CBS 113480* |
| 18 | VII | XP_002848394 | R | ascomycetes | *Arthroderma otae CBS 113480* |
| 19 | III | BAJ65431 | TE | ascomycetes | *Aspergillus aculeatus* |
| 20 | III | XP_001276035 | TE | ascomycetes | *Aspergillus clavatus NRRL 1* |
| 21 | V | XP_001275038 | none | ascomycetes | *Aspergillus clavatus NRRL 1* |
| 22 | VII | XP_001273475 | R | ascomycetes | *Aspergillus clavatus NRRL 1* |
| 23 | IV | AAS90093 | TE | ascomycetes | *Aspergillus flavus* |
| 24 | III | XP_002382817 | TE | ascomycetes | *Aspergillus flavus NRRL3357* |
| 25 | III | XP_002384329 | TE | ascomycetes | *Aspergillus flavus NRRL3357* |
| 26 | IV | XP_002373130 | TE | ascomycetes | *Aspergillus flavus NRRL3357* |
| 27 | IV | XP_002379951 | TE | ascomycetes | *Aspergillus flavus NRRL3357* |
| 28 | V | XP_002376725 | none | ascomycetes | *Aspergillus flavus NRRL3357* |
| 29 | V | XP_002378746 | none | ascomycetes | *Aspergillus flavus NRRL3357* |
| 30 | VII | XP_002379030 | R | ascomycetes | *Aspergillus flavus NRRL3357* |
| 31 | VII | XP_002381902 | R | ascomycetes | *Aspergillus flavus NRRL3357* |
| 32 | VII | XP_002384396 | R | ascomycetes | *Aspergillus flavus NRRL3357* |
| 33 | VIII | XP_002377153 | TE | ascomycetes | *Aspergillus flavus NRRL3357* |
| 34 | III | AAC39471 | TE | ascomycetes | *Aspergillus fumigatus* |
| 35 | III | EDP55264 | TE | ascomycetes | *Aspergillus fumigatus A1163* |
| 36 | V | EDP47078 | none | ascomycetes | *Aspergillus fumigatus A1163* |
| 37 | V | EDP47964 | none | ascomycetes | *Aspergillus fumigatus A1163* |
| 38 | V | EDP50840 | none | ascomycetes | *Aspergillus fumigatus A1163* |
| 39 | VIII | EDP49937 | TE | ascomycetes | *Aspergillus fumigatus A1163* |
| 40 | III | XP_756095 | TE | ascomycetes | *Aspergillus fumigatus Af293* |
| 41 | V | XP_746435 | none | ascomycetes | *Aspergillus fumigatus Af293* |
| 42 | V | XP_746913 | none | ascomycetes | *Aspergillus fumigatus Af293* |
| 43 | V | XP_751377 | none | ascomycetes | *Aspergillus fumigatus Af293* |
| 44 | VII | XP_748578 | R | ascomycetes | *Aspergillus fumigatus Af293* |
| 45 | VI | GAA90162 | TE-like | ascomycetes | *Aspergillus kawachii IFO 4308* |
| 46 | III | GAA88246 | TE | ascomycetes | *Aspergillus kawachii IFO 4308* |
| 47 | III | GAA90949 | TE | ascomycetes | *Aspergillus kawachii IFO 4308* |
| 48 | V | GAA85937 | none | ascomycetes | *Aspergillus kawachii IFO 4308* |
| 49 | V | GAA88581 | none | ascomycetes | *Aspergillus kawachii IFO 4308* |
| 50 | VII | GAA89817 | R | ascomycetes | *Aspergillus kawachii IFO 4308* |
| 51 | VII | GAA92425 | R | ascomycetes | *Aspergillus kawachii IFO 4308* |
| 52 | III | BAK53402 | TE | ascomycetes | *Aspergillus luchuensis* |
| 53 | IV | AAA81586 | TE | ascomycetes | *Aspergillus nidulans* |
| 54 | VI | XP_664052 | TE-like | ascomycetes | *Aspergillus nidulans FGSC A4* |
| 55 | VII | ANID_07903 | R | ascomycetes | *Aspergillus nidulans FGSC A4* |
| 56 | VII | XP_659636 | R | ascomycetes | *Aspergillus nidulans FGSC A4* |
| 57 | I | XP_681178 | TE | ascomycetes | *Aspergillus nidulans FGSC A4* |
| 58 | III | Q03149 | TE | ascomycetes | *Aspergillus nidulans FGSC A4* |
| 59 | IV | Q12397 | TE | ascomycetes | *Aspergillus nidulans FGSC A4* |
| 60 | V | XP_657754 | none | ascomycetes | *Aspergillus nidulans FGSC A4* |
| 61 | V | XP_663604 | none | ascomycetes | *Aspergillus nidulans FGSC A4* |
| 62 | V | XP_664675 | none | ascomycetes | *Aspergillus nidulans FGSC A4* |
| 63 | VI | XP_681652 | TE-like | ascomycetes | *Aspergillus nidulans FGSC A4* |
| 64 | VII | XP_658127 | R | ascomycetes | *Aspergillus nidulans FGSC A4* |
| 65 | VII | XP_658638 | R | ascomycetes | *Aspergillus nidulans FGSC A4* |
| 66 | VII | XP_660834 | R | ascomycetes | *Aspergillus nidulans FGSC A4* |
| 67 | VII | XP_660990 | R | ascomycetes | *Aspergillus nidulans FGSC A4* |
| 68 | VII | est_GWPlus_C_190476 | R | ascomycetes | *Aspergillus niger* |
| 69 | III | EHA21301 | TE | ascomycetes | *Aspergillus niger ATCC 1015* |
| 70 | III | EHA28527 | TE | ascomycetes | *Aspergillus niger ATCC 1015* |
| 71 | V | EHA20150 | none | ascomycetes | *Aspergillus niger ATCC 1015* |
| 72 | VII | EHA25844 | R | ascomycetes | *Aspergillus niger ATCC 1015* |
| 73 | VII | EHA28237 | R | ascomycetes | *Aspergillus niger ATCC 1015* |
| 74 | III | XP_001390425 | TE | ascomycetes | *Aspergillus niger CBS 513.88* |
| 75 | III | XP_001393884 | TE | ascomycetes | *Aspergillus niger CBS 513.88* |
| 76 | V | XP_001394705 | none | ascomycetes | *Aspergillus niger CBS 513.88* |
| 77 | V | XP_001402309 | none | ascomycetes | *Aspergillus niger CBS 513.88* |
| 78 | VI | XP_001393524 | TE-like | ascomycetes | *Aspergillus niger CBS 513.88* |
| 79 | VII | XP_001390084 | R | ascomycetes | *Aspergillus niger CBS 513.88* |
| 80 | VII | XP_001393501 | R | ascomycetes | *Aspergillus niger CBS 513.88* |
| 81 | VII | XP_001395291 | R | ascomycetes | *Aspergillus niger CBS 513.88* |
| 82 | IV | AAS90047 | TE | ascomycetes | *Aspergillus nomius* |
| 83 | IV | ACH72912 | TE | ascomycetes | *Aspergillus ochraceoroseus* |
| 84 | IV | BAE71314 | TE | ascomycetes | *Aspergillus oryzae* |
| 85 | VII | KDE82080 | R | ascomycetes | *Aspergillus oryzae 100-8* |
| 86 | III | EIT79828 | TE | ascomycetes | *Aspergillus oryzae 3.042* |
| 87 | IV | EIT81356 | TE | ascomycetes | *Aspergillus oryzae 3.042* |
| 88 | IV | EIT81872 | TE | ascomycetes | *Aspergillus oryzae 3.042* |
| 89 | V | EIT75060 | none | ascomycetes | *Aspergillus oryzae 3.042* |
| 90 | V | EIT80443 | none | ascomycetes | *Aspergillus oryzae 3.042* |
| 91 | VI | EIT79328 | TE-like | ascomycetes | *Aspergillus oryzae 3.042* |
| 92 | VII | EIT73076 | R | ascomycetes | *Aspergillus oryzae 3.042* |
| 93 | VII | EIT81451 | R | ascomycetes | *Aspergillus oryzae 3.042* |
| 94 | VII | EIT82410 | R | ascomycetes | *Aspergillus oryzae 3.042* |
| 95 | VIII | EIT78482 | TE | ascomycetes | *Aspergillus oryzae 3.042* |
| 96 | III | XP_001822700 | TE | ascomycetes | *Aspergillus oryzae RIB40* |
| 97 | IV | XP_001817959 | TE | ascomycetes | *Aspergillus oryzae RIB40* |
| 98 | IV | XP_001821511 | TE | ascomycetes | *Aspergillus oryzae RIB40* |
| 99 | V | BAE58990 | none | ascomycetes | *Aspergillus oryzae RIB40* |
| 100 | V | XP_001823362 | none | ascomycetes | *Aspergillus oryzae RIB40* |
| 101 | VI | XP_001816639 | TE-like | ascomycetes | *Aspergillus oryzae RIB40* |
| 102 | VII | BAE56924 | R | ascomycetes | *Aspergillus oryzae RIB40* |
| 103 | VII | XP_001818926 | R | ascomycetes | *Aspergillus oryzae RIB40* |
| 104 | VII | XP_001823618 | R | ascomycetes | *Aspergillus oryzae RIB40* |
| 105 | VII | XP_001827158 | R | ascomycetes | *Aspergillus oryzae RIB40* |
| 106 | VIII | BAE59374 | TE | ascomycetes | *Aspergillus oryzae RIB40* |
| 107 | IV | AAS66004 | TE | ascomycetes | *Aspergillus parasiticus* |
| 108 | IV | Q12053 | TE | ascomycetes | *Aspergillus parasiticus* |
| 109 | III | EYE97768 | TE | ascomycetes | *Aspergillus ruber CBS 135680* |
| 110 | III | EYE98507 | TE | ascomycetes | *Aspergillus ruber CBS 135680* |
| 111 | V | EYE98259 | none | ascomycetes | *Aspergillus ruber CBS 135680* |
| 112 | VII | EYE91696 | R | ascomycetes | *Aspergillus ruber CBS 135680* |
| 113 | IV | AAR32704 | TE | ascomycetes | *Aspergillus sp. L* |
| 114 | I | AGC95321 | TE | ascomycetes | *Aspergillus terreus* |
| 115 | III | BAB88689 | TE | ascomycetes | *Aspergillus terreus* |
| 116 | V | BAB88752 | none | ascomycetes | *Aspergillus terreus* |
| 117 | VII | XP_001212610 | R | ascomycetes | *Aspergillus terreus NIH2624* |
| 118 | III | XP_001216121 | TE | ascomycetes | *Aspergillus terreus NIH2624* |
| 119 | V | XP_001211612 | none | ascomycetes | *Aspergillus terreus NIH2624* |
| 120 | V | XP_001217072 | none | ascomycetes | *Aspergillus terreus NIH2624* |
| 121 | VI | XP_001212807 | TE-like | ascomycetes | *Aspergillus terreus NIH2624* |
| 122 | VII | XP_001216282 | R | ascomycetes | *Aspergillus terreus NIH2624* |
| 123 | VII | XP_001217248 | R | ascomycetes | *Aspergillus terreus NIH2624* |
| 124 | VIII | XP_001210065 | TE | ascomycetes | *Aspergillus terreus NIH2624* |
| 125 | IV | XP_007671613 | TE | ascomycetes | *Baudoinia compniacensis UAMH 10762* |
| 126 | III | EJP64619 | TE | ascomycetes | *Beauveria bassiana ARSEF 2860* |
| 127 | VIII | EJP62792 | TE | ascomycetes | *Beauveria bassiana ARSEF 2860* |
| 128 | V | ENH99769 | none | ascomycetes | *Bipolaris maydis ATCC 48331* |
| 129 | II | EMD96875 | TE | ascomycetes | *Bipolaris maydis C5* |
| 130 | V | EMD89515 | none | ascomycetes | *Bipolaris maydis C5* |
| 131 | V | EMD93898 | none | ascomycetes | *Bipolaris maydis C5* |
| 132 | VII | EMD93081 | R | ascomycetes | *Bipolaris maydis C5* |
| 133 | II | BAD22832 | TE | ascomycetes | *Bipolaris oryzae* |
| 134 | I | XP_007683625 | TE | ascomycetes | *Bipolaris oryzae ATCC 44560* |
| 135 | II | XP_007683730 | TE | ascomycetes | *Bipolaris oryzae ATCC 44560* |
| 136 | IV | XP_007688540 | R | ascomycetes | *Bipolaris oryzae ATCC 44560* |
| 137 | V | XP_007688273 | none | ascomycetes | *Bipolaris oryzae ATCC 44560* |
| 138 | V | XP_007692278 | none | ascomycetes | *Bipolaris oryzae ATCC 44560* |
| 139 | VI | XP_007689067 | TE-like | ascomycetes | *Bipolaris oryzae ATCC 44560* |
| 140 | I | XP_007697886 | TE | ascomycetes | *Bipolaris sorokiniana ND90Pr* |
| 141 | II | XP_007696213 | TE | ascomycetes | *Bipolaris sorokiniana ND90Pr* |
| 142 | IV | XP_007696013 | R | ascomycetes | *Bipolaris sorokiniana ND90Pr* |
| 143 | V | XP_007696688 | none | ascomycetes | *Bipolaris sorokiniana ND90Pr* |
| 144 | V | XP_007701226 | none | ascomycetes | *Bipolaris sorokiniana ND90Pr* |
| 145 | I | EUN21450 | TE | ascomycetes | *Bipolaris victoriae FI3* |
| 146 | II | EUN32222 | TE | ascomycetes | *Bipolaris victoriae FI3* |
| 147 | IV | EUN29146 | R | ascomycetes | *Bipolaris victoriae FI3* |
| 148 | V | EUN25734 | none | ascomycetes | *Bipolaris victoriae FI3* |
| 149 | VII | EUN22347 | R | ascomycetes | *Bipolaris victoriae FI3* |
| 150 | I | XP_007712105 | TE | ascomycetes | *Bipolaris zeicola 26-R-13* |
| 151 | I | XP_007718130 | TE | ascomycetes | *Bipolaris zeicola 26-R-13* |
| 152 | II | XP_007706311 | TE | ascomycetes | *Bipolaris zeicola 26-R-13* |
| 153 | V | XP_007715766 | none | ascomycetes | *Bipolaris zeicola 26-R-13* |
| 154 | VII | XP_007714759 | R | ascomycetes | *Bipolaris zeicola 26-R-13* |
| 155 | V | CCU75801 | none | ascomycetes | *Blumeria graminis f. sp. hordei DH14* |
| 156 | V | EPQ66189 | none | ascomycetes | *Blumeria graminis f. sp. tritici 96224* |
| 157 | I | XP_001559596 | TE | ascomycetes | *Botryotinia fuckeliana B05.10* |
| 158 | II | XP_001547095 | TE | ascomycetes | *Botryotinia fuckeliana B05.10* |
| 159 | II | XP_001554288 | TE | ascomycetes | *Botryotinia fuckeliana B05.10* |
| 160 | V | XP_001553397 | none | ascomycetes | *Botryotinia fuckeliana B05.10* |
| 161 | VI | XP_001550802 | TE-like | ascomycetes | *Botryotinia fuckeliana B05.10* |
| 162 | VII | XP_001559289 | R | ascomycetes | *Botryotinia fuckeliana B05.10* |
| 163 | I | CCD53467 | TE | ascomycetes | *Botryotinia fuckeliana T4* |
| 164 | II | CCD50774 | TE | ascomycetes | *Botryotinia fuckeliana T4* |
| 165 | II | CCD52428 | TE | ascomycetes | *Botryotinia fuckeliana T4* |
| 166 | VI | CCD46020 | TE-like | ascomycetes | *Botryotinia fuckeliana T4* |
| 167 | VI | CCD50766 | TE-like | ascomycetes | *Botryotinia fuckeliana T4* |
| 168 | VI | CCD51172 | TE-like | ascomycetes | *Botryotinia fuckeliana T4* |
| 169 | VI | CCD56082 | TE-like | ascomycetes | *Botryotinia fuckeliana T4* |
| 170 | VII | CCD44950 | R | ascomycetes | *Botryotinia fuckeliana T4* |
| 171 | I | EMR80535 | TE | ascomycetes | *Botrytis cinerea BcDW1* |
| 172 | II | EMR81302 | TE | ascomycetes | *Botrytis cinerea BcDW1* |
| 173 | II | EMR86678 | TE | ascomycetes | *Botrytis cinerea BcDW1* |
| 174 | V | EMR83380 | none | ascomycetes | *Botrytis cinerea BcDW1* |
| 175 | VI | EMR80977 | TE-like | ascomycetes | *Botrytis cinerea BcDW1* |
| 176 | VI | EMR86672 | TE-like | ascomycetes | *Botrytis cinerea BcDW1* |
| 177 | VI | EMR89251 | TE-like | ascomycetes | *Botrytis cinerea BcDW1* |
| 178 | VI | EMR89283 | TE-like | ascomycetes | *Botrytis cinerea BcDW1* |
| 179 | II | GAD99561 | TE | ascomycetes | *Byssochlamys spectabilis No. 5* |
| 180 | VII | GAE00035 | R | ascomycetes | *Byssochlamys spectabilis No. 5* |
| 181 | II | XP_007721308 | TE | ascomycetes | *Capronia coronata CBS 617.96* |
| 182 | II | XP_007728918 | TE | ascomycetes | *Capronia epimyces CBS 606.96* |
| 183 | V | XP_007731035 | none | ascomycetes | *Capronia epimyces CBS 606.96* |
| 184 | VI | XP_007730098 | TE-like | ascomycetes | *Capronia epimyces CBS 606.96* |
| 185 | II | AAO60166 | TE | ascomycetes | *Ceratocystis resinifera* |
| 186 | IV | AAT69682 | TE | ascomycetes | *Cercospora nicotianae* |
| 187 | VIII | EMD42363 | TE | basidiomycetes | *Ceriporiopsis subvermispora B* |
| 188 | I | ACM42403 | TE | ascomycetes | *Chaetomium chiversii* |
| 189 | II | XP_001219763 | TE | ascomycetes | *Chaetomium globosum CBS 148.51* |
| 190 | VI | XP_001228055 | TE-like | ascomycetes | *Chaetomium globosum CBS 148.51* |
| 191 | VII | XP_001227513 | R | ascomycetes | *Chaetomium globosum CBS 148.51* |
| 192 | VII | XP_001227954 | R | ascomycetes | *Chaetomium globosum CBS 148.51* |
| 193 | II | XP_006695455 | TE | ascomycetes | *Chaetomium thermophilum var. thermophilum DSM 1495* |
| 194 | II | ADX36087 | TE | ascomycetes | *Cladonia grayi* |
| 195 | VI | ADM79462 | TE-like | ascomycetes | *Cladonia grayi* |
| 196 | II | AFB81352 | TE | ascomycetes | *Cladonia macilenta* |
| 197 | II | ETI24557 | TE | ascomycetes | *Cladophialophora carrionii CBS 160.54* |
| 198 | V | ETI19899 | none | ascomycetes | *Cladophialophora carrionii CBS 160.54* |
| 199 | II | XP_007740016 | TE | ascomycetes | *Cladophialophora psammophila CBS 110553* |
| 200 | III | XP_007740172 | TE | ascomycetes | *Cladophialophora psammophila CBS 110553* |
| 201 | II | XP_007756951 | TE | ascomycetes | *Cladophialophora yegresii CBS 114405* |
| 202 | V | XP_007754624 | none | ascomycetes | *Cladophialophora yegresii CBS 114405* |
| 203 | V | scf7180000126987_G8647 | none | ascomycetes | *Cladosporium fulvum* |
| 204 | II | AFP89389 | TE | ascomycetes | *Cladosporium phlei* |
| 205 | I | CCE33500 | TE | ascomycetes | *Claviceps purpurea 20.1* |
| 206 | V | CCE31584 | none | ascomycetes | *Claviceps purpurea 20.1* |
| 207 | VI | XP_001243721 | TE-like | ascomycetes | *Coccidioides immitis RS* |
| 208 | VII | XP_001243185 | R | ascomycetes | *Coccidioides immitis RS* |
| 209 | IV | XP_001241406 | TE | ascomycetes | *Coccidioides immitis RS* |
| 210 | VI | EAS33894 | TE-like | ascomycetes | *Coccidioides immitis RS* |
| 211 | VI | XP_001245248 | TE-like | ascomycetes | *Coccidioides immitis RS* |
| 212 | VII | XP_001246130 | R | ascomycetes | *Coccidioides immitis RS* |
| 213 | IV | XP_003070496 | TE | ascomycetes | *Coccidioides posadasii C735 delta SOWgp* |
| 214 | VI | XP_003068966 | TE-like | ascomycetes | *Coccidioides posadasii C735 delta SOWgp* |
| 215 | VI | XP_003071593 | TE-like | ascomycetes | *Coccidioides posadasii C735 delta SOWgp* |
| 216 | VII | XP_003067742 | R | ascomycetes | *Coccidioides posadasii C735 delta SOWgp* |
| 217 | VII | XP_003070229 | R | ascomycetes | *Coccidioides posadasii C735 delta SOWgp* |
| 218 | IV | EFW18011 | TE | ascomycetes | *Coccidioides posadasii str. Silveira* |
| 219 | VI | EFW20973 | TE-like | ascomycetes | *Coccidioides posadasii str. Silveira* |
| 220 | VII | EFW17356 | R | ascomycetes | *Coccidioides posadasii str. Silveira* |
| 221 | VII | EFW23245 | R | ascomycetes | *Coccidioides posadasii str. Silveira* |
| 222 | II | XP_007596282 | TE | ascomycetes | *Colletotrichum fioriniae PJ7* |
| 223 | III | XP_007599710 | TE | ascomycetes | *Colletotrichum fioriniae PJ7* |
| 224 | IV | XP_007592254 | TE | ascomycetes | *Colletotrichum fioriniae PJ7* |
| 225 | I | EQB52988 | TE | ascomycetes | *Colletotrichum gloeosporioides Cg-14* |
| 226 | II | EQB55056 | TE | ascomycetes | *Colletotrichum gloeosporioides Cg-14* |
| 227 | IV | EQB52152 | TE | ascomycetes | *Colletotrichum gloeosporioides Cg-14* |
| 228 | VI | EQB51125 | TE-like | ascomycetes | *Colletotrichum gloeosporioides Cg-14* |
| 229 | VIII | EQB49011 | TE | ascomycetes | *Colletotrichum gloeosporioides Cg-14* |
| 230 | VI | XP_007284106 | TE-like | ascomycetes | *Colletotrichum gloeosporioides Nara gc5* |
| 231 | I | XP_007280259 | TE | ascomycetes | *Colletotrichum gloeosporioides Nara gc5* |
| 232 | II | XP_007287024 | TE | ascomycetes | *Colletotrichum gloeosporioides Nara gc5* |
| 233 | IV | XP_007281131 | TE | ascomycetes | *Colletotrichum gloeosporioides Nara gc5* |
| 234 | VI | XP_007283523 | TE-like | ascomycetes | *Colletotrichum gloeosporioides Nara gc5* |
| 235 | I | EFQ36633 | TE | ascomycetes | *Colletotrichum graminicola M1.001* |
| 236 | I | EFQ36690 | TE | ascomycetes | *Colletotrichum graminicola M1.001* |
| 237 | II | EFQ29059 | TE | ascomycetes | *Colletotrichum graminicola M1.001* |
| 238 | IV | EFQ33691 | TE | ascomycetes | *Colletotrichum graminicola M1.001* |
| 239 | IV | EFQ36810 | R | ascomycetes | *Colletotrichum graminicola M1.001* |
| 240 | V | EFQ33703 | none | ascomycetes | *Colletotrichum graminicola M1.001* |
| 241 | VI | EFQ36407 | TE-like | ascomycetes | *Colletotrichum graminicola M1.001* |
| 242 | VI | EFQ36652 | TE-like | ascomycetes | *Colletotrichum graminicola M1.001* |
| 243 | VII | EFQ34124 | R | ascomycetes | *Colletotrichum graminicola M1.001* |
| 244 | VII | EFQ36707 | R | ascomycetes | *Colletotrichum graminicola M1.001* |
| 245 | II | CCF45141 | TE | ascomycetes | *Colletotrichum higginsianum* |
| 246 | IV | CCF41085 | TE | ascomycetes | *Colletotrichum higginsianum* |
| 247 | II | BAA18956 | TE | ascomycetes | *Colletotrichum lagenaria* |
| 248 | I | ENH83455 | TE | ascomycetes | *Colletotrichum orbiculare MAFF 240422* |
| 249 | I | ENH86452 | TE | ascomycetes | *Colletotrichum orbiculare MAFF 240422* |
| 250 | II | ENH81867 | TE | ascomycetes | *Colletotrichum orbiculare MAFF 240422* |
| 251 | IV | ENH81662 | TE | ascomycetes | *Colletotrichum orbiculare MAFF 240422* |
| 252 | IV | ENH84744 | TE | ascomycetes | *Colletotrichum orbiculare MAFF 240422* |
| 253 | VI | ENH84250 | TE-like | ascomycetes | *Colletotrichum orbiculare MAFF 240422* |
| 254 | VIII | ENH82425 | TE | ascomycetes | *Colletotrichum orbiculare MAFF 240422* |
| 255 | VIII | XP_007775190 | TE | basidiomycetes | *Coniophora_puteana_RWD-64-598_SS2* |
| 256 | II | EON65175 | TE | ascomycetes | *Coniosporium apollinis CBS 100218* |
| 257 | III | EON65343 | TE | ascomycetes | *Coniosporium apollinis CBS 100218* |
| 258 | VIII | XP_001835415 | TE | basidiomycetes | *Coprinopsis cinerea okayama7#130* |
| 259 | III | XP_006668876 | TE | ascomycetes | *Cordyceps militaris CM01* |
| 260 | IV | XP_006667138 | TE | ascomycetes | *Cordyceps militaris CM01* |
| 261 | II | ETN44617 | TE | ascomycetes | *Cyphellophora europaea CBS 101466* |
| 262 | II | ADP05113 | TE | ascomycetes | *Daldinia eschscholtzii* |
| 263 | VIII | XP_007368302 | TE | basidiomycetes | *Dichomitus squalens LYAD-421 SS1* |
| 264 | II | ACH72076 | TE | ascomycetes | *Dirinaria applanata* |
| 265 | II | ABS85549 | TE | ascomycetes | *Dothiorella aegiceri* |
| 266 | II | EME39782 | TE | ascomycetes | *Dothistroma septosporum NZE10* |
| 267 | II | ABU63483 | TE | ascomycetes | *Elsinoe fawcettii* |
| 268 | II | XP_007804330 | TE | ascomycetes | *Endocarpon pusillum Z07020* |
| 269 | III | XP_007785431 | TE | ascomycetes | *Endocarpon pusillum Z07020* |
| 270 | V | XP_007786761 | none | ascomycetes | *Endocarpon pusillum Z07020* |
| 271 | VII | XP_007800756 | R | ascomycetes | *Endocarpon pusillum Z07020* |
| 272 | VII | XP_007801017 | R | ascomycetes | *Endocarpon pusillum Z07020* |
| 273 | II | XP_007796413 | TE | ascomycetes | *Eutypa lata UCREL1* |
| 274 | V | XP_007793674 | none | ascomycetes | *Eutypa lata UCREL1* |
| 275 | VI | XP_007790426 | TE-like | ascomycetes | *Eutypa lata UCREL1* |
| 276 | VII | XP_007793245 | R | ascomycetes | *Eutypa lata UCREL1* |
| 277 | VII | XP_007793864 | R | ascomycetes | *Eutypa lata UCREL1* |
| 278 | II | AAD31436 | TE | ascomycetes | *Exophiala dermatitidis* |
| 279 | II | AAN75188 | TE | ascomycetes | *Exophiala lecanii-corni* |
| 280 | VIII | CCM00101 | TE | basidiomycetes | *Fibroporia radiculosa* |
| 281 | VIII | XP_007262597 | TE | basidiomycetes | *Fomitiporia mediterranea MF3/22* |
| 282 | VIII | EPS94471 | TE | basidiomycetes | *Fomitopsis pinicola FP-58527 SS1* |
| 283 | III | CAB92399 | TE | ascomycetes | *Fusarium fujikuroi* |
| 284 | IV | CCE67070 | R | ascomycetes | *Fusarium fujikuroi* |
| 285 | VII | CCT76054 | R | ascomycetes | *Fusarium fujikuroi IMI 58289* |
| 286 | I | ABB90282 | TE | ascomycetes | *Fusarium graminearum* |
| 287 | III | AAU10633 | TE | ascomycetes | *Fusarium graminearum* |
| 288 | IV | EYB26831 | R | ascomycetes | *Fusarium graminearum* |
| 289 | VIII | EYB32182 | TE | ascomycetes | *Fusarium graminearum* |
| 290 | III | ESU07748 | TE | ascomycetes | *Fusarium graminearum PH-1* |
| 291 | IV | ESU15719 | R | ascomycetes | *Fusarium graminearum PH-1* |
| 292 | VIII | XP_384140 | TE | ascomycetes | *Fusarium graminearum PH-1* |
| 293 | IV | EXL68993 | R | ascomycetes | *Fusarium oxysporum f. sp. conglutinans race 2 54008* |
| 294 | III | ENH68137 | TE | ascomycetes | *Fusarium oxysporum f. sp. cubense race 1* |
| 295 | IV | ENH69483 | R | ascomycetes | *Fusarium oxysporum f. sp. cubense race 1* |
| 296 | III | EMT69321 | TE | ascomycetes | *Fusarium oxysporum f. sp. cubense race 4* |
| 297 | III | EXL92030 | TE | ascomycetes | *Fusarium oxysporum f. sp. cubense tropical race 4 54006* |
| 298 | IV | EXM03524 | R | ascomycetes | *Fusarium oxysporum f. sp. cubense tropical race 4 54006* |
| 299 | III | EWZ80090 | TE | ascomycetes | *Fusarium oxysporum f. sp. lycopersici MN25* |
| 300 | IV | EWZ99873 | R | ascomycetes | *Fusarium oxysporum f. sp. lycopersici MN25* |
| 301 | III | EXK38465 | TE | ascomycetes | *Fusarium oxysporum f. sp. melonis 26406* |
| 302 | IV | EXK32837 | R | ascomycetes | *Fusarium oxysporum f. sp. melonis 26406* |
| 303 | III | EXA44005 | TE | ascomycetes | *Fusarium oxysporum f. sp. pisi HDV247* |
| 304 | IV | EXA46686 | R | ascomycetes | *Fusarium oxysporum f. sp. pisi HDV247* |
| 305 | III | EXL55894 | TE | ascomycetes | *Fusarium oxysporum f. sp. radicis-lycopersici 26381* |
| 306 | IV | EXL58547 | R | ascomycetes | *Fusarium oxysporum f. sp. radicis-lycopersici 26381* |
| 307 | III | EXK83376 | TE | ascomycetes | *Fusarium oxysporum f. sp. raphani 54005* |
| 308 | IV | EXK80558 | R | ascomycetes | *Fusarium oxysporum f. sp. raphani 54005* |
| 309 | III | EXM24735 | TE | ascomycetes | *Fusarium oxysporum f. sp. vasinfectum 25433* |
| 310 | IV | EXM20110 | R | ascomycetes | *Fusarium oxysporum f. sp. vasinfectum 25433* |
| 311 | III | EWZ36433 | TE | ascomycetes | *Fusarium oxysporum Fo47* |
| 312 | IV | EWZ43044 | R | ascomycetes | *Fusarium oxysporum Fo47* |
| 313 | IV | EGU74198 | R | ascomycetes | *Fusarium oxysporum Fo5176* |
| 314 | III | EWY89850 | TE | ascomycetes | *Fusarium oxysporum FOSC 3-a* |
| 315 | IV | EWY95164 | R | ascomycetes | *Fusarium oxysporum FOSC 3-a* |
| 316 | I | EKJ72968 | TE | ascomycetes | *Fusarium pseudograminearum CS3096* |
| 317 | III | EKJ76027 | TE | ascomycetes | *Fusarium pseudograminearum CS3096* |
| 318 | IV | EKJ76363 | R | ascomycetes | *Fusarium pseudograminearum CS3096* |
| 319 | VIII | EKJ74560 | TE | ascomycetes | *Fusarium pseudograminearum CS3096* |
| 320 | III | EWG41233 | TE | ascomycetes | *Fusarium verticillioides 7600* |
| 321 | IV | EWG41617 | R | ascomycetes | *Fusarium verticillioides 7600* |
| 322 | II | EJT80408 | TE | ascomycetes | *Gaeumannomyces graminis var. tritici R3-111a-1* |
| 323 | V | EJT69423 | none | ascomycetes | *Gaeumannomyces graminis var. tritici R3-111a-1* |
| 324 | II | AAN59953 | TE | ascomycetes | *Glarea lozoyensis* |
| 325 | VII | EHK98549 | R | ascomycetes | *Glarea lozoyensis 74030* |
| 326 | I | EHK97626 | TE | ascomycetes | *Glarea lozoyensis 74030* |
| 327 | II | EHL00222 | TE | ascomycetes | *Glarea lozoyensis 74030* |
| 328 | I | EPE32109 | TE | ascomycetes | *Glarea lozoyensis ATCC 20868* |
| 329 | I | EPE35573 | TE | ascomycetes | *Glarea lozoyensis ATCC 20868* |
| 330 | II | EPE33704 | TE | ascomycetes | *Glarea lozoyensis ATCC 20868* |
| 331 | II | EPE34890 | TE | ascomycetes | *Glarea lozoyensis ATCC 20868* |
| 332 | IV | EPE29990 | TE | ascomycetes | *Glarea lozoyensis ATCC 20868* |
| 333 | VII | EPE28681 | R | ascomycetes | *Glarea lozoyensis ATCC 20868* |
| 334 | VIII | XP_007866703 | TE | basidiomycetes | *Gloeophyllum_trabeum_ATCC_11539* |
| 335 | VIII | BAO20284 | TE | basidiomycetes | *Grifola frondosa* |
| 336 | VII | EFX04833 | R | ascomycetes | *Grosmannia clavigera kw1407* |
| 337 | II | EFX02617 | TE | ascomycetes | *Grosmannia clavigera kw1407* |
| 338 | IV | EFX00827 | TE | ascomycetes | *Grosmannia clavigera kw1407* |
| 339 | V | EFX04268 | none | ascomycetes | *Grosmannia clavigera kw1407* |
| 340 | VIII | ETW82342 | TE | basidiomycetes | *Heterobasidion irregulare TC 32-1* |
| 341 | VIII | ETW82343 | TE | basidiomycetes | *Heterobasidion irregulare TC 32-1* |
| 342 | VII | AFO67256 | R | ascomycetes | *Hypogymnia physodes* |
| 343 | VII | AFO67255 | R | ascomycetes | *Hypogymnia physodes* |
| 344 | I | ACD39762 | TE | ascomycetes | *Hypomyces subiculosus* |
| 345 | VIII | XP_001876029 | TE | basidiomycetes | *Laccaria bicolor S238N-H82* |
| 346 | I | AHV78247 | TE | ascomycetes | *Lasiodiplodia theobromae* |
| 347 | IV | AAS92537 | TE | ascomycetes | *Leptosphaeria maculans* |
| 348 | II | XP_003841919 | TE | ascomycetes | *Leptosphaeria maculans JN3* |
| 349 | IV | XP_003842432 | TE | ascomycetes | *Leptosphaeria maculans JN3* |
| 350 | V | EKG18431 | none | ascomycetes | *Macrophomina phaseolina MS6* |
| 351 | VI | EKG18751 | TE-like | ascomycetes | *Macrophomina phaseolina MS6* |
| 352 | VIII | EKG09551 | TE | ascomycetes | *Macrophomina phaseolina MS6* |
| 353 | I | XP_003715799 | TE | ascomycetes | *Magnaporthe oryzae 70-15* |
| 354 | I | XP_003718865 | TE | ascomycetes | *Magnaporthe oryzae 70-15* |
| 355 | II | XP_003715434 | TE | ascomycetes | *Magnaporthe oryzae 70-15* |
| 356 | IV | XP_003718644 | TE | ascomycetes | *Magnaporthe oryzae 70-15* |
| 357 | VII | XP_003717493 | R | ascomycetes | *Magnaporthe oryzae 70-15* |
| 358 | VII | ELQ61531 | R | ascomycetes | *Magnaporthe oryzae P131* |
| 359 | IV | ELQ68526 | TE | ascomycetes | *Magnaporthe oryzae P131* |
| 360 | I | ELQ42257 | TE | ascomycetes | *Magnaporthe oryzae Y34* |
| 361 | II | ELQ39536 | TE | ascomycetes | *Magnaporthe oryzae Y34* |
| 362 | IV | ELQ42604 | TE | ascomycetes | *Magnaporthe oryzae Y34* |
| 363 | VII | ELQ38934 | R | ascomycetes | *Magnaporthe oryzae Y34* |
| 364 | I | XP_007294614 | TE | ascomycetes | *Marssonina brunnea f. sp. 'multigermtubi' MB_m1* |
| 365 | II | XP_007288149 | TE | ascomycetes | *Marssonina brunnea f. sp. 'multigermtubi' MB_m1* |
| 366 | II | XP_007295542 | TE | ascomycetes | *Marssonina brunnea f. sp. 'multigermtubi' MB_m1* |
| 367 | I | ACD39770 | TE | ascomycetes | *Metacordyceps chlamydosporia* |
| 368 | III | XP_007806517 | TE | ascomycetes | *Metarhizium acridum CQMa 102* |
| 369 | III | XP_007807862 | TE | ascomycetes | *Metarhizium acridum CQMa 102* |
| 370 | III | XP_007811725 | TE | ascomycetes | *Metarhizium acridum CQMa 102* |
| 371 | III | XP_007815650 | TE | ascomycetes | *Metarhizium acridum CQMa 102* |
| 372 | V | XP_007810456 | none | ascomycetes | *Metarhizium acridum CQMa 102* |
| 373 | VII | XP_007810891 | R | ascomycetes | *Metarhizium acridum CQMa 102* |
| 374 | VII | XP_007816051 | R | ascomycetes | *Metarhizium acridum CQMa 102* |
| 375 | III | XP_007819428 | TE | ascomycetes | *Metarhizium anisopliae ARSEF 23* |
| 376 | III | XP_007823934 | TE | ascomycetes | *Metarhizium anisopliae ARSEF 23* |
| 377 | III | XP_007826081 | TE | ascomycetes | *Metarhizium anisopliae ARSEF 23* |
| 378 | V | XP_007822764 | none | ascomycetes | *Metarhizium anisopliae ARSEF 23* |
| 379 | V | XP_007825109 | none | ascomycetes | *Metarhizium anisopliae ARSEF 23* |
| 380 | VII | XP_007824114 | R | ascomycetes | *Metarhizium anisopliae ARSEF 23* |
| 381 | VII | XP_007826136 | R | ascomycetes | *Metarhizium anisopliae ARSEF 23* |
| 382 | VII | EXU95978 | R | ascomycetes | *Metarhizium robertsii* |
| 383 | III | EXU95796 | TE | ascomycetes | *Metarhizium robertsii* |
| 384 | III | EXU98524 | TE | ascomycetes | *Metarhizium robertsii* |
| 385 | III | EXV02536 | TE | ascomycetes | *Metarhizium robertsii* |
| 386 | VII | AGN71604 | R | ascomycetes | *Monascus pilosus* |
| 387 | II | CAC94008 | TE | ascomycetes | *Monascus purpureus* |
| 388 | VII | BAD44749 | R | ascomycetes | *Monascus purpureus* |
| 389 | VIII | XP_007844087 | TE | basidiomycetes | *Moniliophthora_roreri_MCA_2997* |
| 390 | VIII | XP_007844893 | TE | basidiomycetes | *Moniliophthora_roreri_MCA_2997* |
| 391 | VIII | XP_007850268 | TE | basidiomycetes | *Moniliophthora_roreri_MCA_2997* |
| 392 | VIII | XP_007850276 | TE | basidiomycetes | *Moniliophthora_roreri_MCA_2997* |
| 393 | II | XP_003661126 | TE | ascomycetes | *Myceliophthora thermophila ATCC 42464* |
| 394 | III | XP_003666434 | TE | ascomycetes | *Myceliophthora thermophila ATCC 42464* |
| 395 | V | XP_003663601 | none | ascomycetes | *Myceliophthora thermophila ATCC 42464* |
| 396 | VIII | XP_003664878 | TE | ascomycetes | *Myceliophthora thermophila ATCC 42464* |
| 397 | IV | ADO14690 | TE | ascomycetes | *Mycosphaerella coffeicola* |
| 398 | IV | AAZ95017 | TE | ascomycetes | *Mycosphaerella pini* |
| 399 | III | AAS48892 | TE | ascomycetes | *Nectria haematococca* |
| 400 | III | XP_003045916 | TE | ascomycetes | *Nectria haematococca mpVI 77-13-4* |
| 401 | IV | XP_003039929 | R | ascomycetes | *Nectria haematococca mpVI 77-13-4* |
| 402 | II | XP_007584358 | TE | ascomycetes | *Neofusicoccum parvum UCRNP2* |
| 403 | VII | XP_007579490 | R | ascomycetes | *Neofusicoccum parvum UCRNP2* |
| 404 | III | XP_001261235 | TE | ascomycetes | *Neosartorya fischeri NRRL 181* |
| 405 | V | XP_001262597 | none | ascomycetes | *Neosartorya fischeri NRRL 181* |
| 406 | V | XP_001266579 | none | ascomycetes | *Neosartorya fischeri NRRL 181* |
| 407 | V | XP_001266594 | none | ascomycetes | *Neosartorya fischeri NRRL 181* |
| 408 | V | XP_001267621 | none | ascomycetes | *Neosartorya fischeri NRRL 181* |
| 409 | VII | XP_001258915 | R | ascomycetes | *Neosartorya fischeri NRRL 181* |
| 410 | II | EAA31350 | TE | ascomycetes | *Neurospora crassa OR74A* |
| 411 | II | XP_960586 | TE | ascomycetes | *Neurospora crassa OR74A* |
| 412 | II | EGO61342 | TE | ascomycetes | *Neurospora tetrasperma FGSC 2508* |
| 413 | II | EGZ74641 | TE | ascomycetes | *Neurospora tetrasperma FGSC 2509* |
| 414 | II | AAD38786 | TE | ascomycetes | *Nodulisporium sp. ATCC74245* |
| 415 | II | ABD47522 | TE | ascomycetes | *Ophiostoma piceae* |
| 416 | II | AEE65372 | TE | ascomycetes | *Peltigera membranacea* |
| 417 | II | AEE65374 | TE | ascomycetes | *Peltigera membranacea* |
| 418 | V | ADI24926 | none | ascomycetes | *Penicillium aethiopicum* |
| 419 | V | ADI24953 | none | ascomycetes | *Penicillium aethiopicum* |
| 420 | VI | ADY00130 | TE-like | ascomycetes | *Penicillium brevicompactum* |
| 421 | VII | XP_002567553 | R | ascomycetes | *Penicillium chrysogenum Wisconsin 54-1255* |
| 422 | I | XP_002568275 | TE | ascomycetes | *Penicillium chrysogenum Wisconsin 54-1255* |
| 423 | III | XP_002568608 | TE | ascomycetes | *Penicillium chrysogenum Wisconsin 54-1255* |
| 424 | III | EKV10885 | TE | ascomycetes | *Penicillium digitatum PHI26* |
| 425 | VI | EKV16250 | TE-like | ascomycetes | *Penicillium digitatum PHI26* |
| 426 | VII | EKV06857 | R | ascomycetes | *Penicillium digitatum PHI26* |
| 427 | III | EPS34527 | TE | ascomycetes | *Penicillium oxalicum 114-2* |
| 428 | V | EPS34273 | none | ascomycetes | *Penicillium oxalicum 114-2* |
| 429 | III | CDM29654 | TE | ascomycetes | *Penicillium roqueforti* |
| 430 | VI | CDM27382 | TE-like | ascomycetes | *Penicillium roqueforti* |
| 431 | VI | CDM36726 | TE-like | ascomycetes | *Penicillium roqueforti* |
| 432 | II | XP_007833873 | TE | ascomycetes | *Pestalotiopsis fici W106-1* |
| 433 | IV | XP_007833333 | R | ascomycetes | *Pestalotiopsis fici W106-1* |
| 434 | V | XP_007837596 | none | ascomycetes | *Pestalotiopsis fici W106-1* |
| 435 | II | AGT56219 | TE | ascomycetes | *Pestalotiopsis malicola* |
| 436 | II | XP_001802212 | TE | ascomycetes | *Phaeosphaeria nodorum SN15* |
| 437 | III | XP_001798591 | TE | ascomycetes | *Phaeosphaeria nodorum SN15* |
| 438 | IV | XP_001798923 | TE | ascomycetes | *Phaeosphaeria nodorum SN15* |
| 439 | V | XP_001805964 | none | ascomycetes | *Phaeosphaeria nodorum SN15* |
| 440 | VI | XP_001797375 | TE-like | ascomycetes | *Phaeosphaeria nodorum SN15* |
| 441 | VI | XP_001906796 | TE-like | ascomycetes | *Podospora anserina S mat+* |
| 442 | II | XP_001910795 | TE | ascomycetes | *Podospora anserina S mat+* |
| 443 | IV | XP_001911528 | TE | ascomycetes | *Podospora anserina S mat+* |
| 444 | VIII | XP_001903585 | TE | ascomycetes | *Podospora anserina S mat+* |
| 445 | II | XP_007931490 | TE | ascomycetes | *Pseudocercospora fijiensis CIRAD86* |
| 446 | V | XP_007929626 | none | ascomycetes | *Pseudocercospora fijiensis CIRAD86* |
| 447 | II | ELR06213 | TE | ascomycetes | *Pseudogymnoascus destructans 20631-21* |
| 448 | III | ELR07706 | TE | ascomycetes | *Pseudogymnoascus destructans 20631-21* |
| 449 | III | ELR10234 | TE | ascomycetes | *Pseudogymnoascus destructans 20631-21* |
| 450 | V | ELR08155 | none | ascomycetes | *Pseudogymnoascus destructans 20631-21* |
| 451 | VII | ELR07152 | R | ascomycetes | *Pseudogymnoascus destructans 20631-21* |
| 452 | VIII | XP_007386370 | TE | basidiomycetes | *Punctularia strigosozonata HHB-11173 SS5* |
| 453 | II | XP_003298917 | TE | ascomycetes | *Pyrenophora teres f. teres 0-1* |
| 454 | IV | XP_003306836 | R | ascomycetes | *Pyrenophora teres f. teres 0-1* |
| 455 | V | XP_003296346 | none | ascomycetes | *Pyrenophora teres f. teres 0-1* |
| 456 | VI | XP_003300445 | TE-like | ascomycetes | *Pyrenophora teres f. teres 0-1* |
| 457 | VI | XP_003301870 | TE-like | ascomycetes | *Pyrenophora teres f. teres 0-1* |
| 458 | VII | XP_003299580 | R | ascomycetes | *Pyrenophora teres f. teres 0-1* |
| 459 | I | XP_001934718 | TE | ascomycetes | *Pyrenophora tritici-repentis Pt-1C-BFP* |
| 460 | II | XP_001933656 | TE | ascomycetes | *Pyrenophora tritici-repentis Pt-1C-BFP* |
| 461 | V | XP_001933041 | none | ascomycetes | *Pyrenophora tritici-repentis Pt-1C-BFP* |
| 462 | VIII | XP_001939987 | TE | ascomycetes | *Pyrenophora tritici-repentis Pt-1C-BFP* |
| 463 | VIII | CCX04910 | TE | ascomycetes | *Pyronema omphalodes CBS 100304* |
| 464 | II | BAO58974 | TE | ascomycetes | *Rosellinia necatrix* |
| 465 | II | ABG91136 | TE | ascomycetes | *Rusavskia elegans* |
| 466 | I | AHV78253 | TE | ascomycetes | *Sarocladium zeae* |
| 467 | VIII | XP_003038401 | TE | basidiomycetes | *Schizophyllum commune H4-8* |
| 468 | II | ESZ91707 | TE | ascomycetes | *Sclerotinia borealis F-4157* |
| 469 | II | ESZ92457 | TE | ascomycetes | *Sclerotinia borealis F-4157* |
| 470 | IV | ESZ99281 | TE | ascomycetes | *Sclerotinia borealis F-4157* |
| 471 | V | ESZ98980 | none | ascomycetes | *Sclerotinia borealis F-4157* |
| 472 | VI | ESZ91464 | TE-like | ascomycetes | *Sclerotinia borealis F-4157* |
| 473 | II | XP_001585805 | TE | ascomycetes | *Sclerotinia sclerotiorum 1980 UF-70* |
| 474 | II | XP_001586760 | TE | ascomycetes | *Sclerotinia sclerotiorum 1980 UF-70* |
| 475 | IV | XP_001592760 | TE | ascomycetes | *Sclerotinia sclerotiorum 1980 UF-70* |
| 476 | VIII | EGN93845 | TE | basidiomycetes | *Serpula lacrymans var. lacrymans S7.3* |
| 477 | VIII | XP_007323934 | TE | basidiomycetes | *Serpula lacrymans var. lacrymans S7.9* |
| 478 | I | EOA86107 | TE | ascomycetes | *Setosphaeria turcica Et28A* |
| 479 | II | EOA89647 | TE | ascomycetes | *Setosphaeria turcica Et28A* |
| 480 | IV | EOA85517 | R | ascomycetes | *Setosphaeria turcica Et28A* |
| 481 | V | EOA88807 | none | ascomycetes | *Setosphaeria turcica Et28A* |
| 482 | VI | EOA83736 | TE-like | ascomycetes | *Setosphaeria turcica Et28A* |
| 483 | VI | EOA85421 | TE-like | ascomycetes | *Setosphaeria turcica Et28A* |
| 484 | II | CAM35471 | TE | ascomycetes | *Sordaria macrospora* |
| 485 | II | EMF09017 | TE | ascomycetes | *Sphaerulina musiva SO2202* |
| 486 | V | EMF17386 | none | ascomycetes | *Sphaerulina musiva SO2202* |
| 487 | VIII | EMF08276 | TE | ascomycetes | *Sphaerulina musiva SO2202* |
| 488 | II | ERS95912 | TE | ascomycetes | *Sporothrix schenckii ATCC 58251* |
| 489 | VIII | XP_007307184 | TE | ascomycetes | *Stereum hirsutum FP-91666 SS1* |
| 490 | VIII | EIM84127 | TE | basidiomycetes | *Stereum hirsutum FP-91666 SS1* |
| 491 | VIII | XP_007300189 | TE | basidiomycetes | *Stereum hirsutum FP-91666 SS1* |
| 492 | VII | ADH01671 | R | ascomycetes | *Talaromyces marneffei* |
| 493 | I | XP_002146110 | TE | ascomycetes | *Talaromyces marneffei ATCC 18224* |
| 494 | I | XP_002151003 | TE | ascomycetes | *Talaromyces marneffei ATCC 18224* |
| 495 | II | XP_002149119 | TE | ascomycetes | *Talaromyces marneffei ATCC 18224* |
| 496 | III | XP_002145792 | TE | ascomycetes | *Talaromyces marneffei ATCC 18224* |
| 497 | III | XP_002147717 | TE | ascomycetes | *Talaromyces marneffei ATCC 18224* |
| 498 | V | XP_002144865 | none | ascomycetes | *Talaromyces marneffei ATCC 18224* |
| 499 | V | XP_002149615 | none | ascomycetes | *Talaromyces marneffei ATCC 18224* |
| 500 | VI | XP_002145720 | TE-like | ascomycetes | *Talaromyces marneffei ATCC 18224* |
| 501 | VII | XP_002149769 | R | ascomycetes | *Talaromyces marneffei ATCC 18224* |
| 502 | VII | DAA64703 | R | ascomycetes | *Talaromyces stipitatus* |
| 503 | I | XP_002488697 | TE | ascomycetes | *Talaromyces stipitatus ATCC 10500* |
| 504 | III | XP_002478062 | TE | ascomycetes | *Talaromyces stipitatus ATCC 10500* |
| 505 | III | XP_002481882 | TE | ascomycetes | *Talaromyces stipitatus ATCC 10500* |
| 506 | IV | XP_002483004 | TE | ascomycetes | *Talaromyces stipitatus ATCC 10500* |
| 507 | V | XP_002482902 | none | ascomycetes | *Talaromyces stipitatus ATCC 10500* |
| 508 | V | XP_002482968 | none | ascomycetes | *Talaromyces stipitatus ATCC 10500* |
| 509 | VI | XP_002339967 | TE-like | ascomycetes | *Talaromyces stipitatus ATCC 10500* |
| 510 | VII | XP_002340038 | R | ascomycetes | *Talaromyces stipitatus ATCC 10500* |
| 511 | VII | XP_002340070 | R | ascomycetes | *Talaromyces stipitatus ATCC 10500* |
| 512 | VII | XP_002481993 | R | ascomycetes | *Talaromyces stipitatus ATCC 10500* |
| 513 | VII | XP_002487778 | R | ascomycetes | *Talaromyces stipitatus ATCC 10500* |
| 514 | VIII | XP_002478017 | TE | ascomycetes | *Talaromyces stipitatus ATCC 10500* |
| 515 | VIII | XP_002486858 | TE | ascomycetes | *Talaromyces stipitatus ATCC 10500* |
| 516 | II | XP_003648614 | TE | ascomycetes | *Thielavia terrestris NRRL 8126* |
| 517 | IV | XP_003656937 | TE | ascomycetes | *Thielavia terrestris NRRL 8126* |
| 518 | V | XP_003652581 | none | ascomycetes | *Thielavia terrestris NRRL 8126* |
| 519 | VI | XP_003653746 | TE-like | ascomycetes | *Thielavia terrestris NRRL 8126* |
| 520 | VII | XP_003651141 | R | ascomycetes | *Thielavia terrestris NRRL 8126* |
| 521 | VII | XP_003653735 | R | ascomycetes | *Thielavia terrestris NRRL 8126* |
| 522 | VII | XP_003654933 | R | ascomycetes | *Thielavia terrestris NRRL 8126* |
| 523 | II | XP_007912544 | TE | ascomycetes | *Togninia minima UCRPA7* |
| 524 | VII | XP_007915823 | R | ascomycetes | *Togninia minima UCRPA7* |
| 525 | VIII | EIW56045 | TE | basidiomycetes | *Trametes versicolor FP-101664 SS1* |
| 526 | I | EHK49847 | TE | ascomycetes | *Trichoderma atroviride IMI 206040* |
| 527 | III | EHK46843 | TE | ascomycetes | *Trichoderma atroviride IMI 206040* |
| 528 | V | EHK46042 | none | ascomycetes | *Trichoderma atroviride IMI 206040* |
| 529 | VI | EHK42299 | TE-like | ascomycetes | *Trichoderma atroviride IMI 206040* |
| 530 | VII | EHK44445 | R | ascomycetes | *Trichoderma atroviride IMI 206040* |
| 531 | III | XP_006969537 | TE | ascomycetes | *Trichoderma reesei QM6a* |
| 532 | V | XP_006969240 | none | ascomycetes | *Trichoderma reesei QM6a* |
| 533 | VI | XP_006964226 | TE-like | ascomycetes | *Trichoderma reesei QM6a* |
| 534 | VII | XP_006961156 | R | ascomycetes | *Trichoderma reesei QM6a* |
| 535 | VI | ETS03185 | TE-like | ascomycetes | *Trichoderma reesei RUT C-30* |
| 536 | III | EHK20748 | TE | ascomycetes | *Trichoderma virens Gv29-8* |
| 537 | V | EHK20655 | none | ascomycetes | *Trichoderma virens Gv29-8* |
| 538 | VI | EHK23001 | TE-like | ascomycetes | *Trichoderma virens Gv29-8* |
| 539 | III | EGE06072 | TE | ascomycetes | *Trichophyton equinum CBS 127.97* |
| 540 | IV | EGE06793 | TE | ascomycetes | *Trichophyton equinum CBS 127.97* |
| 541 | V | EGE06343 | none | ascomycetes | *Trichophyton equinum CBS 127.97* |
| 542 | VII | EGE04288 | R | ascomycetes | *Trichophyton equinum CBS 127.97* |
| 543 | IV | EZF36469 | TE | ascomycetes | *Trichophyton interdigitale H6* |
| 544 | VII | EZF36181 | R | ascomycetes | *Trichophyton interdigitale H6* |
| 545 | IV | KDB24468 | TE | ascomycetes | *Trichophyton interdigitale MR816* |
| 546 | V | KDB28089 | none | ascomycetes | *Trichophyton interdigitale MR816* |
| 547 | IV | XP_003235798 | TE | ascomycetes | *Trichophyton rubrum CBS 118892* |
| 548 | V | XP_003231059 | none | ascomycetes | *Trichophyton rubrum CBS 118892* |
| 549 | VII | XP_003234762 | R | ascomycetes | *Trichophyton rubrum CBS 118892* |
| 550 | V | EZG11077 | none | ascomycetes | *Trichophyton rubrum CBS 735.88* |
| 551 | VII | EZG06712 | R | ascomycetes | *Trichophyton rubrum CBS 735.88* |
| 552 | VII | EZF23313 | R | ascomycetes | *Trichophyton rubrum MR850* |
| 553 | V | EZF78756 | none | ascomycetes | *Trichophyton soudanense CBS 452.61* |
| 554 | VII | EZF74361 | R | ascomycetes | *Trichophyton soudanense CBS 452.61* |
| 555 | III | EGD96701 | TE | ascomycetes | *Trichophyton tonsurans CBS 112818* |
| 556 | IV | EGD94040 | TE | ascomycetes | *Trichophyton tonsurans CBS 112818* |
| 557 | V | EGD99348 | none | ascomycetes | *Trichophyton tonsurans CBS 112818* |
| 558 | VII | EGD97507 | R | ascomycetes | *Trichophyton tonsurans CBS 112818* |
| 559 | IV | XP_003021298 | TE | ascomycetes | *Trichophyton verrucosum HKI 0517* |
| 560 | V | XP_003025446 | none | ascomycetes | *Trichophyton verrucosum HKI 0517* |
| 561 | VII | XP_003023417 | R | ascomycetes | *Trichophyton verrucosum HKI 0517* |
| 562 | VI | XP_002542778 | TE-like | ascomycetes | *Uncinocarpus reesii 1704* |
| 563 | I | AGI60158 | TE | ascomycetes | *Usnea longissima* |
| 564 | II | AEX30306 | TE | ascomycetes | *Usnea longissima* |
| 565 | V | AGI60157 | none | ascomycetes | *Usnea longissima* |
| 566 | VI | AGI60156 | TE-like | ascomycetes | *Usnea longissima* |
| 567 | II | XP_003008898 | TE | ascomycetes | *Verticillium alfalfae VaMs.102* |
| 568 | II | EGY13508 | TE | ascomycetes | *Verticillium dahliae VdLs.17* |
| 569 | III | KDB16994 | TE | ascomycetes | *Villosiclava virens* |
| 570 | VII | KDB13139 | R | ascomycetes | *Villosiclava virens* |
| 571 | VIII | AEF32750 | TE | basidiomycetes | *Volvariella volvacea* |
| 572 | II | AAM93545 | TE | ascomycetes | *Xylaria sp. BCC 1067* |
| 573 | II | XP_003848644 | TE | ascomycetes | *Zymoseptoria tritici IPO323* |
| 574 | VI | XP_003855816 | TE-like | ascomycetes | *Zymoseptoria tritici IPO323* |
