# Supplementary material for: Analysis of the Sequences, Structures, and Functions of Product-Releasing Enzyme Domains in Fungal Polyketide Synthases
Source: Front Microbiol. 2017 Sep 4;8:1685. doi: 10.3389/fmicb.2017.01685 (PMC5591372; doi:10.3389/fmicb.2017.01685)
Supplement: Supplementary file 4 [file Table_2.DOCX]

# Table S2. List of 58 NR-PKSs related to known polyketides.

| **S/N** | **Group** | **Accession No.** | **Protein Name** | **Products** | **Catalytic mechanisms** | **Ref.** |
| --- | --- | --- | --- | --- | --- | --- |
| 1 | I | XP_681178 | *Aspergillus nidulans* OrsA | Lecanoric acid | TE (cross-coupling) | ([Gressler et al., 2015](#_ENREF_20)) |
| 2 | I | AGC95321 | *Aspergillus terreus* AtCURS2 | 10,11-Dehydrocurvularin | TE (macrolactone closure) | ([Xu et al., 2013a](#_ENREF_45)) |
| 3 | I | ACM42403 | *Chaetomium chiversii* RADS2 | Radicicol | TE (macrolactone closure) | ([Xu et al., 2013b](#_ENREF_46)) |
| 4 | I | ABB90282 | *Fusarium graminearum* PKS13 | Zearalenone | TE (macrolactone closure) | ([Wang et al., 2009](#_ENREF_43)) |
| 5 | I | ACD39762 | *Hypomyces subiculosus* Hpm3 | Hypothemycin | TE (macrolactone closure) | ([Reeves et al., 2008](#_ENREF_34)) |
| 6 | I | ACD39770 | *Pochonia chlamydosporia* RDC1 | Radicicol | TE (macrolactone closure) | ([Zhou et al., 2010](#_ENREF_52)) |
| 7 | II | BAD22832 | *Bipolaris oryzae* PKS1 | T4HN | TE/CLC (the second-ring cyclization) | ([Moriwaki et al., 2004](#_ENREF_32)) |
| 8 | II | BAA18956 | *Colletotrichum lagenaria* PKS1 | T4HN | TE/CLC (the second-ring cyclization) | ([Vagstad et al., 2012](#_ENREF_42)) |
| 9 | II | ABU63483 | *Elsinoe fawcettii* PKS1 | Elsinochrome | TE/CLC (the second-ring cyclization) | ([Liao and Chung, 2008](#_ENREF_27)) |
| 10 | II | AAO60166 | *Endoconidiophora resinifera* PKS1 | T4HN | TE/CLC (the second-ring cyclization) | ([Loppnau et al., 2004](#_ENREF_31)) |
| 11 | II | AAD31436 | *Exophiala dermatitidis* PKS1 | T4HN | TE/CLC (the second-ring cyclization) | ([Wheeler et al., 2008](#_ENREF_44)) |
| 12 | II | AAN75188 | *Exophiala lecanii-corni* PKS1 | T4HN | TE/CLC (the second-ring cyclization) | ([Cheng et al., 2004](#_ENREF_8)) |
| 13 | II | AAN59953 | *Glarea lozoyensis* PKS1 | T4HN | TE/CLC (the second-ring cyclization) | ([Zhang et al., 2003](#_ENREF_50)) |
| 14 | II | AAD38786 | *Nodulisporium* sp. PKS1 | T4HN | TE/CLC (the second-ring cyclization) | ([Fulton et al., 1999](#_ENREF_18)) |
| 15 | II | ABD47522 | *Ophiostoma piceae* PKSA | T4HN | TE/CLC (the second-ring cyclization) | ([Tanguay et al., 2007](#_ENREF_39)) |
| 16 | II | CAM35471 | *Sordaria macrospora* PKS | T4HN | TE/CLC (the second-ring cyclization) | ([Engh et al., 2007](#_ENREF_15)) |
| 17 | III | AAC39471 | *Aspergillus fumigatus* Alb1 | Naphthopyrones | TE/CLC (the second-ring cyclization) | ([Tsai et al., 2001](#_ENREF_41)) |
| 18 | III | EDP55264 | *Aspergillus fumigatus* PksP | T4HN | TE/CLC (the second-ring cyclization) | ([Langfelder et al., 1998](#_ENREF_25)) |
| 19 | III | Q03149 | *Aspergillus nidulans* WA | YWA1, Naphthopyrone | TE/CLC (the second-ring cyclization) | ([Fujii et al., 2001](#_ENREF_17)) |
| 20 | III | EHA28527 | *Aspergillus niger* AlbA | YWA1, Dimeric naphtho-γ-pyrones | TE/CLC (the second-ring cyclization) | ([Chiang et al., 2011](#_ENREF_9)) |
| 21 | III | CAB92399 | *Fusarium fujikuroi* PKS4 | Bikaverin | TE/CLC (the second-ring cyclization) | ([Linnemannstons et al., 2002](#_ENREF_29)) |
| 22 | III | AAU10633 | *Fusarium graminearum* PKS12 | Aurofusarin | TE/CLC (the second-ring cyclization) | ([Frandsen et al., 2011](#_ENREF_16)) |
| 23 | IV | AAS90093 | *Aspergillus flavus* PksA | Aflatoxin | TE/CLC (the third-ring cyclization) | ([Ehrlich et al., 2004](#_ENREF_14)) |
| 24 | IV | Q12397 | *Aspergillus nidulans* StcA | Sterigmatocystin | TE/CLC (the third-ring cyclization) | ([Klejnstrup et al., 2012](#_ENREF_21)) |
| 25 | IV | ACH72912 | *Aspergillus ochraceoroseus* AflC | Aflatoxin | TE/CLC (the third-ring cyclization) | ([Cary et al., 2009](#_ENREF_7)) |
| 26 | IV | BAE71314 | *Aspergillus oryzae* PKSA | Aflatoxin | TE/CLC (the third-ring cyclization) | ([Tominaga et al., 2006](#_ENREF_40)) |
| 27 | IV | AAS66004 | *Aspergillus parasiticus* AflC | Aflatoxin | TE/CLC (the third-ring cyclization) | ([Yu et al., 2004](#_ENREF_48)) |
| 28 | IV | Q12053 | *Aspergillus parasiticus* PKSA | Aflatoxin | TE/CLC (the third-ring cyclization) | ([Korman et al., 2010](#_ENREF_22)) |
| 29 | IV | AAT69682 | *Cercospora nicotianae* CTB1 | Cercosporin | TE (pyrone formation) | ([Newman et al., 2012](#_ENREF_33)) |
| 30 | IV | AAZ95017 | *Dothistroma septosporum* PKSA | Aflatoxin | TE/CLC (the third-ring cyclization) | ([Zhang et al., 2007](#_ENREF_51)) |
| 31 | IV | CCE67070 | *Fusarium fujikuroi* Fsr1 | Fusarubin | R | ([Studt et al., 2012](#_ENREF_37)) |
| 32 | IV | AAS92537 | *Leptosphaeria maculans* PKS1 | Sirodesmin PL | TE/CLC (the third-ring cyclization) | ([Gardiner et al., 2004](#_ENREF_19)) |
| 33 | IV | XP_003039929 | *Nectria haematococca* PKS1 | Bostrycoidin, Fusarubin | R | ([Awakawa et al., 2012](#_ENREF_2)) |
| 34 | V | XP_746434 | *Aspergillus fumigatus* EncB | Endocrocin | MβL-TE | ([Lim et al., 2012](#_ENREF_28)) |
| 35 | V | CBF70385 | *Aspergillus nidulans* AptB | Asperthecin | MβL-TE | ([Szewczyk et al., 2008](#_ENREF_38)) |
| 36 | V | CBF90099 | *Aspergillus nidulans* MdpF | Atrochrysone carboxylic acid | MβL-TE | ([Chiang et al., 2010](#_ENREF_10)) |
| 37 | V | CBF79145 | *Aspergillus nidulans* PkgB | Dehydrocitreoisocoumarin, Citreisocoumarin, Alternariol | MβL-TE | ([Ahuja et al., 2012](#_ENREF_1)) |
| 38 | V | XP_001394706 | *Aspergillus niger* AdaB | TAN-1612,BMS-192548 | MβL-TE | ([Li et al., 2011](#_ENREF_26)) |
| 39 | V | XP_001217071 | *Aspergillus terreus* ACTE | Emodin | MβL-TE | ([Awakawa et al., 2009](#_ENREF_3)) |
| 40 | V | ADI24932 | *Penicillium aethiopicum* VrtG | Viridicatumtoxin | MβL-TE | ([Chooi et al., 2010](#_ENREF_12)) |
| 41 | VI | XP_681652 | *Aspergillus nidulans* AusA | 3,5-dimethylorsellinic acid, Austinol, Dehydroaustinol | TE-like (hydrolysis) | ([Lo et al., 2012](#_ENREF_30)) |
| 42 | VI | XP_664052 | *Aspergillus nidulans* PkbA | 3-methylorsellinic acid, Cichorine | TE-like (hydrolysis) | ([Ahuja et al., 2012](#_ENREF_1)) |
| 43 | VI | ADY00130 | *Penicillium brevicompactum* MpaC | 5-methylorsellinic acid, Mycophenolic acid | TE-like (hydrolysis) | ([Regueira et al., 2011](#_ENREF_35)) |
| 44 | VI | est_GWPlus_C_190476 | *Aspergillus niger* DtbA | 2,4-dihydroxy-3,5,6-trimethylbenzaldehyde, 6-ethyl-2,4-dihydroxy-3,5-dimethylbenzaldehyde | R | ([Yeh et al., 2013](#_ENREF_47)) |
| 45 | VII | CAN87161 | *Acremonium strictum* MOS | 3-methylorcinaldehyde | R | ([Bailey et al., 2007](#_ENREF_4)) |
| 46 | VII | XP_658638 | *Aspergillus nidulans* AfoE | Asperfuranone | R | ([Chiang et al., 2009](#_ENREF_11)) |
| 47 | VII | XP_658127 | *Aspergillus nidulans* PkdA | 2-ethyl-4,6-dihydroxy-3,5-dimethylbenzaldehyde | R | ([Ahuja et al., 2012](#_ENREF_1)) |
| 48 | VII | ANID_07903 | *Aspergillus nidulans* PkeA | 2,4-dihydroxy-3-methyl-6-(2-oxopropyl)benzaldehyde | R | ([Ahuja et al., 2012](#_ENREF_1)) |
| 49 | VII | XP_660834 | *Aspergillus nidulans* PkfA | Orsellinaldehyde | R | ([Ahuja et al., 2012](#_ENREF_1)) |
| 50 | VII | XP_659636 | *Aspergillus nidulans* PkhA | 2,4-dihydroxy-6[(3E,5E,7E)-2-oxonona-3,5,7-trienyl]benzaldehyde | R | ([Ahuja et al., 2012](#_ENREF_1)) |
| 51 | VII | XP_660990 | *Aspergillus nidulans* PkiA | 2,4-dihydroxy-3-methyl-6-(2-oxopropyl)benzaldehyde | R | ([Ahuja et al., 2012](#_ENREF_1)) |
| 52 | VII | EHA28237 | *Aspergillus niger* AzaA | Azanigerone A | R | ([Zabala et al., 2012](#_ENREF_49)) |
| 53 | VII | XP_001212610 | *Aspergillus terreus* ATEG_03432 | Citrinin | R | ([Boruta and Bizukojc, 2014](#_ENREF_6)) |
| 54 | VII | AGN71604 | *Monascus pilosus* PKS5 | Rubropunctatin | R | ([Balakrishnan et al., 2013](#_ENREF_5)) |
| 55 | VII | BAD44749 | *Monascus purpureus* PksCT | Citrinin | R | ([Shimizu et al., 2005](#_ENREF_36)) |
| 56 | VII | DAA64703 | *Talaromyces stipitatus* TropA | 3-methylorcinaldehyde | R | ([Davison et al., 2012](#_ENREF_13)) |
| 57 | VIII | AFL91703 | *Armillaria mellea* ArmB | Orsellinic acid, melledonol | TE (cross-coupling) | ([Lackner et al., 2013](#_ENREF_23)) |
| 58 | VIII | XP_007307184 | *Stereum hirsutum* FP-91666 SS1 PKS1 | MS3 | TE (cross-coupling) | ([Lackner et al., 2012](#_ENREF_24)) |

The related information of group V and *Aspergillus niger* DtbA in groupVI is showed by physically discrete PRE domains instead of NR-PKSs.

References

Ahuja, M., Chiang, Y.M., Chang, S.L., Praseuth, M.B., Entwistle, R., Sanchez, J.F., Lo, H.C., Yeh, H.H., Oakley, B.R., and Wang, C.C. (2012). Illuminating the diversity of aromatic polyketide synthases in *Aspergillus nidulans*. *J Am Chem Soc* 134**,** 8212-8221.

Awakawa, T., Kaji, T., Wakimoto, T., and Abe, I. (2012). A heptaketide naphthaldehyde produced by a polyketide synthase from *Nectria haematococca*. *Bioorg Med Chem Lett* 22**,** 4338-4340.

Awakawa, T., Yokota, K., Funa, N., Doi, F., Mori, N., Watanabe, H., and Horinouchi, S. (2009). Physically discrete beta-lactamase-type thioesterase catalyzes product release in atrochrysone synthesis by iterative type I polyketide synthase. *Chem Biol* 16**,** 613-623.

Bailey, A.M., Cox, R.J., Harley, K., Lazarus, C.M., Simpson, T.J., and Skellam, E. (2007). Characterisation of 3-methylorcinaldehyde synthase (MOS) in *Acremonium strictum*: first observation of a reductive release mechanism during polyketide biosynthesis. *Chem Commun (Camb)***,** 4053-4055.

Balakrishnan, B., Karki, S., Chiu, S.H., Kim, H.J., Suh, J.W., Nam, B., Yoon, Y.M., Chen, C.C., and Kwon, H.J. (2013). Genetic localization and in vivo characterization of a *Monascus azaphilone* pigment biosynthetic gene cluster. *Appl Microbiol Biotechnol* 97**,** 6337-6345.

Boruta, T., and Bizukojc, M. (2014). Culture-based and sequence-based insights into biosynthesis of secondary metabolites by *Aspergillus terreus* ATCC 20542. *J Biotechnol* 175**,** 53-62.

Cary, J.W., Ehrlich, K.C., Beltz, S.B., Harris-Coward, P., and Klich, M.A. (2009). Characterization of the *Aspergillus ochraceoroseus* aflatoxin/sterigmatocystin biosynthetic gene cluster. *Mycologia* 101**,** 352-362.

Cheng, Q., Kinney, K.A., Whitman, C.P., and Szaniszlo, P.J. (2004). Characterization of two polyketide synthase genes in *Exophiala lecanii-corni*, a melanized fungus with bioremediation potential. *Bioorg Chem* 32**,** 92-108.

Chiang, Y.M., Meyer, K.M., Praseuth, M., Baker, S.E., Bruno, K.S., and Wang, C.C. (2011). Characterization of a polyketide synthase in *Aspergillus niger* whose product is a precursor for both dihydroxynaphthalene (DHN) melanin and naphtho-gamma-pyrone. *Fungal Genet Biol* 48**,** 430-437.

Chiang, Y.M., Szewczyk, E., Davidson, A.D., Entwistle, R., Keller, N.P., Wang, C.C., and Oakley, B.R. (2010). Characterization of the *Aspergillus nidulans* monodictyphenone gene cluster. *Appl Environ Microbiol* 76**,** 2067-2074.

Chiang, Y.M., Szewczyk, E., Davidson, A.D., Keller, N., Oakley, B.R., and Wang, C.C. (2009). A gene cluster containing two fungal polyketide synthases encodes the biosynthetic pathway for a polyketide, asperfuranone, in *Aspergillus nidulans*. *J Am Chem Soc* 131**,** 2965-2970.

Chooi, Y.H., Cacho, R., and Tang, Y. (2010). Identification of the viridicatumtoxin and griseofulvin gene clusters from *Penicillium aethiopicum*. *Chem Biol* 17**,** 483-494.

Davison, J., Al Fahad, A., Cai, M., Song, Z., Yehia, S.Y., Lazarus, C.M., Bailey, A.M., Simpson, T.J., and Cox, R.J. (2012). Genetic, molecular, and biochemical basis of fungal tropolone biosynthesis. *Proc Natl Acad Sci U S A* 109**,** 7642-7647.

Ehrlich, K.C., Chang, P.K., Yu, J., and Cotty, P.J. (2004). Aflatoxin biosynthesis cluster gene cypA is required for G aflatoxin formation. *Appl Environ Microbiol* 70**,** 6518-6524.

Engh, I., Nowrousian, M., and Kuck, U. (2007). Regulation of melanin biosynthesis via the dihydroxynaphthalene pathway is dependent on sexual development in the ascomycete *Sordaria macrospora*. *FEMS Microbiol Lett* 275**,** 62-70.

Frandsen, R.J., Schutt, C., Lund, B.W., Staerk, D., Nielsen, J., Olsson, S., and Giese, H. (2011). Two novel classes of enzymes are required for the biosynthesis of aurofusarin in *Fusarium graminearum*. *J Biol Chem* 286**,** 10419-10428.

Fujii, I., Watanabe, A., Sankawa, U., and Ebizuka, Y. (2001). Identification of Claisen cyclase domain in fungal polyketide synthase WA, a naphthopyrone synthase of *Aspergillus nidulans*. *Chem Biol* 8**,** 189-197.

Fulton, T.R., Ibrahim, N., Losada, M.C., Grzegorski, D., and Tkacz, J.S. (1999). A melanin polyketide synthase (PKS) gene from Nodulisporium sp. that shows homology to the pks1 gene of *Colletotrichum lagenarium*. *Mol Gen Genet* 262**,** 714-720.

Gardiner, D.M., Cozijnsen, A.J., Wilson, L.M., Pedras, M.S., and Howlett, B.J. (2004). The sirodesmin biosynthetic gene cluster of the plant pathogenic fungus *Leptosphaeria maculans*. *Mol Microbiol* 53**,** 1307-1318.

Gressler, M., Hortschansky, P., Geib, E., and Brock, M. (2015). A new high-performance heterologous fungal expression system based on regulatory elements from the *Aspergillus terreus* terrein gene cluster. *Front Microbiol* 6**,** 184.

Klejnstrup, M.L., Frandsen, R.J., Holm, D.K., Nielsen, M.T., Mortensen, U.H., Larsen, T.O., and Nielsen, J.B. (2012). Genetics of Polyketide Metabolism in *Aspergillus nidulans*. *Metabolites* 2**,** 100-133.

Korman, T.P., Crawford, J.M., Labonte, J.W., Newman, A.G., Wong, J., Townsend, C.A., and Tsai, S.C. (2010). Structure and function of an iterative polyketide synthase thioesterase domain catalyzing Claisen cyclization in aflatoxin biosynthesis. *Proc Natl Acad Sci U S A* 107**,** 6246-6251.

Lackner, G., Bohnert, M., Wick, J., and Hoffmeister, D. (2013). Assembly of melleolide antibiotics involves a polyketide synthase with cross-coupling activity. *Chem Biol* 20**,** 1101-1106.

Lackner, G., Misiek, M., Braesel, J., and Hoffmeister, D. (2012). Genome mining reveals the evolutionary origin and biosynthetic potential of basidiomycete polyketide synthases. *Fungal Genet Biol* 49**,** 996-1003.

Langfelder, K., Jahn, B., Gehringer, H., Schmidt, A., Wanner, G., and Brakhage, A.A. (1998). Identification of a polyketide synthase gene (pksP) of *Aspergillus fumigatus* involved in conidial pigment biosynthesis and virulence. *Med Microbiol Immunol* 187**,** 79-89.

Li, Y., Chooi, Y.H., Sheng, Y., Valentine, J.S., and Tang, Y. (2011). Comparative characterization of fungal anthracenone and naphthacenedione biosynthetic pathways reveals an alpha-hydroxylation-dependent Claisen-like cyclization catalyzed by a dimanganese thioesterase. *J Am Chem Soc* 133**,** 15773-15785.

Liao, H.L., and Chung, K.R. (2008). Genetic dissection defines the roles of elsinochrome Phytotoxin for fungal pathogenesis and conidiation of the citrus pathogen *Elsinoe fawcettii*. *Mol Plant Microbe Interact* 21**,** 469-479.

Lim, F.Y., Hou, Y., Chen, Y., Oh, J.H., Lee, I., Bugni, T.S., and Keller, N.P. (2012). Genome-based cluster deletion reveals an endocrocin biosynthetic pathway in *Aspergillus fumigatus*. *Appl Environ Microbiol* 78**,** 4117-4125.

Linnemannstons, P., Schulte, J., Del Mar Prado, M., Proctor, R.H., Avalos, J., and Tudzynski, B. (2002). The polyketide synthase gene pks4 from *Gibberella fujikuroi* encodes a key enzyme in the biosynthesis of the red pigment bikaverin. *Fungal Genet Biol* 37**,** 134-148.

Lo, H.C., Entwistle, R., Guo, C.J., Ahuja, M., Szewczyk, E., Hung, J.H., Chiang, Y.M., Oakley, B.R., and Wang, C.C. (2012). Two separate gene clusters encode the biosynthetic pathway for the meroterpenoids austinol and dehydroaustinol in *Aspergillus nidulans*. *J Am Chem Soc* 134**,** 4709-4720.

Loppnau, P., Tanguay, P., and Breuil, C. (2004). Isolation and disruption of the melanin pathway polyketide synthase gene of the softwood deep stain fungus *Ceratocystis resinifera*. *Fungal Genetics and Biology* 41**,** 33-41.

Moriwaki, A., Kihara, J., Kobayashi, T., Tokunaga, T., Arase, S., and Honda, Y. (2004). Insertional mutagenesis and characterization of a polyketide synthase gene (PKS1) required for melanin biosynthesis in *Bipolaris oryzae*. *FEMS Microbiol Lett* 238**,** 1-8.

Newman, A.G., Vagstad, A.L., Belecki, K., Scheerer, J.R., and Townsend, C.A. (2012). Analysis of the cercosporin polyketide synthase CTB1 reveals a new fungal thioesterase function. *Chem Commun (Camb)* 48**,** 11772-11774.

Reeves, C.D., Hu, Z., Reid, R., and Kealey, J.T. (2008). Genes for the biosynthesis of the fungal polyketides hypothemycin from *Hypomyces subiculosus* and radicicol from *Pochonia chlamydosporia*. *Appl Environ Microbiol* 74**,** 5121-5129.

Regueira, T.B., Kildegaard, K.R., Hansen, B.G., Mortensen, U.H., Hertweck, C., and Nielsen, J. (2011). Molecular basis for mycophenolic acid biosynthesis in *Penicillium brevicompactum*. *Appl Environ Microbiol* 77**,** 3035-3043.

Shimizu, T., Kinoshita, H., Ishihara, S., Sakai, K., Nagai, S., and Nihira, T. (2005). Polyketide synthase gene responsible for citrinin biosynthesis in *Monascus purpureus*. *Appl Environ Microbiol* 71**,** 3453-3457.

Studt, L., Wiemann, P., Kleigrewe, K., Humpf, H.U., and Tudzynski, B. (2012). Biosynthesis of fusarubins accounts for pigmentation of *Fusarium fujikuroi* perithecia. *Appl Environ Microbiol* 78**,** 4468-4480.

Szewczyk, E., Chiang, Y.M., Oakley, C.E., Davidson, A.D., Wang, C.C., and Oakley, B.R. (2008). Identification and characterization of the asperthecin gene cluster of *Aspergillus nidulans*. *Appl Environ Microbiol* 74**,** 7607-7612.

Tanguay, P., Tangen, K., and Breuil, C. (2007). Identifying Pigmentation-Related Genes in *Ophiostoma piceae* Using Agrobacterium-Mediated Integration. *Phytopathology* 97**,** 1040-1048.

Tominaga, M., Lee, Y.H., Hayashi, R., Suzuki, Y., Yamada, O., Sakamoto, K., Gotoh, K., and Akita, O. (2006). Molecular analysis of an inactive aflatoxin biosynthesis gene cluster in *Aspergillus oryzae* RIB strains. *Appl Environ Microbiol* 72**,** 484-490.

Tsai, H.F., Fujii, I., Watanabe, A., Wheeler, M.H., Chang, Y.C., Yasuoka, Y., Ebizuka, Y., and Kwon-Chung, K.J. (2001). Pentaketide melanin biosynthesis in *Aspergillus fumigatus* requires chain-length shortening of a heptaketide precursor. *J Biol Chem* 276**,** 29292-29298.

Vagstad, A.L., Hill, E.A., Labonte, J.W., and Townsend, C.A. (2012). Characterization of a fungal thioesterase having Claisen cyclase and deacetylase activities in melanin biosynthesis. *Chem Biol* 19**,** 1525-1534.

Wang, M., Zhou, H., Wirz, M., Tang, Y., and Boddy, C.N. (2009). A thioesterase from an iterative fungal polyketide synthase shows macrocyclization and cross coupling activity and may play a role in controlling iterative cycling through product offloading. *Biochemistry* 48**,** 6288-6290.

Wheeler, M.H., Abramczyk, D., Puckhaber, L.S., Naruse, M., Ebizuka, Y., Fujii, I., and Szaniszlo, P.J. (2008). New biosynthetic step in the melanin pathway of *Wangiella (Exophiala) dermatitidis*: evidence for 2-acetyl-1,3,6,8-Tetrahydroxynaphthalene as a novel precursor. *Eukaryot Cell* 7**,** 1699-1711.

Xu, Y., Espinosa-Artiles, P., Schubert, V., Xu, Y.M., Zhang, W., Lin, M., Gunatilaka, A.A., Sussmuth, R., and Molnar, I. (2013a). Characterization of the biosynthetic genes for 10,11-dehydrocurvularin, a heat shock response-modulating anticancer fungal polyketide from *Aspergillus terreus*. *Appl Environ Microbiol* 79**,** 2038-2047.

Xu, Y., Zhou, T., Zhang, S., Xuan, L.J., Zhan, J., and Molnar, I. (2013b). Thioesterase domains of fungal nonreducing polyketide synthases act as decision gates during combinatorial biosynthesis. *J Am Chem Soc* 135**,** 10783-10791.

Yeh, H.H., Chang, S.L., Chiang, Y.M., Bruno, K.S., Oakley, B.R., Wu, T.K., and Wang, C.C. (2013). Engineering fungal nonreducing polyketide synthase by heterologous expression and domain swapping. *Org Lett* 15**,** 756-759.

Yu, J., Chang, P.K., Ehrlich, K.C., Cary, J.W., Bhatnagar, D., Cleveland, T.E., Payne, G.A., Linz, J.E., Woloshuk, C.P., and Bennett, J.W. (2004). Clustered Pathway Genes in Aflatoxin Biosynthesis. *Applied and Environmental Microbiology* 70**,** 1253-1262.

Zabala, A.O., Xu, W., Chooi, Y.H., and Tang, Y. (2012). Characterization of a silent azaphilone gene cluster from *Aspergillus niger* ATCC 1015 reveals a hydroxylation-mediated pyran-ring formation. *Chem Biol* 19**,** 1049-1059.

Zhang, A., Lu, P., Dahl-Roshak, A.M., Paress, P.S., Kennedy, S., Tkacz, J.S., and An, Z. (2003). Efficient disruption of a polyketide synthase gene ( pks1) required for melanin synthesis through Agrobacterium-mediated transformation of *Glarea lozoyensis*. *Mol Genet Genomics* 268**,** 645-655.

Zhang, S., Schwelm, A., Jin, H., Collins, L.J., and Bradshaw, R.E. (2007). A fragmented aflatoxin-like gene cluster in the forest pathogen *Dothistroma septosporum*. *Fungal Genet Biol* 44**,** 1342-1354.

Zhou, H., Qiao, K., Gao, Z., Vederas, J.C., and Tang, Y. (2010). Insights into radicicol biosynthesis via heterologous synthesis of intermediates and analogs. *J Biol Chem* 285**,** 41412-41421.
