# Supplementary material for: Analysis of the Sequences, Structures, and Functions of Product-Releasing Enzyme Domains in Fungal Polyketide Synthases
Source: Front Microbiol. 2017 Sep 4;8:1685. doi: 10.3389/fmicb.2017.01685 (PMC5591372; doi:10.3389/fmicb.2017.01685)
Supplement: Supplementary file 5 [file Table_3.DOCX]

**Table S3. The structural templates for the comparative modeling and quality validation.**

| **Accession No.** | **Ramachandran plot (%)** | | | | **Template** | **Seq Similarity** |
| --- | --- | --- | --- | --- | --- | --- |
|  | **core** | **allow** | **gener** | **disall** |  |  |
| ABB90282 | 85.4 | 12.0 | 1.3 | 1.3 | 3ils.1.A | 0.34 |
| ACD39762 | 87.7 | 9.8 | 2.0 | 0.5 | 3ils.1.A | 0.36 |
| ACD39770 | 81.6 | 13.1 | 3.7 | 1.6 | 3ils.1.A | 0.34 |
| ACM42403 | 83.5 | 11.3 | 2.6 | 2.6 | 3ils.1.A | 0.33 |
| AGC95321 | 83.6 | 11.3 | 2.9 | 2.1 | 3ils.1.A | 0.35 |
| XP_681178 | 83.3 | 13.2 | 2.2 | 1.3 | 3ils.1.A | 0.34 |
| AAD31436 | 88.4 | 8.2 | 1.4 | 1.9 | 3ils.1.A | 0.41 |
| AAD38786 | 85.4 | 11.3 | 1.4 | 1.9 | 3ils.1.A | 0.39 |
| AAN59953 | 85.1 | 13.0 | 0.5 | 1.4 | 3ils.1.A | 0.41 |
| AAN75188 | 87.0 | 12.1 | 0.5 | 0.5 | 3ils.1.A | 0.40 |
| AAO60166 | 86.7 | 10.6 | 0.9 | 1.8 | 3ils.1.A | 0.40 |
| ABD47522 | 86.5 | 10.7 | 0.9 | 1.9 | 3ils.1.A | 0.38 |
| ABU63483 | 85.2 | 13.8 | 0.5 | 0.5 | 3ils.1.A | 0.40 |
| BAA18956 | 86.1 | 11.0 | 1.4 | 1.4 | 3ils.1.A | 0.40 |
| BAD22832 | 87.7 | 10.8 | 1.0 | 0.5 | 3ils.1.A | 0.41 |
| CAM35471 | 86.0 | 11.6 | 0.9 | 1.4 | 3ils.1.A | 0.41 |
| AAC39471 | 84.9 | 13.2 | 0.9 | 0.9 | 3ils.1.A | 0.43 |
| AAU10633 | 87.5 | 9.5 | 1.7 | 1.3 | 3ils.1.A | 0.40 |
| CAB92399 | 88.4 | 9.7 | 1.4 | 0.5 | 3ils.1.A | 0.41 |
| EDP55264 | 84.9 | 13.2 | 0.9 | 0.9 | 3ils.1.A | 0.43 |
| EHA28527 | 85.1 | 13.0 | 0.9 | 0.9 | 3ils.1.A | 0.43 |
| Q03149 | 85.7 | 12.4 | 1.4 | 0.5 | 3ils.1.A | 0.43 |
| AAS66004 | 89.1 | 10.0 | 0.5 | 0.5 | 3ils.1.A | 0.62 |
| AAS90093 | 89.0 | 10.0 | 0.5 | 0.5 | 3ils.1.A | 0.62 |
| AAS92537 | 90.1 | 8.9 | 0.5 | 0.5 | 3ils.1.A | 0.53 |
| AAT69682 | 89.3 | 7.6 | 1.8 | 1.3 | 3ils.1.A | 0.39 |
| AAZ95017 | 86.9 | 11.7 | 0.9 | 0.5 | 3ils.1.A | 0.51 |
| ACH72912 | 88.8 | 10.2 | 0.5 | 0.5 | 3ils.1.A | 0.56 |
| CCE67070 | 84.9 | 12.4 | 1.5 | 1.2 | 4u7w.1.A | 0.32 |
| Q12053 | 90.9 | 8.2 | 0.5 | 0.5 | - | - |
| Q12397 | 89.4 | 9.6 | 0.5 | 0.5 | 3ils.1.A | 0.55 |
| XP_003039929 | 84.5 | 12.4 | 0.9 | 2.1 | 4u7w.1.A | 0.31 |
| ADI24932 | 87.7 | 9.8 | 1.3 | 1.3 | 4ad9.1.A | 0.39 |
| CBF70385 | 88.7 | 8.8 | 0.8 | 1.7 | 4ad9.1.A | 0.39 |
| CBF79145 | 88.7 | 8.2 | 2.0 | 1.2 | 4ad9.1.A | 0.39 |
| CBF90099 | 83.7 | 14.1 | 1.8 | 0.4 | 4ad9.1.A | 0.41 |
| XP_746434 | 85.2 | 10.7 | 2.5 | 1.6 | 4ad9.1.A | 0.39 |
| XP_001217071 | 89.8 | 8.6 | 0.4 | 1.2 | 4ad9.1.A | 0.40 |
| XP_001394706 | 88.8 | 9.2 | 1.3 | 0.8 | 4ad9.1.A | 0.39 |
| ADY00130 | 78.5 | 14.7 | 4.4 | 2.4 | 2yh2.1.A | 0.30 |
| XP_664052 | 82.5 | 14.4 | 1.7 | 1.4 | 2yh2.1.A | 0.31 |
| XP_681652 | 79.1 | 15.2 | 3.4 | 2.4 | 2yh2.1.A | 0.30 |
| AGN71604 | 85.9 | 10.7 | 1.2 | 2.1 | 4u7w.1.A | 0.32 |
| ANID_07903 | 83.8 | 12.4 | 1.5 | 2.4 | 4u7w.1.A | 0.33 |
| BAD44749 | 82.4 | 12.7 | 3.3 | 1.5 | 4u7w.1.A | 0.32 |
| CAN87161 | 83.6 | 13.3 | 1.2 | 1.8 | 4u7w.1.A | 0.32 |
| DAA64703 | 82.1 | 13.0 | 2.5 | 2.5 | 4u7w.1.A | 0.31 |
| EHA28237 | 82.8 | 13.6 | 1.8 | 1.8 | 4u7w.1.A | 0.32 |
| est_GWPlus_C_190476 | 84.1 | 13.3 | 1.6 | 1.0 | 4u7w.1.A | 0.33 |
| XP_658127 | 85.5 | 11.8 | 1.5 | 1.2 | 4u7w.1.A | 0.31 |
| XP_658638 | 85.9 | 11.1 | 1.8 | 1.2 | 4u7w.1.A | 0.32 |
| XP_659636 | 83.7 | 12.4 | 2.4 | 1.5 | 4u7w.1.A | 0.34 |
| XP_660834 | 82.2 | 13.9 | 2.4 | 1.5 | 4u7w.1.A | 0.32 |
| XP_660990 | 86.4 | 11.4 | 0.6 | 1.6 | 4u7w.1.A | 0.31 |
| XP_001212610 | 83.1 | 13.1 | 2.1 | 1.8 | 4u7w.1.A | 0.33 |
| XP_007307184 | 83.1 | 12.3 | 2.1 | 2.6 | 3ils.1.A | 0.31 |
| AFL91703 | 79.8 | 14.4 | 3.7 | 2.1 | 3ils.1.A | 0.30 |
