# Supplementary material for: Analysis of the Sequences, Structures, and Functions of Product-Releasing Enzyme Domains in Fungal Polyketide Synthases
Source: Front Microbiol. 2017 Sep 4;8:1685. doi: 10.3389/fmicb.2017.01685 (PMC5591372; doi:10.3389/fmicb.2017.01685)
Supplement: Supplementary file 6 [file Table_4.DOCX]

**Table S4.** The lid-loop sequence lengths and synthesized polyketide chain lengths of TE domains of NR-PKSs.

| **Group** | **Accession No.** | **Sequence lengths of TE lid-loops** | **Chain lengths of polyketide intermediates^*^** | **Structures of final products** |
| --- | --- | --- | --- | --- |
| I | ABB90282 | 18 | 18 |   Zearalenone |
| I | ACD39762 | 18 | 18 |   Hypothemycin |
| I | ACD39770 | 20 | 18 |   Radicicol |
| I | ACM42403 | 20 | 18 |   Radicicol |
| I | AGC95321 | 18 | 16 |   10,11-Dehydrocurvularin |
| I | XP_681178 | 15 | 8 |   Lecanoric acid |
| II | AAD31436 | 13 | 12 |   T4HN |
| II | AAD38786 | 16 | 12 |   T4HN |
| II | AAN59953 | 14 | 12 |   T4HN |
| II | AAN75188 | 13 | 12 |   T4HN |
| II | AAO60166 | 18 | 12 |   T4HN |
| II | ABD47522 | 14 | 12 |   T4HN |
| II | ABU63483 | 14 | 12 |   Elsinochrome |
| II | BAA18956 | 17 | 12 |   T4HN |
| II | BAD22832 | 14 | 12 |   T4HN |
| II | CAM35471 | 15 | 12 |   T4HN |
| III | AAC39471 | 13 | 14 |   YWA1 |
| III | AAU10633 | 21 | 14 | Aurofusarin |
| III | CAB92399 | 18 | 18 |   Bikaverin |
| III | EDP55264 | 13 | 14 |   T4HN |
| III | EHA28527 | 13 | 14 |   YWA1 |
| III | Q03149 | 13 | 14 |   YWA1 |
| IV | AAS66004 | 20 | 20 |   Aflatoxin |
| IV | AAS90093 | 20 | 20 |   Aflatoxin |
| IV | AAT69682 | 20 | 14 |   Cercosporin |
| IV | ACH72912 | 20 | 20 |   Aflatoxin |
| IV | Q12053 | 20 | 20 |   Aflatoxin |
| IV | Q12397 | 20 | 20 |   Sterigmatocystin |
| VIII | XP_007307184 | 10 | 8 |   MS3 |
| VIII | AFL91703 | 14 | 8 |   Orsellinic acid |

^*^The numbers of carbon atoms were used to represent the chain length of polyketide intermediates.
