# Supplementary material for: Analysis of the Sequences, Structures, and Functions of Product-Releasing Enzyme Domains in Fungal Polyketide Synthases
Source: Front Microbiol. 2017 Sep 4;8:1685. doi: 10.3389/fmicb.2017.01685 (PMC5591372; doi:10.3389/fmicb.2017.01685)
Supplement: Supplementary file 7 [file Table_5.DOCX]

**Table S5. Conservation analysis of residue sites in TE domains.**

| **Position** | | **Group I** | | | **Group II** | | | **Group III** | | | **Group IV** | | | **Group VIII** | | |
| --- | --- | --- | --- | --- | --- | --- | --- | --- | --- | --- | --- | --- | --- | --- | --- | --- |
|  |  | **XP_681178** | **MAX*** | **Grade**** | **BAA18956** | **MAX** | **Grade** | **Q03149** | **MAX** | **Grade** | **Q12053** | **MAX** | **Grade** | **AFL91703** | **MAX** | **Grade** |
| 1 | | Q1823 | E 20 | 1 | D1917 | D 38 | 6 | S1875 | V 23 | 1 | L1845 | L 27 | 1 | V1954 | A 23 | 1 |
| 2 | | T1824 | T 36 | 3 | N1918 | N 22 | 1 | S1876 | V 35 | 1 | K1846 | K 24 | 1 | R1955 | A 25 | 2 |
| 3 | | S1825 | P 47 | 2 | Y1919 | Y 47 | 1 | D1877 | A 30 | 1 | P1847 | P 29 | 1 | V1956 | I 20 | 1 |
| 4 | | T1826 | K 29 | 1 | P1920 | P 96 | 9 | N1878 | E 19 | 1 | Y1848 | Y 20 | 1 | L1957 | L 52 | 3 |
| 5 | | N1827 | N 14 | 1 | H1921 | H 54 | 5 | H1879 | H 30 | 1 | C1849 | C 41 | 4 | R1958 | R 22 | 1 |
| 6 | | P1828 | K 21 | 1 | R1922 | R 96 | 8 | P1880 | P 84 | 8 | R1850 | R 43 | 6 | L1959 | L 68 | 6 |
| 7 | | A1829 | V 29 | 1 | K1923 | P 37 | 2 | P1881 | R 32 | 4 | P1851 | P 78 | 4 | D1960 | D 37 | 5 |
| 8 | | K1830 | A 43 | 6 | A1924 | A 90 | 8 | A1882 | A 75 | 9 | S1852 | A 56 | 5 | E1961 | E 21 | 1 |
| 9 | | E1831 | P 77 | 6 | T1925 | T 62 | 6 | T1883 | T 88 | 8 | T1853 | T 62 | 4 | V1962 | N 19 | 2 |
| 10 | | T1832 | Q 23 | 1 | S1926 | S 98 | 9 | S1884 | S 99 | 9 | S1854 | S 100 | 9 | P1963 | P 86 | 8 |
| 11 | | T1833 | P 24 | 1 | I1927 | V 66 | 7 | I1885 | I 53 | 7 | V1855 | V 65 | 8 | M1964 | V 30 | 5 |
| 12 | | I1834 | P 27 | 1 | L1928 | L 99 | 9 | L1886 | L 75 | 7 | V1856 | L 27 | 5 | S1965 | L 30 | 3 |
| 13 | | D1835 | T 29 | 1 | L1929 | L 100 | 9 | L1887 | L 96 | 9 | L1857 | L 94 | 7 | V1966 | L 39 | 7 |
| 14 | | S1836 | A 18 | 1 | Q1930 | Q 85 | 8 | Q1888 | Q 79 | 8 | Q1858 | Q 100 | 9 | Q1967 | Q 66 | 8 |
| 15 | | S1837 | T 24 | 1 | G1931 | G 100 | 9 | G1889 | G 93 | 8 | G1859 | G 90 | 6 | - | - | - |
| 16 | | R1838 | S 26 | 1 | S1932 | N 61 | 7 | N1890 | N 43 | 5 | L1860 | L 25 | 4 | K1968 | S 19 | 1 |
| 17 | | Q1839 | D 24 | 1 | T1933 | P 42 | 3 | P1891 | P 79 | 5 | P1861 | P 55 | 6 | S1969 | S 30 | 3 |
| 18 | | H1840 | D 24 | 1 | R1934 | K 58 | 6 | R1892 | K 55 | 2 | M1862 | K 27 | 2 | S1970 | S 18 | 1 |
| 19 | | K1841 | P 19 | 1 | T1935 | T 67 | 6 | T1893 | T 56 | 6 | V1863 | V 24 | 1 | S1971 | P 25 | 3 |
| 20 | | - | - | - | A1936 | A 97 | 9 | A1894 | A 75 | 9 | A1864 | A 67 | 7 | S1972 | G 23 | 1 |
| 21 | | - | - | - | T1937 | T 73 | 6 | S1895 | T 36 | 3 | R1865 | R 31 | 1 | G1973 | R 23 | 1 |
| 22 | | - | - | - | K1938 | K 73 | 6 | K1896 | K 32 | 1 | K1866 | K 63 | 5 | S1974 | A 30 | 3 |
| 23 | | - | - | - | N1939 | K 30 | 3 | T1897 | T 55 | 4 | T1867 | T 65 | 6 | P1975 | P 93 | 9 |
| 24 | | - | - | - | L1940 | L 73 | 6 | L1898 | L 84 | 6 | L1868 | L 100 | 9 | L1976 | L 87 | 8 |
| 25 | | - | - | - | W1941 | F 76 | 8 | F1899 | F 88 | 8 | F1869 | F 98 | 8 | F1977 | F 61 | 7 |
| 26 | | - | - | - | M1942 | L 45 | 7 | L1900 | L 91 | 8 | M1870 | L 71 | 6 | L1978 | L 91 | 9 |
| 27 | | - | - | - | V1943 | V 47 | 5 | F1901 | F 84 | 8 | L1871 | L 49 | 8 | F1979 | I 57 | 8 |
| 28 | | L1842 | S 31 | 3 | P1944 | P 100 | 9 | P1902 | P 100 | 9 | P1872 | P 100 | 9 | H1980 | H 100 | 9 |
| 29 | | D1843 | D 100 | 9 | D1945 | D 100 | 9 | D1903 | D 99 | 9 | D1873 | D 100 | 9 | D1981 | D 98 | 9 |
| 30 | | A1844 | G 95 | 8 | G1946 | G 100 | 9 | G1904 | G 100 | 9 | G1874 | G 100 | 9 | G1982 | G 100 | 9 |
| 31 | | A1845 | T 41 | 7 | S1947 | S 87 | 8 | S1905 | S 81 | 9 | G1875 | S 49 | 8 | S1983 | S 89 | 9 |
| 32 | | V1846 | G 97 | 9 | G1948 | G 100 | 9 | G1906 | G 100 | 9 | G1876 | G 100 | 9 | G1984 | G 100 | 9 |
| 33 | | S1847 | S 59 | 8 | C1949 | S 76 | 8 | S1907 | S 97 | 9 | S1877 | S 71 | 8 | A1985 | L 51 | 5 |
| 34 | | R1848 | I 38 | 7 | A1950 | A 96 | 9 | A1908 | A 89 | 8 | A1878 | A 86 | 8 | V1986 | I 36 | 5 |
| 35 | | A1849 | A 56 | 6 | T1951 | T 90 | 9 | T1909 | T 63 | 7 | F1879 | T 45 | 7 | N1987 | H 20 | 5 |
| 36 | | S1850 | T 41 | 7 | S1952 | S 100 | 9 | S1910 | S 100 | 9 | S1880 | S 100 | 9 | Y1988 | Y 36 | 3 |
| 37 | | Y1851 | Y 92 | 8 | Y1953 | Y 100 | 9 | Y1911 | Y 100 | 9 | Y1881 | Y 100 | 9 | L1989 | Y 82 | 8 |
| 38 | | I1852 | I 67 | 7 | T1954 | T 45 | 6 | A1912 | A 60 | 6 | A1882 | A 29 | 1 | R1990 | R 16 | 1 |
| 39 | | H1853 | H 82 | 8 | E1955 | E 46 | 6 | T1913 | T 47 | 5 | S1883 | S 31 | 1 | R1991 | R 62 | 7 |
| 40 | | L1854 | L 90 | 9 | I1956 | I 87 | 8 | I1914 | L 59 | 7 | L1884 | L 57 | 6 | L1992 | L 84 | 8 |
| 41 | | K1855 | P 64 | 6 | S1957 | P 53 | 6 | P1915 | P 72 | 7 | P1885 | P 98 | 9 | G1993 | S 22 | 3 |
| 42 | | A1856 | A 28 | 2 | Q1958 | N 32 | 1 | G1916 | G 17 | 2 | R1886 | R 62 | 5 | S1994 | P 56 | 4 |
| 43 | | L1857 | L 51 | 6 | V1959 | I 56 | 4 | V1917 | I 52 | 5 | L1887 | L 67 | 6 | V1995 | L 58 | 7 |
| 44 | | P1858 | K 26 | 3 | S1960 | S 51 | 6 | S1918 | S 61 | 5 | K1888 | S 29 | 1 | G1996 | G 49 | 5 |
| 45 | | K1859 | S 36 | 5 | S1961 | P 40 | 1 | P1919 | P 52 | 2 | S1889 | P 38 | 1 | R1997 | R 89 | 9 |
| 46 | | G1860 | G 41 | 5 | N1962 | D 52 | 4 | N1920 | D 75 | 4 | D1890 | D 50 | 5 | - | - | - |
| 47 | | R1861 | R 33 | 4 | W1963 | W 28 | 5 | V1921 | V 63 | 7 | T1891 | T 37 | 3 | - | - | - |
| 48 | | R1862 | P 49 | 5 | A1964 | A 88 | 7 | A1922 | A 39 | 7 | A1892 | A 81 | 8 | E1998 | D 33 | 4 |
| 49 | | I1863 | V 44 | 6 | V1965 | V 89 | 8 | V1923 | V 88 | 8 | V1893 | V 60 | 6 | F1999 | V 64 | 7 |
| 50 | | Y1864 | Y 95 | 8 | W1966 | W 46 | 4 | Y1924 | Y 77 | 8 | V1894 | V 73 | 8 | W2000 | W 51 | 6 |
| 51 | | A1865 | G 51 | 8 | G1967 | G 99 | 9 | G1925 | G 95 | 9 | G1895 | G 73 | 7 | G2001 | G 51 | 7 |
| 52 | | L1866 | I 36 | 7 | L1968 | L 92 | 8 | L1926 | L 97 | 9 | L1896 | L 63 | 6 | F2002 | I 69 | 8 |
| 53 | | E1867 | E 51 | 8 | F1969 | N 58 | 9 | N1927 | N 93 | 9 | N1897 | N 92 | 8 | N2003 | H 43 | 5 |
| 54 | | S1868 | S 100 | 9 | S1970 | S 57 | 9 | C1928 | C 77 | 9 | C1898 | C 50 | 9 | N2004 | N 64 | 8 |
| 55 | | P1869 | P 100 | 9 | P1971 | P 100 | 9 | P1929 | P 100 | 9 | P1899 | P 88 | 7 | P2005 | P 89 | 8 |
| 56 | | F1870 | F 59 | 6 | F1972 | F 87 | 7 | Y1930 | Y 55 | 6 | Y1900 | Y 65 | 6 | N2006 | K 30 | 2 |
| 57 | | L1871 | L 72 | 6 | M1973 | M 96 | 9 | M1931 | M 40 | 8 | A1901 | A 37 | 4 | Y2007 | F 70 | 7 |
| 58 | | E1872 | R 54 | 4 | K1974 | K 83 | 6 | K1932 | K 65 | 7 | R1902 | K 52 | 7 | A2008 | L 25 | 1 |
| 59 | | Q1873 | C 49 | 5 | T1975 | T 55 | 7 | A1933 | T 49 | 5 | D1903 | D 42 | 5 | T2009 | T 31 | 5 |
| 60 | | P1874 | P 97 | 9 | P1976 | P 99 | 9 | P1934 | P 67 | 6 | P1904 | P 92 | 8 | G2010 | G 40 | 2 |
| 61 | | E1875 | E 26 | 1 | E1977 | E 68 | 6 | E1935 | E 44 | 2 | E1905 | E 71 | 5 | K2011 | D 31 | 2 |
| 62 | | L1876 | R 44 | 4 | E1978 | E 58 | 6 | K1936 | E 31 | 2 | N1906 | N 38 | 4 | P2012 | S 23 | 1 |
| 63 | | F1877 | F 46 | 6 | Y1979 | Y 57 | 6 | L1937 | L 51 | 7 | M1907 | M 65 | 7 | W2013 | W 70 | 5 |
| 64 | | - | - | - | - | - | - | - | - | - | - | - | - | G2014 | G 20 | 3 |
| 65 | | D1878 | D 33 | 3 | K1980 | T 37 | 3 | T1938 | T 24 | 4 | N1908 | N 35 | 5 | - | - | - |
| 66 | | L1879 | V 44 | 7 | C1981 | C 91 | 9 | C1939 | C 49 | 3 | C1909 | C 71 | 5 | - | - | - |
| 67 | | S1880 | G 46 | 8 | G1982 | G 100 | 9 | S1940 | T 23 | 3 | T1910 | T 75 | 7 | S2015 | S 44 | 7 |
| 68 | | I1881 | I 74 | 8 | V1983 | V 80 | 9 | L1941 | L 81 | 8 | H1911 | L 40 | 3 | V2016 | L 51 | 6 |
| 69 | | E1882 | E 46 | 4 | Y1984 | Y 38 | 3 | D1942 | Q 25 | 2 | G1912 | D 33 | 3 | E2017 | E 33 | 2 |
| 70 | | E1883 | E 46 | 7 | G1985 | G 82 | 7 | S1943 | E 40 | 4 | A1913 | A 33 | 1 | A2018 | E 38 | 6 |
| 71 | | M1884 | V 33 | 5 | M1986 | M 46 | 7 | L1944 | L 57 | 5 | M1914 | M 33 | 7 | M2019 | M 82 | 8 |
| 72 | | A1885 | A 82 | 8 | A1987 | A 58 | 8 | T1945 | T 67 | 7 | I1915 | I 50 | 4 | A2020 | A 91 | 9 |
| 73 | | T1886 | K 28 | 2 | A1988 | T 23 | 1 | T1946 | A 33 | 3 | E1916 | D 33 | 1 | S2021 | A 31 | 3 |
| 74 | | I1887 | L 54 | 6 | K1989 | K 43 | 4 | P1947 | P 28 | 6 | S1917 | S 87 | 8 | A2022 | E 20 | 4 |
| 75 | | F1888 | Y 31 | 8 | F1990 | Y 51 | 7 | Y1948 | Y 91 | 8 | F1918 | Y 62 | 7 | Y2023 | Y 100 | 9 |
| 76 | | L1889 | V 36 | 5 | I1991 | I 56 | 7 | L1949 | L 56 | 4 | C1919 | I 37 | 1 | A2024 | A 62 | 7 |
| 77 | | R1890 | A 28 | 5 | E1992 | E 39 | 1 | A1950 | A 37 | 3 | N1920 | N 42 | 3 | D2025 | S 24 | 3 |
| 78 | | T1891 | A 82 | 8 | A1993 | E 89 | 8 | E1951 | E 100 | 9 | E1921 | E 75 | 8 | Y2026 | L 29 | 1 |
| 79 | | I1892 | L 44 | 7 | M1994 | I 45 | 6 | I1952 | I 59 | 7 | I1922 | I 63 | 8 | A2027 | I 62 | 7 |
| 80 | | R1893 | R 46 | 6 | K1995 | K 75 | 6 | R1953 | R 83 | 7 | R1923 | R 73 | 5 | V2028 | S 20 | 1 |
| 81 | | R1894 | K 38 | 3 | A1996 | R 88 | 8 | R1954 | R 100 | 9 | R1924 | R 81 | 7 | K2029 | R 22 | 1 |
| 82 | | I1895 | I 24 | 4 | R1997 | R 99 | 9 | R1955 | R 84 | 8 | R1925 | R 88 | 8 | V2030 | T 38 | 4 |
| 83 | | Q1896 | Q 92 | 9 | Q1998 | Q 98 | 9 | Q1956 | Q 81 | 7 | Q1926 | Q 100 | 9 | A2031 | A 29 | 3 |
| 84 | | P1897 | P 95 | 9 | S1999 | P 91 | 7 | P1957 | P 92 | 8 | P1927 | P 81 | 6 | G2032 | G 27 | 1 |
| 85 | | H1898 | H 26 | 1 | K2000 | K 27 | 1 | T1958 | K 25 | 1 | R1928 | R 19 | 1 | S2033 | S 25 | 2 |
| 86 | | G1899 | G 87 | 8 | G2001 | G 100 | 9 | G1959 | G 92 | 8 | G1929 | G 100 | 9 | R2034 | G 43 | 2 |
| 87 | | P1900 | P 82 | 7 | P2002 | P 100 | 9 | P1960 | P 93 | 8 | P1930 | P 100 | 9 | P2035 | P 55 | 4 |
| 88 | | Y1901 | Y 56 | 7 | Y2003 | Y 100 | 9 | Y1961 | Y 88 | 7 | Y1931 | Y 98 | 8 | V2036 | V 30 | 6 |
| 89 | | L1902 | L 51 | 5 | S2004 | S 25 | 3 | N1962 | N 32 | 3 | H1932 | H 60 | 5 | I2037 | I 66 | 7 |
| 90 | | I1903 | L 46 | 6 | L2005 | L 57 | 3 | L1963 | L 60 | 5 | L1933 | L 73 | 6 | F2038 | L 64 | 6 |
| 91 | | G1904 | G 85 | 8 | A2006 | G 51 | 7 | G1964 | G 83 | 9 | G1934 | G 94 | 8 | G2039 | G 80 | 8 |
| 92 | | G1905 | G 100 | 9 | G2007 | G 100 | 9 | G1965 | G 100 | 9 | G1935 | G 100 | 9 | G2040 | G 100 | 9 |
| 93 | | W1906 | W 46 | 6 | W2008 | W 100 | 9 | W1966 | W 99 | 8 | W1936 | W 100 | 9 | W2041 | W 100 | 9 |
| **94** | | **S1907** | **S 100** | **9** | **S2009** | **S 100** | **9** | **S1967** | **S 100** | **9** | **S1937** | **S 100** | **9** | **S2042** | **S 90** | **9** |
| 95 | | A1908 | G 51 | 8 | A2010 | A 100 | 9 | A1968 | A 100 | 9 | S1938 | A 54 | 8 | F2043 | F 93 | 9 |
| 96 | | G1909 | G 97 | 9 | G2011 | G 99 | 9 | G1969 | G 100 | 9 | G1939 | G 100 | 9 | G2044 | G 100 | 9 |
| 97 | | S1910 | A 41 | 6 | G2012 | G 100 | 9 | G1970 | G 100 | 9 | G1940 | G 100 | 9 | G2045 | G 95 | 9 |
| 98 | | M1911 | M 28 | 6 | V2013 | V 98 | 9 | I1971 | I 77 | 8 | A1941 | I 54 | 7 | V2046 | V 93 | 9 |
| 99 | | Y1912 | Y 31 | 3 | I2014 | I 63 | 8 | C1972 | C 61 | 5 | F1942 | F 52 | 6 | V2047 | V 71 | 8 |
| 100 | | A1913 | A 87 | 9 | A2015 | A 92 | 9 | A1973 | A 100 | 9 | A1943 | A 94 | 9 | G2048 | A 95 | 9 |
| 101 | | Y1914 | Y 92 | 8 | Y2016 | Y 80 | 4 | Y1974 | Y 57 | 4 | Y1944 | Y 67 | 5 | F2049 | F 74 | 7 |
| 102 | | E1915 | E 100 | 9 | E2017 | E 92 | 9 | D1975 | D 53 | 8 | V1945 | A 33 | 7 | E2050 | E 91 | 9 |
| 103 | | V1916 | V 67 | 8 | I2018 | I 41 | 5 | A1976 | A 97 | 9 | V1946 | V 44 | 5 | A2051 | A 30 | 5 |
| 104 | | A1917 | A 49 | 5 | V2019 | V 49 | 7 | A1977 | A 85 | 8 | A1947 | A 94 | 8 | A2052 | A 95 | 9 |
| 105 | | H1918 | R 69 | 7 | N2020 | N 32 | 6 | R1978 | R 70 | 7 | E1948 | Q 46 | 7 | R2053 | R 70 | 7 |
| 106 | | R1919 | Q 56 | 7 | Q2021 | Q 94 | 9 | K1979 | Q 62 | 4 | A1949 | A 25 | 1 | Q2054 | Q 49 | 6 |
| 107 | | L1920 | L 95 | 9 | L2022 | L 96 | 8 | L1980 | L 88 | 8 | L1950 | L 98 | 9 | L2055 | L 91 | 9 |
| 108 | | T1921 | L 31 | 4 | T2023 | T 31 | 4 | V1981 | V 28 | 3 | V1951 | I 58 | 4 | M2056 | M 26 | 3 |
| 109 | | - | - | - | - | - | - | L1982 | L 55 | 1 | - | - | - | - | - | - |
| 110 | | R1922 | A 41 | 4 | K2024 | K 42 | 5 | Q1983 | E 30 | 2 | N1952 | N 31 | 4 | R2057 | A 26 | 4 |
| 111 | | E1923 | A 64 | 7 | A2025 | A 52 | 3 | Q1984 | E 49 | 1 | Q1953 | A 40 | 4 | R2058 | R 19 | 1 |
| 112 | | G1924 | G 90 | 8 | G2026 | G 78 | 5 | G1985 | G 93 | 8 | G1954 | G 100 | 9 | G2059 | G 72 | 6 |
| 113 | | E1925 | E 41 | 7 | E2027 | E 61 | 5 | E1986 | E 73 | 5 | E1955 | E 85 | 6 | V2060 | V 30 | 2 |
| 114 | | T1926 | R 26 | 1 | T2028 | K 28 | 1 | I1987 | V 23 | 1 | E1956 | E 50 | 1 | P2061 | P 19 | 1 |
| 115 | | I1927 | V 69 | 8 | V2029 | V 97 | 9 | V1988 | V 85 | 9 | V1957 | V 98 | 9 | V2062 | V 93 | 9 |
| 116 | | Q1928 | D 21 | 1 | E2030 | E 69 | 5 | E1989 | E 49 | 2 | H1958 | H 29 | 1 | K2063 | K 43 | 4 |
| 117 | | A1929 | G 69 | 6 | N2031 | N 35 | 3 | T1990 | R 49 | 5 | S1959 | S 58 | 6 | G2064 | G 93 | 9 |
| 118 | | L1930 | L 92 | 8 | L2032 | L 96 | 8 | L1991 | L 95 | 9 | L1960 | L 94 | 8 | V2065 | V 56 | 7 |
| 119 | | I1931 | V 41 | 5 | I2033 | I 55 | 5 | L1992 | I 54 | 6 | I1961 | I 52 | 5 | V2066 | V 49 | 6 |
| 120 | | I1932 | L 38 | 4 | I2034 | L 60 | 6 | L1993 | L 96 | 9 | I1962 | L 54 | 7 | L2067 | L 91 | 9 |
| 121 | | L1933 | I 62 | 7 | I2035 | I 87 | 8 | L1994 | L 58 | 7 | I1963 | I 92 | 8 | I2068 | I 86 | 9 |
| **122** | | **D1934** | **D 100** | **9** | **D2036** | **D 99** | **9** | **D1995** | **D 100** | **9** | **D1964** | **D 100** | **9** | **D2069** | **D 100** | **9** |
| 123 | | M1935 | M 71 | 8 | A2037 | A 53 | 8 | T1996 | S 84 | 8 | A1965 | S 56 | 8 | S2070 | S 77 | 9 |
| 124 | | R1936 | R 37 | 7 | P2038 | P 100 | 9 | P1997 | P 100 | 9 | P1966 | P 100 | 9 | P2071 | P 100 | 9 |
| 125 | | A1937 | A 32 | 4 | C2039 | C 89 | 7 | F1998 | N 62 | 8 | I1967 | V 44 | 4 | F2072 | S 28 | 5 |
| 126 | | P1938 | P 89 | 8 | P2040 | P 100 | 9 | P1999 | P 100 | 9 | P1968 | P 100 | 9 | P2073 | P 98 | 9 |
| 127 | | - | - | - | V2041 | V 45 | 3 | I2000 | I 66 | 7 | Q1969 | Q 38 | 1 | V2074 | I 25 | 1 |
| 128 | | S1939 | R 39 | 5 | T2042 | T 38 | 6 | G2001 | G 99 | 9 | A1970 | G 56 | 4 | - | - | - |
| 129 | | L1940 | L 29 | 4 | I2043 | I 56 | 9 | L2002 | L 95 | 9 | M1971 | L 58 | 8 | - | - | - |
| 130 | | I1941 | S 16 | 1 | E2044 | E 75 | 8 | E2003 | E 61 | 8 | E1972 | D 56 | 7 | D2075 | N 48 | 6 |
| 131 | | P1942 | P 62 | 5 | P2045 | P 89 | 8 | K2004 | K 69 | 8 | Q1973 | K 31 | 3 | H2076 | H 70 | 7 |
| 132 | | T1943 | D 16 | 1 | L2046 | L 99 | 9 | L2005 | L 70 | 8 | L1974 | L 100 | 9 | V2077 | V 45 | 6 |
| 133 | | S1944 | A 21 | 1 | P2047 | P 100 | 9 | P2006 | P 97 | 9 | P1975 | P 100 | 9 | P2078 | P 86 | 8 |
| 134 | | I1945 | E 39 | 4 | R2048 | A 52 | 4 | P2007 | P 38 | 2 | R1976 | R 35 | 2 | S2079 | L 75 | 7 |
| 135 | | V1946 | V 31 | 2 | S2049 | S 37 | 7 | R2008 | R 89 | 8 | A1977 | R 40 | 6 | S2080 | S 49 | 8 |
| 136 | | T1947 | T 41 | 6 | L2050 | L 89 | 9 | L2009 | L 51 | 8 | F1978 | F 90 | 8 | N2081 | D 28 | 2 |
| 137 | | T1948 | M 26 | 2 | H2051 | H 100 | 9 | Y2010 | Y 81 | 7 | Y1979 | Y 88 | 7 | E2082 | E 23 | 2 |
| 138 | | D1949 | E 32 | 6 | A2052 | A 38 | 6 | S2011 | D 55 | 3 | E1980 | E 54 | 6 | F2083 | I 42 | 6 |
| 139 | | F1950 | I 29 | 5 | W2053 | W 52 | 7 | F2012 | F 95 | 9 | H1981 | H 71 | 6 | M2084 | I 72 | 8 |
| 140 | | V1951 | F 32 | 4 | F2054 | F 89 | 8 | F2013 | F 49 | 5 | C1982 | C 94 | 7 | A2085 | D 37 | 5 |
| 141 | | D1952 | E 50 | 5 | A2055 | A 51 | 7 | N2014 | N 31 | 4 | N1983 | N 38 | 2 | V2086 | A 33 | 5 |
| 142 | | K1953 | A 18 | 2 | S2056 | S 41 | 5 | S2015 | S 43 | 5 | S1984 | K 29 | 1 | T2087 | V 53 | 7 |
| 143 | | L1954 | I 34 | 4 | I2057 | I 95 | 9 | I2016 | I 28 | 3 | I1985 | I 33 | 3 | A2088 | T 37 | 5 |
| 144 | | G1955 | G 39 | 7 | G2058 | G 100 | 9 | G2017 | G 84 | 6 | G1986 | G 75 | 7 | G2089 | G 26 | 2 |
| 145 | | T1956 | M 16 | 3 | L2059 | L 99 | 9 | L2018 | L 39 | 4 | L1987 | L 55 | 5 | A2090 | L 19 | 1 |
| 146 | | F1957 | F 22 | 1 | L2060 | L 100 | 9 | F2019 | F 95 | 9 | F1988 | F 82 | 7 | F2091 | D 19 | 1 |
| 147 | | E1958 | D 24 | 7 | G2061 | G 98 | 9 | G2020 | G 99 | 9 | A1989 | G 49 | 6 | T2092 | G 19 | 1 |
| 148 | | G1959 | G 67 | 6 | E2062 | T 36 | 6 | E2021 | E 36 | 1 | T1990 | N 29 | 1 | - | - | - |
| 149 | | I1960 | I 50 | 5 | G2063 | G 97 | 8 | G2022 | G 68 | 6 | Q1991 | Q 33 | 5 | - | - | - |
| 150 | | N1961 | N 35 | 6 | D2064 | D 55 | 6 | - | - | - | P1992 | P 30 | 2 | - | - | - |
| 151 | | - | - | - | D2065 | P 39 | 1 | - | - | - | G1993 | G 64 | 5 | - | - | - |
| 152 | | - | - | - | E2066 | A 36 | 1 | - | - | - | A1994 | R 33 | 1 | - | - | - |
| 153 | | - | - | - | - | - | - | - | - | - | S1995 | G 32 | 1 | - | - | - |
| 154 | | - | - | - | - | - | - | - | - | - | P1996 | A 42 | 5 | R2093 | R 19 | 1 |
| 155 | | - | - | - | - | - | - | - | - | - | D1997 | D 47 | 1 | G2094 | S 23 | 3 |
| 156 | | R1962 | R 74 | 7 | A2067 | A 53 | 4 | - | - | - | G1998 | G 56 | 1 | G2095 | G 29 | 1 |
| 157 | | A1963 | N 32 | 5 | A2068 | A 59 | 1 | K2023 | K 27 | 2 | S1999 | S 26 | 1 | R2096 | A 19 | 2 |
| 158 | | R1964 | F 31 | 1 | K2069 | K 29 | 2 | A2024 | Q 20 | 1 | T2000 | T 24 | 1 | T2097 | S 26 | 3 |
| 159 | | D1965 | S 21 | 5 | K2070 | K 70 | 6 | A2025 | A 42 | 4 | E2001 | K 32 | 1 | P2098 | E 19 | 1 |
| 160 | | L1966 | G 36 | 5 | I2071 | I 40 | 5 | P2026 | P 61 | 6 | P2002 | P 62 | 5 | I2099 | I 37 | 5 |
| 161 | | P1967 | L 26 | 3 | P2072 | P 100 | 9 | P2027 | P 97 | 9 | P2003 | P 100 | 9 | G2100 | R 26 | 1 |
| 162 | | E1968 | W 33 | 2 | S2073 | S 34 | 1 | A2028 | D 34 | 2 | S2004 | S 22 | 1 | R2101 | N 16 | 1 |
| 163 | | D1969 | P 31 | 4 | W2074 | W 100 | 9 | W2029 | W 99 | 8 | Y2005 | W 60 | 8 | M2102 | L 56 | 6 |
| 164 | | L1970 | L 23 | 1 | L2075 | L 100 | 9 | L2030 | L 93 | 8 | L2006 | L 98 | 8 | M2103 | V 49 | 6 |
| 165 | | S1971 | S 41 | 5 | - | - | - | - | - | - | - | - | - | - | - | - |
| 166 | | V1972 | D 19 | 1 | - | - | - | - | - | - | - | - | - | - | - | - |
| 167 | | K1973 | K 21 | 1 | - | - | - | - | - | - | - | - | - | - | - | - |
| 168 | | E1974 | T 31 | 6 | - | - | - | - | - | - | - | - | - | - | - | - |
| 169 | | R1975 | K 31 | 5 | L2076 | L 100 | 9 | L2031 | L 57 | 6 | I2007 | I 72 | 8 | W2104 | K 26 | 3 |
| 170 | | A1976 | Q 26 | 1 | P2077 | P 99 | 9 | P2032 | P 46 | 5 | P2008 | P 78 | 6 | K2105 | A 30 | 5 |
| 171 | | H1977 | H 100 | 9 | H2078 | H 100 | 9 | H2033 | H 97 | 9 | H2009 | H 100 | 9 | Q2106 | Q 65 | 8 |
| 172 | | L1978 | L 69 | 7 | F2079 | F 100 | 9 | F2034 | F 100 | 9 | F2010 | F 100 | 9 | L2107 | F 74 | 8 |
| 173 | | M1979 | R 21 | 2 | A2080 | A 72 | 8 | L2035 | L 45 | 5 | T2011 | R 28 | 1 | Q2108 | Q 21 | 3 |
| 174 | | A1980 | A 51 | 7 | A2081 | A 65 | 8 | A2036 | A 73 | 8 | A2012 | A 88 | 8 | Q2109 | A 21 | 1 |
| 175 | | T1981 | T 36 | 5 | S2082 | S 67 | 8 | F2037 | F 92 | 7 | V2013 | T 36 | 4 | N2110 | N 49 | 8 |
| 176 | | C1982 | F 41 | 6 | V2083 | V 60 | 8 | I2038 | I 81 | 7 | V2014 | V 40 | 7 | A2111 | A 53 | 7 |
| 177 | | R1983 | R 38 | 6 | T2084 | T 40 | 5 | D2039 | D 51 | 5 | D2015 | D 52 | 5 | P2112 | R 35 | 5 |
| 178 | | A1984 | A 49 | 6 | A2085 | A 68 | 9 | S2040 | A 40 | 5 | V2016 | V 38 | 7 | L2113 | L 56 | 7 |
| 179 | | L1985 | V 41 | 8 | L2086 | L 100 | 9 | L2041 | L 100 | 9 | M2017 | L 60 | 8 | L2114 | L 95 | 9 |
| 180 | | S1986 | A 26 | 3 | S2087 | S 63 | 8 | D2042 | D 89 | 7 | L2018 | H 42 | 6 | K2115 | G 37 | 3 |
| 181 | | R1987 | A 28 | 3 | N2088 | N 41 | 6 | A2043 | A 36 | 2 | D2019 | D 64 | 3 | T2116 | R 23 | 3 |
| 182 | | Y1988 | Y 87 | 8 | Y2089 | Y 99 | 9 | Y2044 | Y 89 | 8 | Y2020 | Y 100 | 9 | Y2117 | Y 81 | 8 |
| 183 | | D1989 | H 31 | 3 | T2090 | D 34 | 3 | K2045 | K 38 | 2 | K2021 | H 36 | 1 | D2118 | D 23 | 1 |
| 184 | | A1990 | P 69 | 8 | A2091 | A 47 | 8 | A2046 | P 51 | 7 | L2022 | A 58 | 6 | P2119 | P 72 | 7 |
| 185 | | P1991 | P 33 | 1 | E2092 | E 53 | 1 | V2047 | V 41 | 2 | A2023 | A 24 | 1 | R2120 | E 19 | 2 |
| 186 | | A1992 | P 72 | 6 | P2093 | P 63 | 3 | P2048 | P 85 | 6 | P2024 | P 94 | 7 | I2121 | A 28 | 1 |
| 187 | | F1993 | M 41 | 7 | I2094 | I 59 | 6 | L2049 | L 37 | 4 | L2025 | L 74 | 5 | A2122 | S 36 | 4 |
| 188 | | P1994 | P 27 | 1 | P2095 | P 39 | 3 | P2050 | P 40 | 1 | H2026 | P 34 | 1 | - | - | - |
| 189 | | - | - | - | - | - | - | F2051 | F 33 | 1 | - | - | - | - | - | - |
| 190 | | - | - | - | - | - | - | N2052 | N 24 | 3 | - | - | - | - | - | - |
| 191 | | - | - | - | - | - | - | E2053 | D 59 | 5 | - | - | - | - | - | - |
| 192 | | - | - | - | - | - | - | Q2054 | P 34 | 1 | - | - | - | - | - | - |
| 193 | | - | - | - | - | - | - | E2055 | K 56 | 2 | - | - | - | - | - | - |
| 194 | | - | - | - | - | - | - | W2056 | W 54 | 1 | - | - | - | - | - | - |
| 195 | | S1995 | P 32 | 2 | K2096 | K 35 | 1 | K2057 | A 40 | 3 | A2027 | A 44 | 1 | G2123 | S 23 | 4 |
| 196 | | D1996 | G 29 | 3 | E2097 | K 24 | 1 | G2058 | G 31 | 1 | R2028 | G 26 | 1 | G2124 | G 43 | 4 |
| 197 | | R1997 | E 29 | 4 | K2098 | K 65 | 4 | K2059 | K 33 | 2 | R2029 | R 56 | 3 | P2125 | P 26 | 1 |
| 198 | | Q1998 | R 59 | 5 | C2099 | C 42 | 6 | L2060 | A 40 | 3 | M2030 | M 36 | 4 | Y2126 | V 14 | 1 |
| 199 | | P1999 | P 86 | 8 | P2100 | P 93 | 8 | P2061 | P 84 | 8 | P2031 | P 70 | 6 | P2127 | P 70 | 7 |
| 200 | | K2000 | K 21 | 1 | N2101 | K 34 | 1 | K2062 | K 59 | 2 | K2032 | K 48 | 5 | P2128 | R 37 | 5 |
| 201 | | Q2001 | R 36 | 6 | - | - | - | - | - | - | - | - | - | - | - | - |
| 202 | | V2002 | T 67 | 8 | V2102 | V 66 | 8 | T2063 | T 81 | 7 | V2033 | V 62 | 7 | L2129 | L 33 | 7 |
| 203 | | A2003 | A 51 | 5 | M2103 | M 27 | 1 | Y2064 | Y 29 | 3 | G2034 | G 40 | 5 | V2130 | V 60 | 8 |
| 204 | | V2004 | V 51 | 5 | A2104 | A 73 | 6 | L2065 | I 48 | 4 | I2035 | I 80 | 6 | L2131 | L 49 | 7 |
| 205 | | V2005 | I 64 | 8 | I2105 | I 92 | 9 | V2066 | I 44 | 5 | V2036 | I 68 | 7 | L2132 | L 93 | 9 |
| 206 | | W2006 | W 97 | 8 | W2106 | W 100 | 9 | W2067 | W 71 | 7 | W2037 | W 100 | 9 | H2133 | R 77 | 8 |
| 207 | | A2007 | A 79 | 9 | C2107 | C 79 | 9 | A2068 | A 99 | 9 | A2038 | A 100 | 9 | N2134 | S 72 | 8 |
| 208 | | L2008 | K 28 | 2 | E2108 | E 55 | 8 | K2069 | K 36 | 7 | A2039 | C 34 | 1 | Q2135 | R 26 | 1 |
| 209 | | L2009 | K 59 | 6 | D2109 | D 90 | 7 | D2070 | D 92 | 8 | D2040 | E 58 | 4 | E2136 | E 42 | 5 |
| 210 | | G2010 | G 87 | 8 | G2110 | G 100 | 9 | G2071 | G 100 | 9 | T2041 | T 42 | 6 | G2137 | G 58 | 6 |
| 211 | | L2011 | M 44 | 7 | V2111 | V 93 | 9 | V2072 | V 70 | 7 | V2042 | V 56 | 7 | I2138 | F 35 | 2 |
| 212 | | D2012 | V 23 | 6 | C2112 | C 85 | 8 | C2073 | C 96 | 8 | M2043 | M 36 | 4 | P2139 | D 33 | 2 |
| 213 | | N2013 | D 28 | 4 | H2113 | K 34 | 5 | P2074 | K 58 | 5 | D2044 | D 90 | 6 | P2140 | P 23 | 4 |
| 214 | | R2014 | R 56 | 6 | L2114 | L 44 | 4 | K2075 | K 29 | 4 | E2045 | G 50 | 6 | D2141 | P 21 | 1 |
| 215 | | P2015 | L 31 | 3 | P2115 | P 84 | 6 | P2076 | P 73 | 4 | R2046 | V 33 | 1 | A2142 | G 23 | 1 |
| 216 | | D2016 | D 26 | 4 | T2116 | T 41 | 4 | G2077 | D 34 | 1 | D2047 | N 27 | 1 | F2143 | V 27 | 1 |
| 217 | | A2017 | G 23 | 1 | D2117 | D 100 | 9 | D2078 | D 62 | 4 | A2048 | A 35 | 4 | L2144 | L 35 | 3 |
| 218 | | P2018 | I 16 | 1 | P2118 | P 92 | 8 | P2079 | P 75 | 5 | P2049 | P 86 | 5 | P2145 | C 15 | 1 |
| 219 | | I2019 | L 58 | 4 | - | - | - | - | - | - | - | - | - | - | - | - |
| 220 | | A2020 | A 58 | 5 | - | - | - | - | - | - | - | - | - | - | - | - |
| 221 | | S2021 | D 32 | 1 | - | - | - | - | - | - | - | - | - | - | - | - |
| 222 | | M2022 | M 26 | 2 | - | - | - | - | - | - | - | - | - | - | - | - |
| 223 | | G2023 | G 74 | 6 | - | - | - | - | - | - | - | - | - | - | - | - |
| 224 | | R2024 | I 47 | 6 | R2119 | R 88 | 9 | W2080 | R 77 | 6 | K2050 | K 70 | 1 | Y2146 | G 41 | 2 |
| 225 | | P2025 | P 57 | 4 | P2120 | P 98 | 9 | P2081 | P 80 | 7 | - | - | - | P2147 | D 24 | 1 |
| 226 | | G2026 | T 40 | 5 | D2121 | D 38 | 5 | E2082 | E 77 | 6 | - | - | - | V2148 | V 40 | 6 |
| 227 | | L2027 | E 47 | 4 | P2122 | P 89 | 8 | P2083 | P 30 | 3 | - | - | - | P2149 | P 33 | 6 |
| 228 | | D2028 | S 28 | 2 | Y2123 | Y 46 | 8 | A2084 | R 34 | 4 | - | - | - | - | - | - |
| 229 | | I2029 | Y 44 | 3 | P2124 | P 99 | 9 | E2085 | P 60 | 6 | - | - | - | - | - | - |
| 230 | | G2030 | P 63 | 7 | T2125 | T 38 | 4 | D2086 | D 95 | 8 | - | - | - | - | - | - |
| 231 | | - | - | - | - | - | - | G2087 | G 91 | 7 | - | - | - | - | - | - |
| 232 | | - | - | - | - | - | - | S2088 | S 74 | 7 | - | - | - | - | - | - |
| 233 | | K2031 | K 64 | 6 | - | - | - | K2089 | P 35 | 1 | - | - | - | - | - | - |
| 234 | | S2032 | N 27 | 1 | - | - | - | D2090 | D 96 | 9 | - | - | - | - | - | - |
| 235 | | M2033 | M 50 | 3 | - | - | - | P2091 | P 84 | 7 | M2051 | M 38 | 4 | - | - | - |
| 236 | | Y2034 | S 33 | 3 | G2126 | G 55 | 8 | R2092 | R 88 | 8 | K2052 | E 41 | 5 | - | - | - |
| 237 | | E2035 | E 50 | 2 | H2127 | H 55 | 9 | E2093 | E 74 | 8 | G2053 | G 80 | 6 | - | - | - |
| 238 | | M2036 | G 47 | 5 | A2128 | A 55 | 9 | M2094 | M 95 | 9 | M2054 | M 75 | 7 | - | - | - |
| 239 | | N2037 | F 44 | 3 | - | - | - | - | - | - | - | - | - | - | - | - |
| 240 | | L2038 | M 61 | 8 | - | - | - | - | - | - | - | - | - | - | - | - |
| 241 | | D2039 | E 51 | 7 | - | - | - | - | - | - | - | - | - | - | - | - |
| 242 | | E2040 | D 59 | 6 | - | - | - | - | - | - | - | - | - | - | - | - |
| 243 | | F2041 | P 43 | 4 | - | - | - | - | - | - | - | - | - | - | - | - |
| 244 | | E2042 | E 31 | 4 | - | - | - | - | - | - | - | - | - | - | - | - |
| 245 | | R2043 | L 45 | 4 | - | - | - | - | - | - | - | - | - | - | - | - |
| 246 | | Y2044 | G 74 | 7 | - | - | - | - | - | - | - | - | - | - | - | - |
| 247 | | F2045 | A 26 | 4 | - | - | - | - | - | - | - | - | - | - | - | - |
| 248 | | N2046 | V 29 | 4 | - | - | - | - | - | - | - | - | - | - | - | - |
| 249 | S2047 | A 45 | 6 | L2129 | L 40 | 6 | V2095 | L 26 | 1 | H2055 | K 59 | 8 | R2150 | P 28 | 1 |  |
| 250 | W2048 | W 92 | 8 | F2130 | F 51 | 8 | W2096 | W 100 | 9 | F2056 | F 100 | 9 | W2151 | W 77 | 8 |  |
| 251 | F2049 | F 43 | 7 | L2131 | L 100 | 9 | L2097 | L 99 | 9 | M2057 | L 61 | 9 | M2152 | L 86 | 8 |  |
| 252 | Y2050 | L 32 | 4 | L2132 | L 91 | 8 | L2098 | L 92 | 8 | I2058 | T 41 | 7 | S2153 | S 40 | 6 |  |
| 253 | G2051 | P 51 | 6 | D2133 | D 47 | 7 | S2099 | N 62 | 6 | Q2059 | E 47 | 4 | E2154 | D 72 | 8 |  |
| 254 | R2052 | K 32 | 6 | N2134 | N 86 | 9 | N2100 | N 68 | 7 | K2060 | K 61 | 3 | K2155 | R 67 | 8 |  |
| 255 | R2053 | R 43 | 8 | R2135 | R 98 | 9 | R2101 | R 100 | 9 | R2061 | R 100 | 9 | G2156 | G 100 | 7 |  |
| 256 | Q2054 | A 22 | 2 | T2136 | T 70 | 5 | T2102 | T 69 | 8 | T2062 | T 67 | 5 | T2157 | S 37 | 4 |  |
| 257 | - | - | - | - | - | - | - | - | - | - | - | - | D2158 | D 53 | 6 |  |
| 258 | - | - | - | - | - | - | - | - | - | - | - | - | P2159 | P 44 | 4 |  |
| 259 | Q2055 | D 65 | 6 | D2137 | D 98 | 9 | D2103 | D 74 | 7 | E2063 | D 78 | 8 | C2160 | R 26 | 2 |  |
| 260 | F2056 | L 51 | 7 | F2138 | F 91 | 9 | L2104 | F 64 | 6 | F2064 | F 94 | 8 | L2161 | T 19 | 1 |  |
| 261 | G2057 | G 100 | 9 | G2139 | G 78 | 7 | G2105 | G 65 | 8 | G2065 | G 75 | 7 | L2162 | A 40 | 5 |  |
| 262 | T2058 | P 74 | 8 | P2140 | P 65 | 6 | P2106 | P 57 | 6 | P2066 | P 75 | 8 | A2163 | V 49 | 7 |  |
| 263 | N2059 | N 87 | 9 | N2141 | N 97 | 9 | N2107 | N 74 | 6 | D2067 | D 41 | 5 | D2164 | A 37 | 4 |  |
| 264 | G2060 | G 97 | 9 | R2142 | G 45 | 8 | G2108 | G 72 | 7 | G2068 | G 73 | 5 | D2165 | G 49 | 4 |  |
| 265 | W2061 | W 97 | 8 | W2143 | W 100 | 9 | W2109 | W 100 | 9 | W2069 | W 100 | 9 | W2166 | W 100 | 9 |  |
| 266 | E2062 | D 49 | 6 | D2144 | D 64 | 7 | D2110 | D 68 | 6 | D2070 | D 35 | 6 | S2167 | E 74 | 7 |  |
| 267 | D2063 | K 33 | 2 | E2145 | E 41 | 6 | T2111 | T 35 | 4 | T2071 | T 35 | 1 | G2168 | S 35 | 1 |  |
| 268 | L2064 | L 38 | 4 | Y2146 | L 46 | 5 | L2112 | L 72 | 7 | I2072 | L 45 | 6 | L2169 | L 72 | 6 |  |
| 269 | L2065 | V 59 | 5 | L2147 | L 81 | 7 | V2113 | V 50 | 7 | M2073 | F 49 | 5 | V2170 | V 47 | 7 |  |
| 270 | G2066 | G 79 | 7 | D2148 | D 45 | 5 | G2114 | G 100 | 9 | P2074 | P 98 | 9 | G2171 | G 66 | 7 |  |
| 271 | - | - | - | V2149 | K 19 | 1 | K2115 | P 38 | 1 | G2075 | G 88 | 7 | - | - | - |  |
| 272 | D2067 | G 23 | 2 | N2150 | E 45 | 1 | E2116 | E 33 | 1 | A2076 | A 53 | 5 | A2172 | G 26 | 1 |  |
| 273 | H2068 | E 20 | 1 | K2151 | K 53 | 5 | N2117 | N 70 | 6 | S2077 | E 27 | 1 | S2173 | P 26 | 1 |  |
| 274 | I2069 | V 37 | 6 | F2152 | M 37 | 5 | I2118 | I 40 | 4 | F2078 | I 43 | 5 | I2174 | V 47 | 7 |  |
| 275 | A2070 | L 32 | 1 | R2153 | K 22 | 1 | G2119 | G 90 | 7 | D2079 | D 35 | 1 | K2175 | K 30 | 3 |  |
| 276 | V2071 | C 55 | 7 | T2154 | F 23 | 1 | G2120 | G 29 | 1 | I2080 | I 33 | 3 | V2176 | V 58 | 7 |  |
| 277 | - | - | - | - | - | - | I2121 | I 55 | 6 | V2081 | V 27 | 1 | - | - | - |  |
| 278 | - | - | - | - | - | - | T2122 | E 26 | 3 | - | - | - | - | - | - |  |
| 279 | Y2072 | L 32 | 1 | R2155 | R 44 | 4 | V2123 | V 58 | 7 | R2082 | R 51 | 4 | I2177 | L 51 | 3 |  |
| 280 | T2073 | S 34 | 5 | H2156 | H 46 | 6 | I2124 | I 40 | 5 | A2083 | A 37 | 5 | H2178 | D 67 | 7 |  |
| 281 | V2074 | V 66 | 6 | M2157 | M 77 | 8 | H2125 | E 51 | 3 | D2084 | E 61 | 6 | L2179 | I 79 | 8 |  |
| 282 | N2075 | D 39 | 3 | P2158 | P 49 | 4 | D2126 | D 36 | 2 | G2085 | G 71 | 4 | P2180 | P 84 | 8 |  |
| 283 | G2076 | A 53 | 7 | G2159 | G 99 | 9 | A2127 | A 73 | 9 | A2086 | A 67 | 7 | G2181 | G 98 | 9 |  |
| 284 | D2077 | D 89 | 8 | N2160 | N 92 | 9 | N2128 | N 91 | 9 | N2087 | N 71 | 8 | T2182 | N 51 | 7 |  |
| **285** | **H2078** | **H 95** | **9** | **H2161** | **H 100** | **9** | **H2129** | **H 99** | **9** | **H2088** | **H 100** | **9** | **H2183** | **H 100** | **9** |  |
| 286 | F2079 | F 50 | 6 | F2162 | F 100 | 9 | F2130 | F 99 | 9 | F2089 | F 94 | 7 | F2184 | F 95 | 9 |  |
| 287 | S2080 | S 54 | 7 | S2163 | S 55 | 8 | T2131 | T 68 | 7 | T2090 | S 57 | 8 | T2185 | E 44 | 5 |  |
| 288 | M2081 | M 51 | 6 | M2164 | M 99 | 9 | M2132 | M 89 | 8 | L2091 | M 65 | 8 | T2186 | P 50 | 5 |  |
| 289 | M2082 | P 28 | 5 | M2165 | M 97 | 9 | T2133 | M 50 | 8 | M2092 | M 96 | 9 | F2187 | F 100 | 9 |  |
| 290 | C2083 | T 23 | 3 | H2166 | K 45 | 6 | K2134 | R 34 | 3 | Q2093 | R 30 | 1 | A2188 | E 26 | 4 |  |
| 291 | P2084 | P 74 | 6 | G2167 | G 52 | 1 | G2135 | G 74 | 7 | K2094 | G 52 | 4 | T2189 | P 26 | 2 |  |
| 292 | P2085 | P 51 | 6 | D2168 | P 43 | 3 | E2136 | P 49 | 3 | E2095 | E 52 | 1 | P2190 | A 30 | 1 |  |
| 293 | Y2086 | H 22 | 1 | Y2169 | H 30 | 2 | K2137 | K 34 | 4 | H2096 | H 44 | 1 | H2191 | N 70 | 8 |  |
| 294 | A2087 | V 67 | 7 | V2170 | A 46 | 4 | A2138 | A 59 | 7 | V2097 | A 63 | 8 | L2192 | V 45 | 6 |  |
| 295 | S2088 | H 33 | 2 | S2171 | K 46 | 1 | K2139 | K 30 | 1 | S2098 | S 26 | 1 | G2193 | A 18 | 1 |  |
| 296 | E2089 | L 32 | 1 | Q2172 | Q 27 | 1 | E2140 | E 38 | 2 | I2099 | K 30 | 1 | A2194 | E 23 | 1 |  |
| 297 | - | - | - | T2173 | T 25 | 2 | - | - | - | - | - | - | - | - | - |  |
| 298 | - | - | - | T2174 | T 50 | 3 | - | - | - | - | - | - | - | - | - |  |
| 299 | V2090 | L 66 | 4 | L2175 | L 83 | 4 | L2141 | L 59 | 7 | I2100 | L 55 | 5 | V2195 | V 49 | 5 |  |
| 300 | G2091 | G 47 | 3 | S2176 | G 74 | 3 | A2142 | S 32 | 5 | S2101 | S 39 | 5 | T2196 | S 63 | 8 |  |
| 301 | D2092 | E 26 | 2 | P2177 | K 28 | 1 | T2143 | A 37 | 1 | D2102 | D 28 | 1 | Q2197 | Q 15 | 2 |  |
| 302 | I2093 | V 29 | 2 | Y2178 | F 47 | 1 | F2144 | F 63 | 5 | L2103 | L 48 | 1 | A2198 | Q 35 | 4 |  |
| 303 | V2094 | I 27 | 5 | N2179 | I 48 | 2 | M2145 | I 59 | 6 | I2104 | I 76 | 8 | L2199 | L 54 | 7 |  |
| 304 | I2095 | E 30 | 1 | D2180 | R 56 | 4 | K2146 | R 37 | 2 | D2105 | R 33 | 5 | V2200 | R 21 | 2 |  |
| 305 | E2096 | E 24 | 1 | D2181 | E 60 | 3 | N2147 | E 22 | 1 | R2106 | R 33 | 1 | D2201 | E 33 | 3 |  |
| 306 | T2097 | A 38 | 5 | N2182 | A 51 | 5 | A2148 | A 66 | 5 | V2107 | V 39 | 6 | G2202 | A 73 | 8 |  |
| 307 | V2098 | F 34 | 6 | L2183 | L 72 | 3 | L2149 | M 49 | 4 | M2108 | L 57 | 5 | C2203 | C 61 | 6 |  |
| 308 | T2099 | E 40 | 1 | T2184 | K 24 | 1 | G2150 | G 25 | 1 | A2109 | A 28 | 1 | A2204 | A 15 | 1 |  |

*: MAX shows the % for the most animo-acid residues found in position in the MSA.

**: Evolutionary conservation scores of the residues, analyzed by ConSurf (9, conserved; 1, variable).

The blue block, 27 CLR sites in 3ILS PDB crystal structure (TE domain of *Aspergillus parasiticus* PksA). The red colored words, catalytic sites in TE domains.
